# *Homo sapiens*-specific evolution unveiled by ancient southern African genomes

**DOI:** 10.1038/s41586-025-09811-4

**Published:** 2025-12-03

**Authors:** Mattias Jakobsson, Carolina Bernhardsson, James McKenna, Nina Hollfelder, Mario Vicente, Hanna Edlund, Alexandra Coutinho, Per Sjödin, James Brink, Bernhard Zipfel, Helena Malmström, Marlize Lombard, Carina M. Schlebusch

**Affiliations:** 1 Human Evolution, Department of Organismal Biology, Uppsala University, Uppsala, Sweden; 2 Palaeo-Research Institute, University of Johannesburg, Johannesburg, South Africa; 3 SciLife Lab, Uppsala, Sweden; 4 Population Genetics, Institute of Marine Research, Tromsø, Norway; 5 Department of Immunology, Genetics and Pathology, Rudbeck Laboratory, Uppsala University, Uppsala, Sweden; 6 Centre for Palaeogenetics, Stockholm University, Stockholm, Sweden; 7 Florisbad Quaternary Research Department, National Museum Bloemfontein, Bloemfontein, South Africa; 8 Evolutionary Studies Institute, University of the Witwatersrand, Braamfontein, South Africa

**Keywords:** Biological anthropology, Evolutionary biology, Genetic variation, Evolutionary genetics

## Abstract

*Homo sapiens* evolved hundreds of thousands of years ago in Africa, later spreading across the globe^1^, but the early evolutionary process is debated^2–6^. Here we present whole-genome sequencing data for 28 ancient southern African individuals, including six individuals with 25× to 7.2× genome coverage, dated to between 10,200 and 150 calibrated years before present (cal. bp). All ancient southern Africans dated to more than 1,400 cal. bp show a genetic make-up that is outside the range of genetic variation in modern-day humans (including southern African Khoe-San people, although some retain up to 80% ancient southern African ancestry), manifesting in a large fraction of *Homo sapiens*-specific variants that are unique to ancient southern Africans. *Homo sapiens*-specific variants at amino acid-altering sites fixed for all humans—which are likely to have evolved rapidly on the *Homo sapiens* branch—were enriched for genes associated with kidney function. Some *Homo sapiens*-specific variants fixed in ancient southern Africans—which are likely to have adapted rapidly on the southern African branch—were enriched for genes associated with protection against ultraviolet light. The ancient southern Africans show little spatiotemporal stratification for 9,000 years, consistent with a large, stable Holocene population transcending archaeological phases. While southern Africa served as a long-standing geographical refugium, there is outward gene flow over 8,000 years ago; however, inward gene flow manifests only after around 1,400 years ago. The ancient genomes reported here are therefore key to the evolution of *Homo sapiens*, and are important for advancing our understanding of human genomic variation.

## Abstract (web summary)

The genomes of 28 ancient southern African individuals dated to between 10,200 and 150 years before present offer insights into the evolution of *Homo sapiens.*

## Main

Genetic, anthropological and archaeological studies support an African origin of *Homo sapiens*^1^, but the evolutionary process is debated based on fossils, archaeology and genetics^2–7^, with Africa harbouring the greatest human genetic diversity^8,9^, and southern and central African hunter-gatherer groups displaying some of the deepest diverging *Homo sapiens* lineages^7,10–12^. Population stratification between southern Africa (the region south of the Zambezi River) and the rest of Africa probably existed for at least 300 thousand years (kyr)^4,5,7,13^, perhaps up to a million years^6^. Such deep stratification may result from admixture with an unknown archaic African group predating the divergence of *Homo sapiens* from Neandertals and Denisovans^1,5^, and/or from isolation from other groups.

All investigated modern-day central and southern African Indigenous groups show substantial mixing with western and eastern Africans^4,7,10,14–17^, following the large-scale migrations starting 5 thousand years ago (ka) that veil more ancient events^1^, making it difficult to assess deep human evolutionary history using genomic data from modern-day people. By investigating variation among individuals living before recent large-scale population movements/admixture, palaeogenomic approaches overcome this limitation. Although they are restricted to few individuals and/or single archaeological sites with limited genomic data, these studies show longstanding stratification of ancient ancestries in eastern^18–20^, western^21^, northern^22,23^ and southern Africa^4,17,19^.

**Fig. 1:**
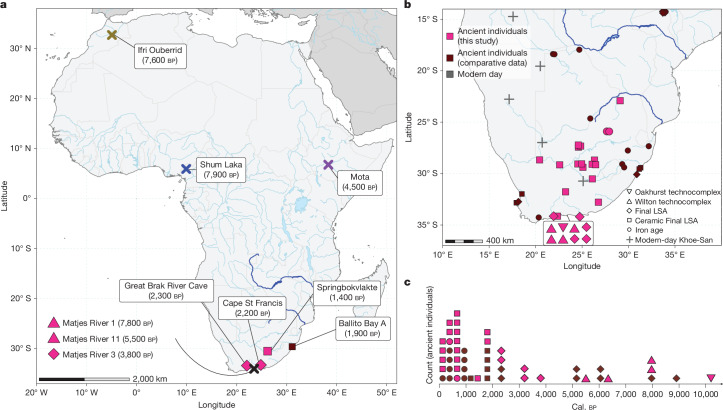
Archaeological sites and dates of sampled and comparative individuals. **a**, All ancient African individuals from >1 ka with complete sequenced genomes (>7× coverage). The seven complete ancient genomes from southern Africa (Matjes River 1, 3 and 11, Great Brak River Cave, Cape St Francis, Springbokvlakte and Ballito Bay A) are studied in detail. The symbol shape indicates archaeological context for southern Africa (see the legend in **b**). The Zambezi and Limpopo rivers are highlighted in blue. **b**, Ancient African individuals with genomic data south of the 14th latitude. Archaeological context is given by the shape of the symbol. LSA, Later Stone Age. **c**, The distribution of sampled and comparative individuals across dates. Maps were created with Natural Earth in R, using vector maps from the package rnaturalearthdata.

We sequenced the genomes of 28 ancient individuals from south of the Limpopo River (South Africa), all dating to the Holocene epoch, with Later Stone Age and Iron Age archaeological affiliations (Fig. 1 and Supplementary Information 1.1). Sampling was geographically and temporally broad (Fig. 1c), with remains recovered from sites across the southern and central parts of South Africa (Fig. 1b). From Matjes River on the southern coast, we sequenced the genomes of individuals spanning around 8 kyr (10,200–2,330 cal. bp; Supplementary Information 1.2).

The sequenced ancient individuals (Fig. 1b) range in coverage from 25.1- to 0.002-fold, with six individuals (Fig. 1a) having over 7.2-fold coverage (Extended Data Table 1). Radiocarbon dates and dietary isotopes were generated for most individuals (Extended Data Table 1, Supplementary Information 2.2 and 3.1, Supplementary Fig. 1 and Supplementary Data 1 and 2). Chronologically, the data cover five archaeological phases—Oakhurst (*n* = 1), Wilton (*n* = 4), Final Later Stone Age (*n* = 3), Ceramic Final Later Stone Age (*n* = 18)^24^ (Supplementary Information 1.1) and Iron Age (*n* = 2), spanning between 10.2 ka and a few hundred years ago. To assess the relationship between the ancient southern Africans (Supplementary Data 1 and 5) and other groups, we co-analysed our data with relevant ancient and modern-day Africans, as well as relevant ancient and/or modern-day Europeans, Asians, Americans and Oceanians (hereafter collectively referred to as non-Africans; Supplementary Data 7 and 8).

Most ancient southern Africans (25 out of 28) carried haplotypes belonging to the mitochondrial L0d haplogroup (Extended Data Table 1), which is common among modern-day Khoe-San individuals^25^ (Supplementary Information 1.3). The oldest individual (Matjes River 6) and four other individuals (Great Brak River Cave, Cape St Francis and two from Ballito Bay^4^) carried a Y chromosome with haplogroup A1b1b2a, which is also common among modern-day Khoe-San individuals^26^. Two individuals who lived ≤500 years ago displayed a Y chromosome with haplogroup E1b1b1, which is common among eastern Africans and modern-day Khoe-San people^26^, consistent with recent gene flow into southern Africa with eastern African pastoralists^4,19^. Variants for high skin pigmentation, brown eyes and non-lactase-persistence were fixed among the seven complete ancient southern African genomes (Fig. 1a and Supplementary Data 6). None of these carried the Duffy-null variant that is protective against Malaria (rs2814778) or the G-variant at rs73885319 in the *APOL1* gene that is protective against sleeping sickness (Supplementary Data 6), even though these variants were present in the region around 500 years ago among individuals with western African ancestry^4^.

## Unique ancient southern African ancestry

**Fig. 2:**
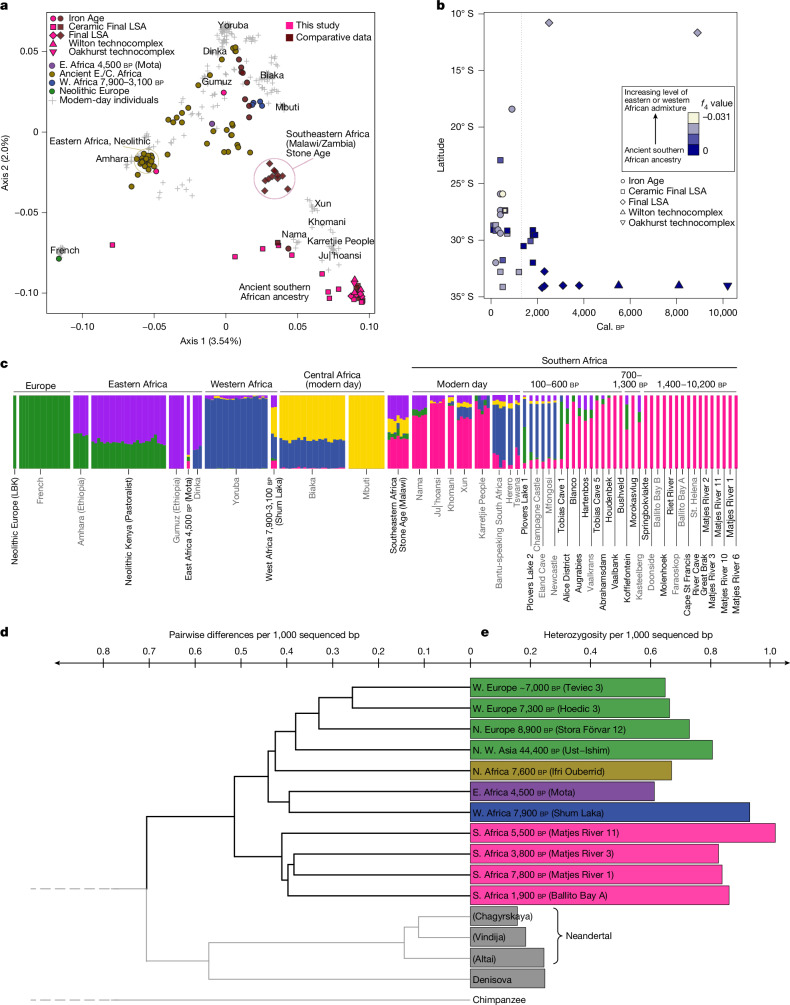
Characterization of the genomic variation in modern-day and ancient Africa. **a**, PCoA of the 28 ancient southern Africans in this study (pink) and all comparative ancient individuals with genetic information across sub-Saharan Africa (modern-day individuals from southern, southeastern, eastern (E), central (C) and western (W) Africa; individual labels are shown in Extended Data Fig. 1). **b**, Gene flow (estimated by two different *f*_4_ tests) from an eastern or western African source into the ancient southern Africans, plotted against years bp and latitude south of the Equator. The vertical dotted line marks 1,300 cal. bp, when admixed individuals first appear. **c**, Estimated ancestry, assuming five ancestry components. The results assuming between two and ten ancestry components are provided in Extended Data Fig. 2 and Supplementary Fig. 6. **d**, Pairwise genetic differences visualized using a hierarchical clustering algorithm (UPGMA) among all pairs of ancient (>1 ka) high-quality genomes (>10×) from Africa and a selection of comparative high-quality ancient genomes from elsewhere (including three Neandertals and a Denisovan individual). bp, base pair. **e**, Genetic diversity (heterozygosity) for ancient high-quality (>10×) genomes.

**Extended Data Fig. 1:**
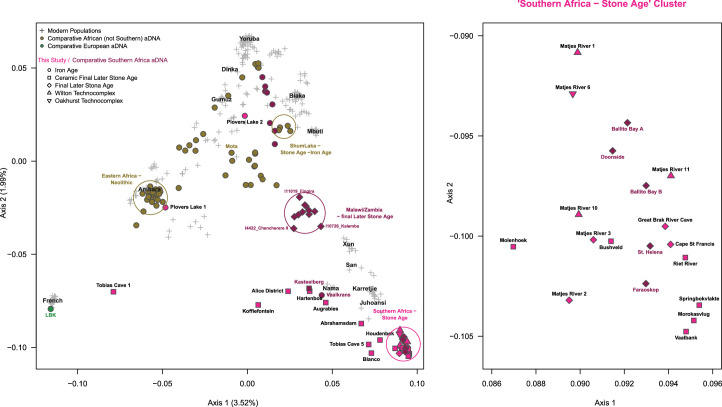
Principal coordinate analysis of ancient and modern-day Africans. Left: Axis 1 vs axis 2 corresponding to the plot in Fig. 2a with specific individuals labelled. Right: Zoom-in of the lower-right corner of the axis 1 and axis 2 space.

Model-free (principal coordinate analysis, PCoA) and model-based approaches (Supplementary Information 2) demonstrate (Fig. 2a and Extended Data Fig. 1) that the first two dimensions (explaining the most genetic variation) form a V-shaped pattern of non-Africans at one end of the distribution, through a gradient of eastern Africans (modern-day and ancient) towards western and central Africans in a second end of the distribution. Indigenous southern Africans form the third end^7,8,27^. Notably, many of the ancient southern Africans (including all individuals between 10.2 ka and 1.4 ka), fall outside the range of genetic variation among modern-day individuals (including Khoe-San groups), and form an extreme end of human genetic variation (at axis 1 and axis 2). This pattern is veiled if the ancient genomes are projected on top of axes of variation built from modern-day genomes and/or ascertained/captured single-nucleotide polymorphisms (SNPs)^17^ (Supplementary Information 2.7 and 3.8–9 and Supplementary Figs. 2, 3 and 9). The ancient western African individuals from Shum Laka dating to 7.9–3.1 ka (ref. ^21^) are located close to modern-day central African rainforest foragers (for example, Biaka, Mbuti) and western Africans. Ancient eastern^18^ and east-central African individuals^20,28^ are distributed among modern-day individuals from eastern, western and central Africa, with ancient eastern African Neolithic pastoralists^28^ clustering with the modern-day eastern African Amhara people. Ancient individuals from southeastern Africa dated to between 8,000 and a few hundred years ago^19,20^ (Malawi, Zambia) cluster in-between eastern Africans (ancient and modern day) and ancient southern Africans.

**Fig. 3:**
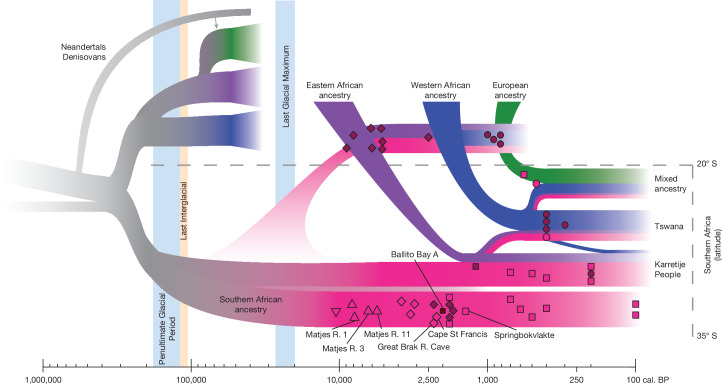
Graphical representation of the inferred human population history of southern Africa. The summary of observations from the ancient genomes in this study that are shown in Figs. 1b,c and 2a–c. Individual symbols correspond to the symbols in Fig. 1b. R., river.

**Extended Data Fig. 2:**
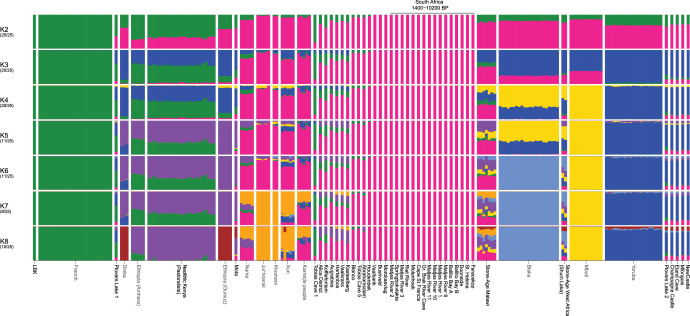
Estimated ancestry fractions assuming between 2 and 8 ancestry components (K). Numbers in parenthesis on the y-axis displays the support for the displayed ancestry estimation (the number of repeated iterations of the ancestry-estimation that resulted very similar solutions).

**Extended Data Fig. 3:**
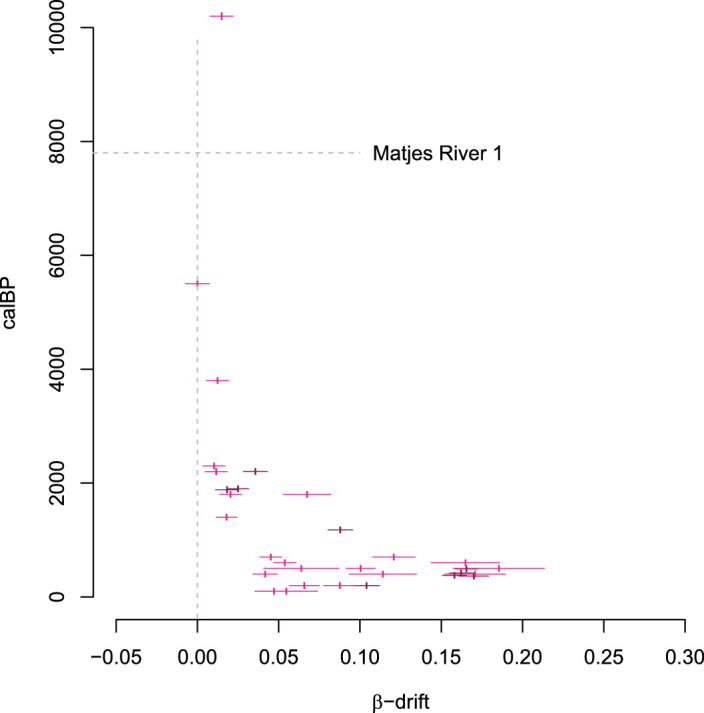
Population continuity. “Anchor”-test for population continuity from Matjes River 1 man, who lived 7.8 kya. The test-statistic (*β*-drift, and normalized against Matjes River 11, who lived 5.5 kya) will be zero for continuous populations, and positive for gene-flow from any other genetically differentiated population. Horizontal lines indicate 95% confidence interval (CI) of the estimate of *β*-drift.

Assuming two ancestry components (Supplementary Information 2.5 and Supplementary Data 7 and 8), one was anchored among the ancient southern Africans (whose genome displays 100% of this ancestry component) and the other among non-Africans (ancient and modern-day Europeans). All of the other individuals (including modern-day and ancient western, eastern and central Africans) were distributed with varying fractions of these ancestry components (Extended Data Fig. 2 and Supplementary Fig. 6), reiterating the unique position of ancient southern Africans. Assuming 3–5 ancestry components (Fig. 2c and Extended Data Fig. 2), the genomes of individuals from western Africa, central Africa and eastern Africa were largely assigned to these ancestry components. Assuming larger numbers of ancestry components typically separated out individual populations (Extended Data Fig. 2 and Supplementary Fig. 6), representing finer-grained population stratification. Assuming five ancestry components (Fig. 2c), the genomes of all ancient southern African individuals between 10,200 and 1,400 cal. bp consist completely of the ‘ancient southern African ancestry component’ (pink), with no indications of admixture (Fig. 3 and Extended Data Fig. 6). For these individuals, there is no indication of temporal genetic stratification (*r*^2^ = 0.02, *P* = 0.06), and low levels of spatial stratification (*r*^2^ = 0.07, *P* = 0.001, Supplementary Information 3.8 and Supplementary Figs. 7 and 8) despite spanning around 9 kyr and a vast landscape (Fig. 1b). Using the oldest high-coverage individual (Matjes River 1) as the anchor individual in a test based on the fraction of shared derived variants to measure population continuity (Supplementary Information 2.16), we find a pattern of population continuity in southern Africans spanning 7 kyr at least (Extended Data Fig. 3), although all individuals older than around 3 ka come from Matjes River.

Average population divergences between individuals representing the ancient southern African group (7 individuals with >7.2-fold genome coverage) to any other individual (ancient and modern-day western, eastern, central, northern Africans and non-Africans) were estimated to around 310–240 ka using a two-by-two site-frequency spectra approach (Supplementary Information 2.17 and Supplementary Data 15–31). Although the exact calibration of chronological population divergence-time estimates depends on model assumptions, mutation-rate assumptions and generation time^5^, these estimates recapitulate findings in which the divergence between ancient southern Africans and all other groups captures the deepest population split-time at around 300 ka (refs. ^4,5,7,11,13,16^) (see also Supplementary Fig. 11 for a comparison to modern-day Khoe-San individuals). This approximately 300 ka population-divergence-time estimate is not caused by a deeper partial archaic admixture event per se, but it does not negate such an event either^6,29^ (Supplementary Information 3.11 and Supplementary Figs. 12 and 13).

We further found that the genetic affinity between ancient southern Africans and eastern Africans (ancient and modern day) is similar to the genetic affinity between ancient southern Africans and western Africans (ancient and modern day; for example, *f*_4_[Denisova, Matjes River 1, Mota, Shum Laka] ≈ 0; Supplementary Fig. 5), suggesting that detectable gene flow from either of these groups was unlikely since around 150–200 ka (the time that marks the divergence between western and eastern Africans; (Supplementary Information 3.5–3.6 and Supplementary Data 25).

## Southern ancestry further north

However, the southern African genetic component was present further north by the mid-Holocene^19,20,30^. Individuals at 11–12° S (current-day Malawi, Zambia) display a mixture of eastern and southern African ancestry by 8 ka (refs. ^19,20^), but no individual with a majority of southern African ancestry has been found in this area. Modern-day Juǀ’hoansi northern San traditionally living around 18–25° S (Botswana, Angola, Namibia), also show eastern African admixture (11%) that may have occurred at the onset of the Holocene^13^. South of 15° S, we detect admixture from eastern and western African sources only from around 1.3 ka (Fig. 2b and Extended Data Fig. 6), but note that few humans dated to >1.3 ka have been palaeogenetically investigated from 10–35° S.

## Gene flow into the south since 1.4 ka

Among the three individuals who lived between 1.3 and 0.7 ka, two (Koffiefontein, Kasteelberg) show a distinct ancestry component matching eastern African Neolithic pastoralists (Fig. 2c (purple and green)), demonstrating that this gene flow extended over a large area. Among the southern Africans living between 0.6 and 0.1 ka, we note: (1) two individuals (Bushveld, Vaalbank) displaying only the ancient southern African ancestry component (Fig. 2c (pink)); (2) eight individuals with a majority of the ancient southern African ancestry component (pink), and with some level of an eastern African (purple and green) or European (green) ancestry; and (3) six individuals who display a substantial western African ancestry component (yellow), with minor fractions from the ancient southern African ancestry (pink), eastern Africa (purple) and sometimes with a European ancestry component (Plovers Lake 1, Tobias Cave 1).

Our findings therefore contrast with linguistic, archaeological and some early genetic studies pointing to a shared ancestry or long-term interaction between eastern, western and southern Africa. For example, it was suggested that the contemporary southern African Khoe-San people are the descendants of a once-widespread population that extended across much of southern, eastern and northeastern Africa^31^. Instead, there was deep population stratification (Figs. 2 and 3), with gene flow reaching south of 15° S only by 1.3 ka. The proposed linguistic connection between eastern African Hadza people and southern African click languages has been refuted, and the connection between eastern African Sandawe people and southern African click languages is now seen as resulting from the introduction of pastoralism to southern Africa after 2 ka (ref. ^32^).

## Partial continuity into Khoe-San

Modern-day Khoe-San groups show ample recent admixture with western African, eastern African and non-African groups. In 25 Khoe-San genomes selected for ‘least admixture’^7^, we estimate an average ancient southern African ancestry of around 79%. The last unadmixed individuals (in our sequenced data) seemingly disappeared a few centuries ago (Fig. 2c). Modern-day Juǀ’hoansi (around 11% eastern African admixture) and Karretjie People (around 17% mainly western and eastern African admixture) show the greatest genetic similarity to the ancient southern Africans (Fig. 2c, Extended Data Fig. 2 and Supplementary Fig. 6). However, the ancient southern Africans were substantially genetically differentiated from Juǀ’hoansi individuals (Wright’s fixation index* F*_ST_ = 0.055; similar to modern-day Finnish versus Chinese individuals; Supplementary Data 9). This differentiation is not solely due to admixture from eastern Africans—it also reflects the deep stratification of northern and southern San groups^4,7,27^ (Extended Data Fig. 2). Genetic differentiation was also distinct between ancient southern Africans and Karretjie People (*F*_ST_ = 0.041; similar to modern-day Indian versus Chinese individuals; Supplementary Data 9), consistent with partial continuity and recent admixture from western and eastern African groups.

## Long-term large population size

For the ancient African genomes (over tenfold coverage), as well as four pre-Neolithic Eurasians, three Neandertals and one Denisovan individual (Supplementary Information 2.5), pairwise genetic differences for the full genomes (*π*; Extended Data Fig. 5) showed around 1.41 differences per 1,000 bp (*π* = 1.41 × 10^−3^) between *Homo sapiens* and archaic humans (Fig. 2d). Pairwise differences between ancient southern Africans and other ancient Africans and pre-Neolithic Eurasians were slightly lower (*π* = 1.02 × 10^−3^) than between Neandertals and Denisovans (*π* = 1.14 × 10^−3^). Pairwise differences within the group of ancient southern Africans (*π* = 0.82 × 10^−3^) was slightly greater than between ancient western and ancient eastern Africans (*π* = 0.79 × 10^−3^). Heterozygosity (*H*_O_) for ancient southern Africans (mean across genomes; *H*_O_ = 0.80 × 10^−3^) was similar to other ancient Africans, only surpassed by an ancient western African individual (*H*_O_ = 0.93 × 10^−3^), indicating a large Holocene population size in southern Africa (Fig. 2e). A multiple sequentially coalescent approach (Supplementary Information 2.11) shows that the effective population size (*N*_e_) was large for several hundred thousand years, up to *N*_e_ ≈ 30,000 around 200 ka (Supplementary Fig. 10), similar to other African groups^7^. The large *N*_e_ at ≥300 ka for all humans was potentially caused by population subdivision^29^. We note a decline in *N*_e_ for ancient southern Africans from around 100–50 ka, to *N*_e_ ≈ 10,000 by the Last Glacial Maximum (20 ka), similar to non-African groups and the ancient northern Africans^22^.

**Extended Data Fig. 4:**
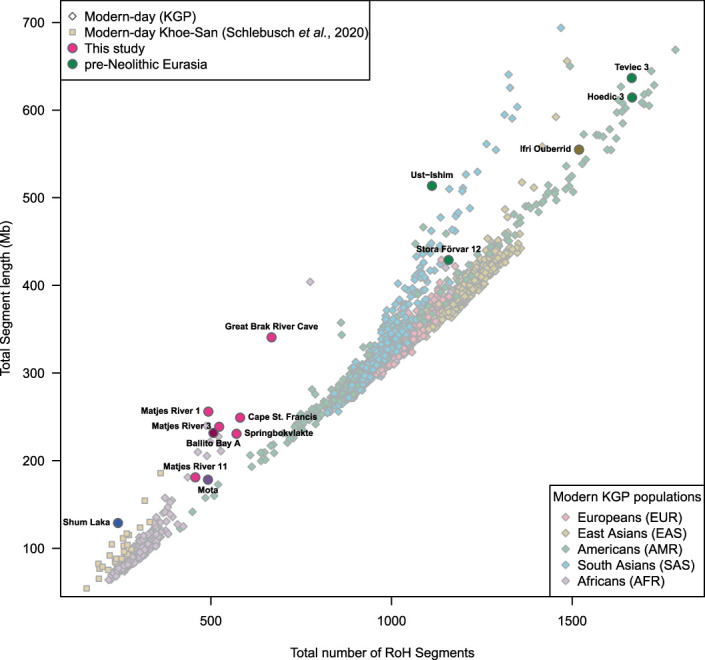
Patterns of runs of homozygosity (*RoH*) among studied populations. For each individual, the total segment length of *RoH* is given on the y-axis (in Mb), and the total number of *RoH* segments is given on the x-axis.

**Extended Data Fig. 5:**
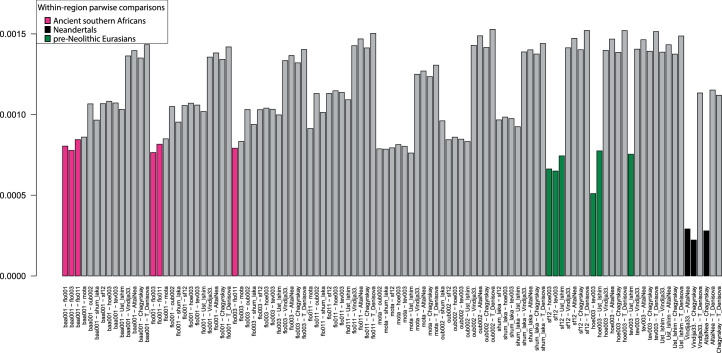
Pairwise differences for 15 complete ancient genomes. Some clear trends were visible: (i) 1.4 × 10^−3^ differences between archaic humans (Neandertals and Denisovans) and ancient humans (here represented by ancient Africans and pre-Neolithic Eurasians); (ii) 1.14 × 10^−3^ differences between Neandertals and Denisovans, (ii) 1.02 × 10^−3^ differences between ancient southern Africans and ancient northern, eastern, western Africans and pre-Neolithic Eurasians; (iii) 1.0 × 10^−3^ to 0.85 × 10^−3^ differences for a range of between-region pairs, and (iv) 0.8 × 10^−3^ to 0.7 × 10^−3^ differences for within-region comparisons.

**Extended Data Fig. 6:**
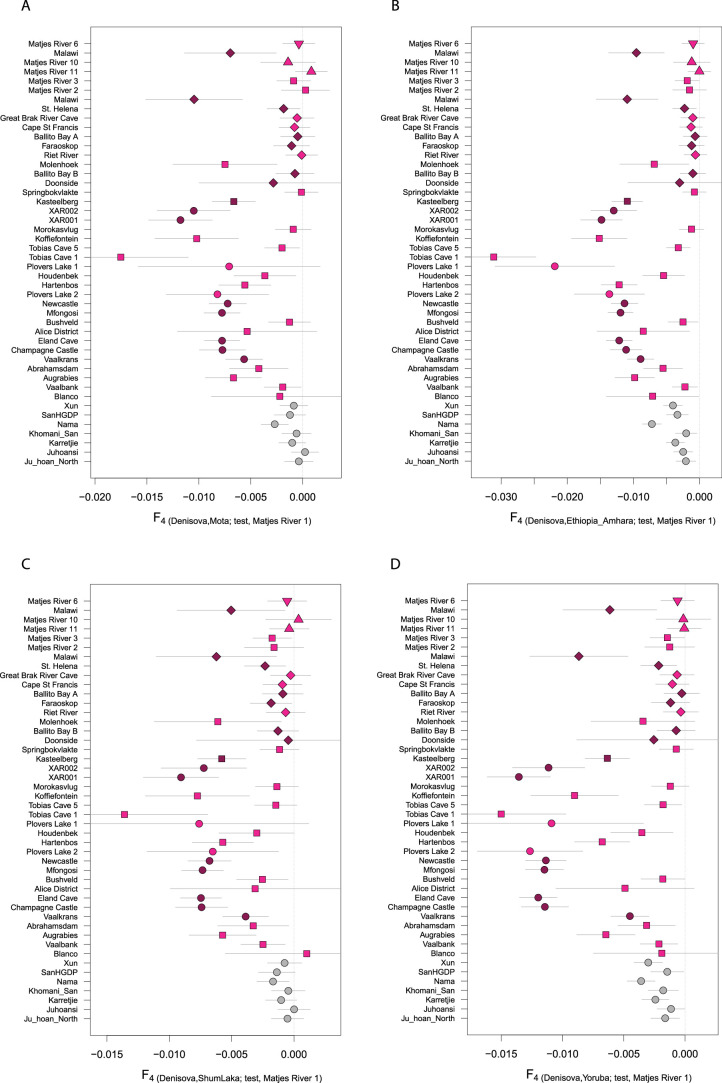
Test for gene-flow (*f*_4_) from eastern or western Africa into ancient southern Africa. A) *f*_4_ test for gene-flow from ancient eastern Africa (Mota), B) *f*_4_ test for gene-flow from eastern Africa (Ahmara), C) *f*_4_ test for gene-flow from ancient western Africa (Shum Laka), D) *f*_4_ test for gene-flow from western Africa (Yoruba) into ancient southern Africa. Standard errors were estimated using block jackknife and 5 Mb genomic blocks weighted by the number of loci. Error bars indicate 95% confidence interval (CI).

Runs of homozygosity (ROH, where greater numbers and total length of ROH segments indicate a smaller population size; Extended Data Fig. 4) show that the ancient southern Africans were at the upper tail of the distribution of modern-day Africans, but less extreme than most non-Africans—a pattern attributed to the out-of-Africa bottleneck. This indicates a smaller population size (relative to, for example, western African groups) in the relatively recent history of each individual, but still larger compared with non-Africans and ancient northern Africans (Extended Data Fig. 4). Most ancient southern Africans are shifted towards greater total segment ROH length without affecting the total number of ROH segments (shifted off the diagonal line in Extended Data Fig. 4), in particular the Great Brak River (2,355–2,310 cal. bp) and the Matjes River 1 (7,845–7,690 cal. bp) individuals. This pattern indicates a smaller recent ancestral population size, possibly with elements of inbreeding, indicating isolation and fragmentation among ancient southern Africans during the Holocene (see Supplementary Information 4.1 and Supplementary Fig. 4 for diet variability). Ancient southern Africans south of the Limpopo River therefore consisted of a large, stable population for many millennia, with a modest decline since around 50 ka, and a possible fragmentation and further decline during the Holocene.

## Southern Africa as a long-term refugium

Cultural contact between southern African foragers and incoming farmers is detected archaeologically from around 2 ka (refs. ^24,33^). The palaeoanthropological record shows a similar transition pattern in the late Holocene, but also a distinct difference in fossils (for example, the Hofmeyr cranium) compared to before the Last Glacial Maximum^34^ (Supplementary Information 4.1). Genomic data are compatible with southern Africa serving as a geographical refugium to a large human population for several hundred thousand years, affected only by gene inflow in the last millennium^4,19^ (Fig. 2). However, gene outflow from southernmost Africa probably happened in pulses during favourable climatic conditions^35^, potentially already around 70 ka (ref. ^30^) (Fig. 3). This resembles an ‘isolation by fragmentation’ model in which human groups were separated in different African refugia for extended periods^35^, as opposed to an ‘isolation by distance’ model with maintained gene flow across the continent. It appears that during favourable conditions, when the isolation-by-fragmentation ceases, gene flow is one-directional into intermediate areas that are depopulated for extended periods of less favourable conditions.

Alternatively, the southern African group inhabited the region up to 11–12° S before the Holocene, followed by an expansion of eastern African groups during Holocene, first to south-eastern Africa (as observed in the 8 ka mixed-ancestry genomes of Malawi)^19^ and reaching southernmost Africa in the last millennia. Either way, the genomic data of ancient southern Africans with its distinctiveness and lack of gene inflow before around 1.3 ka suggest that southern Africa represents a long-standing human refugium, with potential gene-outflow pulses. Although direct comparisons of complete genomes do not rule out complex demographic histories further back in time, nor low levels of archaic admixture, the deep human history in Africa can be represented by deep stratification—including long-term isolation—between southernmost Africa on the one hand and western, central and eastern Africa and the rest of the world on the other (Fig. 3).

## *Homo sapiens*-specific genetic variants

Early *Homo sapiens* fossils with archaic and modern features dating to around 300–190 ka were found in southern^36,37^, eastern^38,39^ and northern Africa^2^. Some of the oldest *Homo sapiens* fossils with modern features come from Border Cave, South Africa, at around 171–152 ka (ref. ^40^), and Omo Kibish in Ethiopia at around 230 ka (ref. ^41^). Thereafter, all human remains in southern Africa are modern^24^, with some of the earliest archaeological evidence of modern human behaviour/thinking from at least 100 ka (ref. ^42^).

As behavioural and cognitive traits are largely heritable, southern Africa’s deep *Homo sapiens* genomic record could aid in disentangling the ‘sapient paradox’, whereby anatomical modernity purportedly predates modern behaviour^43^. However, this may not be straightforward, because some genes governing cognition and anatomy evolved rapidly in the early human lineage^12^, and genetic variants governing rapid neuron developments thought to be fixed in humans^44^ are highly variable in some populations^45^. Yet, our understanding of human cognitive evolution increases with cataloguing genetic variants associated with cognitive traits^46^, and will benefit from improved understanding of genetic trait architecture^47^, coupled with more ancient human genomic data.

Although understanding the genetic architecture of traits surely involves variants in regulatory elements^47^, amino acid-altering variants result in bona fide differences at the protein level, with a potential for trait differences. A previous study^45^ found that 24 out of 113 amino acid-altering variants that were thought to be fixed for the derived state among modern humans (and fixed for the ancestral state in a Neandertal genome) were in fact variable among 25 modern-day Khoe-San individuals^7^. Of these, 13 variants are also variable among the smaller set of 7 ancient southern African complete genomes (Fig. 1a and Supplementary Data 3). One of these derived variants in the *TKTL1* gene on the X chromosome is linked to increased neocortical neurogenesis in the frontal lobe^44^. The derived variant has been reported to be almost fixed (99.97%) in modern humans in contrast to Neandertals and Denisovans who carry the ancestral variant^44^, but the ancestral variant is common among modern-day Khoe-San (32%)^7,45^, and among the 7 ancient southern African complete genomes (27%). Thus, superficially, the derived variant of *TKTL1* appears to be almost fixed in modern humans. Yet, its ancestral variant is common in some populations that are under-represented in genomic investigations. The derived variant therefore represents a false positive that is unlikely to be important for the development of complex modern human neurological characteristics.

To assess genetic variants that are unique to *Homo sapiens* (variants that arose somewhere on the branch leading to *Homo sapiens* or on an internal branch among humans; shown as thick black lines in Fig. 2d), we extracted all variable sites across the complete genomes (over 7.2× coverage) of the 7 ancient southern Africans, 3 ancient Africans from the east (*n* = 1), west (*n* = 1) and north (*n* = 1), 7 pre-Neolithic Eurasians, 7 Northern San (Juǀ’hoansi), 5 Southern San (Karretjie People), 208 individuals (8 individuals each from 26 populations) from the 1000 Genomes Project (1KGP) and 4 archaic humans (3 Neandertals and 1 Denisovan). We coin the term *Homo sapiens*-specific variants for derived variants in the set of *Homo sapiens* where the 4 archaic humans were fixed for the ancestral variant.

**Fig. 4:**
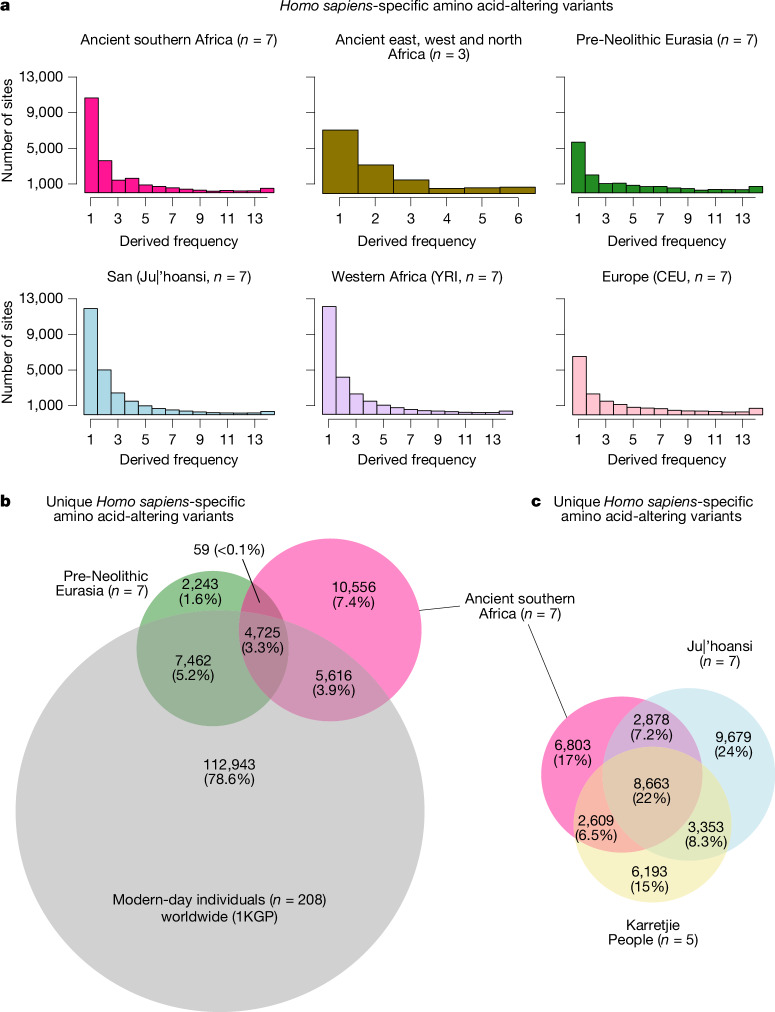
*Homo sapiens*-specific amino acid-altering variants in the human genome. **a**, Frequency spectra of *Homo* *sapiens*-specific variants that alter amino acid code, shown for six groups, and a sample of seven individuals (except for ancient east, west and north Africans). YRI, Yoruba in Ibadan, Nigeria; CEU, Utah residents with northern and western European ancestry. **b**, Venn diagram of 143,604 *Homo* *sapiens*-specific amino acid-altering variants in seven ancient southern Africans and seven pre-Neolithic Eurasians compared with 208 individuals from the 1KGP (Supplementary Information 2.5 and Supplementary Data 7). **c**, Venn diagram of 40,178 *Homo* *sapiens*-specific amino acid-altering variants in seven ancient southern Africans, seven Northern San individuals (Juǀ’hoansi) and five Southern San individuals (Karretjie People; Supplementary Data 7).

**Extended Data Fig. 7:**
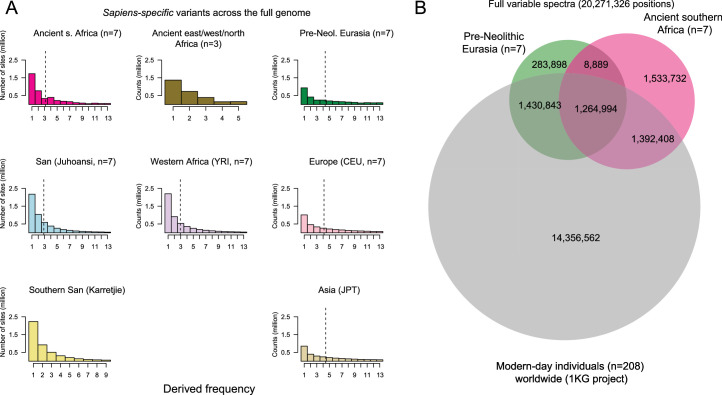
*Sapiens*-specific genetic variants in 8 groups. A) Frequency spectra of *sapiens*-specific variants in the full human genome for 8 groups. The mean frequency (out of 2 × 7 = 14 gene-copies) is shown as a dashed vertical line for groups with 7 individuals. B) Venn-diagram of 20,271,326 *sapiens*-specific variants in 7 ancient southern Africans and 7 pre-Neolithic Eurasians compared to 208 individuals from the 1KG project (compare with Fig. 4, see also Supplementary Data 7 for details).

Among amino acid-altering *Homo sapiens*-specific variants fixed in the 208 1KGP individuals (in total, 106 sites), 12.3% were variable among the ancient southern Africans, 10.4% among the northern San, 12.3% among the southern San and 5.7% among the pre-Neolithic Eurasians. Thus, a substantial fraction of seemingly fixed variants was not fixed when studying a more diverse set of humans. More generally, the genomes of ancient and modern-day groups revealed many low-frequency amino acid-altering *Homo sapiens*-specific variants, as well as several modest-to-high-frequency variants (Fig. 4a). African groups showed a shift towards the low-frequency variants, whereas the non-African groups displayed a flatter frequency spectrum. The *Homo sapiens*-specific variants across the entire genome showed similar patterns, with the ancient southern Africans displaying a spectrum in-between the modern-day African groups and the non-African groups (Extended Data Fig. 7a and Supplementary Data 4).

There were 490 *Homo sapiens*-specific amino acid-altering variants (distributed across 420 genes) fixed among the ancient southern Africans (Supplementary Data 10), compared with 358 for Juǀ’hoansi and 364 for Yoruba individuals. Non-African groups (including pre-Neolithic Eurasians) displayed greater numbers of such fixed variants (around 700–800 per group), consistent with lower diversity caused by the out-of-Africa migration bottleneck. Among the 490 *Homo sapiens*-specific variants fixed in ancient southern Africans, immune-system-related genes showed the greatest enrichment (Supplementary Data 11). We note also that most genes have multiple functions: almost half (44%) of the *Homo sapiens*-specific amino acid-altering variants fixed in ancient southern Africans were also listed as genes with non-synonymous changes at high frequency in modern humans associated with the cellular features of neurons, probably resulting in brain growth trajectories and complex cognitive trait phenotypes^46^.

By focusing on *Homo sapiens*-specific amino acid-altering variants fixed among all investigated individuals, including the ancient southern Africans, we found a considerable enrichment and over-representation of kidney function (7 out of 79 genes and 14 out of the top 15 Gene Ontology categories showed direct links to kidney function; Supplementary Data 12 and Supplementary Information 3.12.1). Some of these genes were previously found to differ between archaic humans and modern humans^48^. This demonstrates that many *Homo sapiens*-specific amino acid-altering variants that became fixed among modern humans were affecting kidney function, whereas Neandertals and Denisovans retained the ancestral variants, suggesting rapid adaption of kidney function on the *Homo sapiens* lineage, potentially connected to improved water retention. This trait is specific to humans compared with other great apes^49^.

By focusing on *Homo sapiens*-specific amino acid-altering variants fixed among the ancient southern Africans that display low frequencies in other human groups, we can isolate local adaptations (Supplementary Data 13 and 14). Among the top 10 variants that showed the greatest difference between ancient southern Africans and the 1KGP individuals, 3 variants were located in genes associated with ultraviolet-light protection, skin diseases and/or skin pigmentation^50^ (Supplementary Data 14 and Supplementary Information 3.12.2). We speculate that ultraviolet-light protection was an important southern African adaptation due to the region’s arid, grassland/savanna ecologies offering limited natural protection^12^.

**Extended Data Fig. 8:**
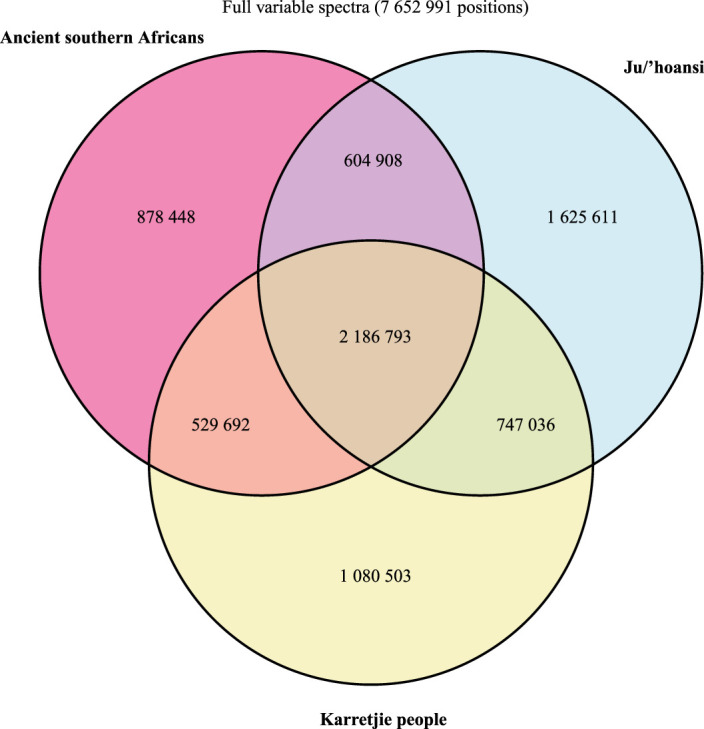
*Sapiens*-specific genetic variants among ancient and modern-day Indigenous southern Africans. Venn-diagram presenting the distribution of 7,652,991 *sapiens*-specific variants in 7 ancient southern Africans, 7 Northern San (Juǀ’hoansi) and 5 Southern San (Karretjie people, see Supplementary Data 7).

A marked 50.4% (10,556) of the *Homo sapiens*-specific amino acid-altering variants were unique to the ancient southern Africans (20,956 such sites were variable among them), compared with a representative set of 208 individuals from the 1KGP (Fig. 4b). By contrast, only 15.5% (2,243 out of 14,489) of *Homo sapiens*-specific amino acid-altering variants were unique among pre-Neolithic Eurasians compared with the same set (Fig. 4b). Replacing the ancient southern Africans with northern San (Supplementary Fig. 14) or southern San (Supplementary Fig. 15) individuals gives similar numbers, but the sets of variants from the modern-day San groups and the ancient southern Africans only overlap partly (Fig. 4c and Extended Data Fig. 8), demonstrating that these three groups carry large amounts of private *Homo sapiens*-specific amino acid-altering variants.

Cumulatively, the genomes of the ancient southern Africans show that this group displays many *Homo sapiens*-specific variants (and variable positions) at amino acid-altering sites, also reflected among the modern-day San people. This observation cannot be explained solely by a large, stable southern African population, which retained derived variants to a greater extent compared with other groups. The ancient southern Africans were probably also isolated from other African groups for long periods. The derived variants unique to southern Africans may also signal low-to-modest gene flow from an unknown/unsampled group of genetically differentiated humans.

Irrespective of cause, the many variable amino acid-altering sites among the ancient southern Africans point towards a genetic model in which different protein variants can be combined to viable outcomes. The assumption that certain variants (detected in modern-day mostly urban populations) are important for the architecture of some *Homo sapiens* traits requires greater scrutiny. We therefore suggest that the vast genetic variation still unassessed in ancient genomes from Indigenous peoples globally is important for advancing our understanding of the evolution of *Homo sapiens*. The many *Homo sapiens*-specific variants found in southern African genomes point to a combinatorial genetic model of human evolution^45^ in which there are many possible combinations of genetic variants that lead to ‘genetically modern’ *Homo sapiens*. It may also be that regulatory variants (and not amino acid-altering changes) were the primary building blocks during the latest steps in human evolution. How this process came about and which (combinations of) variants were important may be deciphered during the next decade by unravelling genetic trait architecture^47^. Importantly, the complete genomes of the ancient southern Africans reveal distinct patterns of a large number of derived genetic variants altering the amino acid sequence of proteins, and probably also their function. The ancient southern African genomes contained many such variants that were not found in any other group (ancient or modern day). These observations are important for advancing our understanding of genomic variation in humans, and combinations of genetic variants that are key to *Homo sapiens* evolution.

## Methods

Detailed methods descriptions for each section are provided in the Supplementary Information.

### Archaeological sampling

The majority (22 out of 28) of ancient human remains analysed in this study were housed at the National Museum of Bloemfontein at Florisbad Quaternary Research Station, Free State, South Africa, whereas the remaining human remains were housed at the School of Anatomical Sciences at Wits Medical School University of Witwatersrand, Gauteng, South Africa. Sampling of teeth and bone elements was done on site in a mobile clean-laboratory and the sampled elements were immediately returned. The samples were transported to the Ancient DNA Laboratory at Uppsala University, Sweden, for further analyses.

### Radiocarbon dating

In total, 20 human remains were sampled for accelerator mass spectrometry (AMS) radiocarbon dating and sent to Beta Analytic where bone collagen extraction were performed for AMS radiocarbon dating and for stable dietary isotope analyses using isotope-ratio mass spectrometry. Conventional radiocarbon dates obtained from Beta Analytic were modelled using BetaCal 3.21 and SHCal13 (ref. ^51^), whereas radiocarbon ages for three previously dated individuals were modelled using OxCal v.4.4 and SHCal20 calibration curves^52,53^.

### Ancient DNA retrieval

DNA was extracted either as in ref. ^54^ with modifications as described in ref. ^55^ or ref. ^56^. DNA extracts were prepared from the 28 human remains (between 1–7 DNA extracts from each individual). One blunt-end library was prepared for each DNA extract and sequenced for screening of endogenous human DNA content and verification of ancient DNA using deamination profiles^57,58^. Authentic ancient DNA (and >1% human DNA) was found in 32% of the sequencing libraries that were used for screening. For DNA extracts for which the proportion of ancient human DNA was >2%, sequencing libraries were prepared using UDG treatment to minimize post-mortem deaminations and increase the sequencing depth^59^. For DNA libraries for which the proportion of human ancient DNA was <2%, additional blunt-end libraries were prepared to increase the sequencing depth. Owing to low amounts of endogenous human DNA, some of the libraries were enriched using MY-bait African Human Whole Genome Capture Kit (MYcroarray) according to the manufacturer’s instructions (MYbaits manual v.2.3.1) and amplified as described previously^4^. All libraries were sequenced on either the HiSeq X10 or a NovaSeq 6000 (SP flow cell) Illumina sequencer with either 100 bp or 150 bp paired-end chemistry.

### Data processing and authentication

Adapters and low-quality bases were trimmed from the sequencing data and paired-end reads were merged if an overlap of at least 11 bp was detected between the forward and reverse read, using either the script MergeReadsFastQcc.py^60^ or AdapterRemoval (v.2.1.7)^61^. The reads were then mapped against the human reference genome build 37 (hs37d5) using bwa aln^62,63^. BAM files from resequenced libraries were merged using Samtools merge (v.0.1.19)^64^ before PCR duplicates were identified and collapsed using a slightly modified version of FilterUniqeSAMCons.py^60^. Non-UDG and UDG-treated libraries were then separately merged per individual and reads shorter than 35 bp and with <90% consensus with the reference sequence were filtered out using percidentity_threshold.py^65^. Mitochondrial contamination was estimated using two different methods—Green and contamMix^66,67^. Additional contamination estimates were performed on all individuals with genome-wide coverage >2× using VerifyBamID^68^. Contamination estimates were generally low for both the nuclear and mitochondrial genome (Supplementary Data 1 and 5).

### Determining sex, uniparental haplogroups

Genetic sex was determined using the X/Y coverage ratio^69^. Mitochondrial haplogroups were inferred for all individuals with Haplogrep and Phylotree Build 17 (refs. ^70,71^). Y-chromosome haplogroups were assigned by using Samtools (v.1.3)^64^ mpileup to call single base substitutions from Phylotree^72^ from BAM files mapped to the hs37d5 (hg19) reference genome. Sites with a mapping quality and base quality of at least 30 were extracted. Indels, transitions and A/T and C/G SNPs were excluded to avoid potential mix-up with deamination damage and strand misidentification.

### Analysis of pseudohaploidized data

To investigate population stratification and genetic affinities among individuals, we compiled a genome-wide dataset of all ancient southern Africans in this study (Extended Data Table 1) merged to comparative modern-day individuals (Supplementary Data 8) and published ancient African individuals (Supplementary Data 7). Variants from the Human Genome Diversity Project^73^ lifted to the hg19 reference genome were selected as known variants to call genotypes from the ancient DNA data (Extended Data Table 1 and Supplementary Data 7). For individuals with only non-UDG-treated sequencing data, all transition sites were coded as missing data to avoid effects of post-mortem damage. For those individuals for whom we had both UDG-treated and non-UDG-treated libraries, a read from either of the two libraries was randomly sampled for transversion sites, and only reads from UDG-treated libraries were sampled from transition sites. At each SNP site, a random read with a minimum mapping and base quality of 30 was drawn and the allelic status at that read was coded to be the hemizygous genotype of the individual. Sites showing more than two alleles or indels were removed from the data. Published ancient African sequencing BAM files were downloaded and processed using the same pipeline as described above, while comparative modern-day whole-genome sequencing data were processed as follows: all-site VCF files were downloaded where available. The data were lifted from hg38 to hg19 if applicable, and positions that switched chromosomes or ended up as duplications were removed from further processing. The data were filtered for 10% missingness and Hardy–Weinberg equilibrium with a *P* value of 0.000001. The all-site VCF files from ref. ^7^ were additionally filtered for quality and 10% missingness before processing. The final genome-wide dataset was filtered using PLINK v.1.9 (www.cog-genomics.org/plink/1.9) for a minimum allele frequency of 10% and linkage-disequilibrium pruned using command --indep-pairwise 50 5 0.4. All modern-day individuals were further pseudohaplodized before analyses.

Analyses of population stratification were conducted using both PCoA with PLINK --pca and principal component analyses (PCA) using smartpca from the Eigensoft package^74,75^. The PCAs were performed both as unprojected and projected (with parameter lsqproject: YES) with the non-default parameters r2thresh: 0.7 and shrinkmode: YES. The program ADMIXTURE (v.1.3.0)^76^ was used for unsupervised estimation of ancestry components. A total of 25 iterations was run for each value of assumed number of clusters (*K*), with *K* ranging from 2 to 10. The program popstats^77^ was used to estimate *f*_4_ statistics and *f*_3_ statistics (using the -f3vanilla option). To minimize bias introduced by ascertainment when performing *f*-statistics, only sites that are polymorphic between the Altai Neandertal and Denisovan were investigated^19^ (approximately 500,000 sites).

Population continuity among ancient southern Africans was investigated using the approach developed previously^78^. This method conditions on heterozygous sites in an ‘anchor’ individual (the oldest high-coverage individual, Matjes River 1) and counts the proportion of derived alleles occurring at those sites in more recent individuals. This statistic, forward in time from the anchor population/individual, is unaffected by genetic drift, but decreases with gene flow from a genetically differentiated population.

### Analysis of complete genomes

Diploid genotype calling on a per chromosome basis were performed using snpAD^79^. The raw VCF files were filtered so that only regions passing the following criteria were retained: unique mapability of 35 bp, positions had a covering depth (DP)of ≥4 and a quality of ≥30. All of the sample VCF files, on a per-chromosome basis, were then merged and annotated using dbSNP v.142 using bcftools annotate^80^. The command LiftoverVCF implemented in picard v.3.1.1 and the chain file ‘hg19ToHg38.over.chain.gz’ from UCSC were used to create a version of the dataset in the GRCh38 reference coordinate system. Positions that switched chromosome were removed before downstream analyses. For diploid analyses, we used two versions of the phase 3 data from the 1000 Genome project as comparative data. A filtered version of the dataset in hg19 coordinates was downloaded from https://hgdownload.soe.ucsc.edu/gbdb/hg19/1000Genomes/phase3/, whereas the CRAM files, in GRCh38 coordinates, for eight randomly collected individuals per population were downloaded from the European Nucleotide Archive. Diploid genotype calling was then performed as described previously^7^. To retrieve the full spectra of genetic variation in present-day southern African Khoe-San individuals, five populations from ref. ^7^ were also used as comparative data.

ROH values were calculated using PLINK (v.1.9) and MSMC (v.0.1.0)^81^ was run per individual to estimate the effective population size as a function of time (assuming a mutation rate of 1.45 × 10^−8^ per bp per generation, and a generation time of 29 years).

Summary statistics were estimated using Bcftools stats^80^. Per-individual heterozygosity was estimated as the number of heterozygous genotype calls over the total number of genotype calls. Per-chromosome genetic distance matrices were estimated using the software VCF2Dis^82^. Chromosome length differences were handled by weighting each chromosome by its contribution to the autosomal genome (chromosome length/total autosomal length).

Pairwise *F*_ST_ was computed using VCFTools (v.0.1.16)^83^ using the --weir-fst-pop parameter (set twice, one per each population in the comparison), which estimates Weir and Cockerham’s^84^ fixation index on a per-site basis.

Estimates of population divergence time among individuals were obtained using the two–two–outgroup site-frequency-based methods^85^ using Denisovan and Neandertal as outgroups. A weighted block jackknife procedure with 5 Mb blocks was used to estimate the confidence intervals of estimates. To rescale the estimated divergence times from generations to chronological years, a mutation rate of 1.45 × 10^−8^ (per bp per generation) and a generation time of 29 years was used. VCF files were filtered to pass only biallelic sites with a QUAL > 30 and a reference or alternative allele matching the ancestral state present in three great apes. For a site to be considered informative, it must pass allele-depth thresholds set by the lower and upper 5% of site coverage distributions, and a minimum allele depth of 4 sequencing reads.

Biallelic SNPs (lifted to GRCh38) were annotated with SnpEff^86^ for functional effect using the hg38kg genome supported by the program. For each set of populations, allele frequencies were estimated and the ancestral state was determined for all variable sites by assessing the genomes of three great apes (chimpanzee, gorilla and orangutan). Only sites with data from at least one great ape and consensus among the great apes were analysed further. We further restricted our analyses of both the full-genome spectra as well as the amino acid-altering sites to where the four archaic genomes were fixed. For each group, we plotted the SFS for derived variants that were fixed for the ancestral variant among four archaic genomes. Gene Ontology term enrichment analyses were performed by linking amino acid-altering variants to genes and then query the gene list using the WEB-based Gene SeT Analysis Toolkit (https://www.webgestalt.org), using the ‘Over-Representation model’ for *Homo sapiens*. As a functional database, we used Gene Ontology for Biological Processes using the reference set ‘genome’. We used the default parameter settings except for changing ‘significance level’ from 10 to 15.

### Ethics and inclusion statement

The sampling for this study was authorized by the various South African heritage Resources Agencies (Supplementary Information 2.1) and emerged from collaborations that involved local universities and researchers—including J.B., B.Z. and M.L—whose involvement in the research design included the selection of archaeological material for analyses as well as sampling supervision. The local relevance of this research is tied to the region’s history and it is locally relevant for describing the human past in southern Africa. The study was undertaken to the highest standards of palaeogenomic research and relevant research by local scholars was cited.

### Reporting summary

Further information on research design is available in the Nature Portfolio Reporting Summary linked to this article.

## Online content

Any methods, additional references, Nature Portfolio reporting summaries, source data, extended data, supplementary information, acknowledgements, peer review information; details of author contributions and competing interests; and statements of data and code availability are available at 10.1038/s41586-025-09811-4.

## Supplementary information

Supplementary Information Supplementary Background, Methods, Results, Discussion and References, including Supplementary Tables 1–5 and Supplementary Figs 1–15, and details of Supplementary Data 1–31.

Reporting Summary

Supplementary Data Supplementary Data 1–31.

## Figures and Tables

**Extended Data Table 1:**
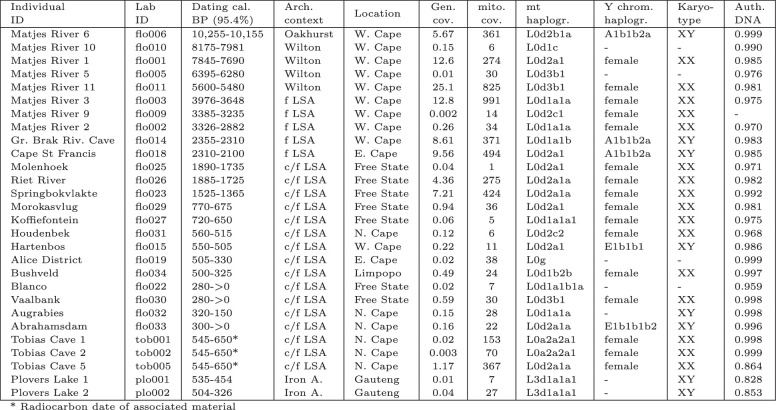
The 28 individuals whose genomes were sequenced in this study

Key: mito = mitochondrial, cal = calibrated, BP = before present (present defined as 1950). Proportion of authentic DNA estimated from the mitochondria (SI section 2.3, see Supplementary Data 1 & 5 for additional quality statistics).

## Data Availability

The called variants for the ancient individuals in this study are available at Zenodo^87^ (10.5281/zenodo.17295109) and the sequencing data for the complete genomes are available at the European Nucleotide Archive under the accession number PRJEB98562.
