# Supplementary material for: Homo sapiens-specific evolution unveiled by ancient southern African genomes
Source: Nature. 2025 Dec 3;650(8100):156–63. doi: 10.1038/s41586-025-09811-4 (PMC12872451; doi:10.1038/s41586-025-09811-4)
Supplement: Supplementary file 1 — Supplementary Background, Methods, Results, Discussion and References, including Supplementary Tables 1–5 and Supplementary Figs 1–15, and details of Supplementary Data 1–31. [file 41586_2025_9811_MOESM1_ESM.pdf]

nature portfolio

https://doi.org/10.1038/s41586-025-09811-4

Supplementary information

Homo sapiens-specific evolution unveiled by ancient southern African genomes

In the format provided by the authors and unedited

Contents

1 Supplementary background 3
1.1 The individuals in the southern African archaeological sequence 3
1.2 The Matjes River individuals in archaeological context 8
1.3 Relationship of ancient southern Africans and modern-day indigenous Khoe-San groups 9

2 Supplementary material & methods 10
2.1 Sampling and permits 10
2.2 DNA and isotope data collection 11
2.3 Sequencing and data processing 12
2.4 Inference of uniparental haplogroups 14
2.5 Comparative genomic data 15
2.6 Filtering genome-wide data 16
2.7 Assessing stratification with MDS/PCoA/PCA 16
2.8 Estimating ancestry components 18
2.9 Tests of genetic affinity with f-statistics 19
2.10 Correlations between genetic and geographic distance, and time 20
2.11 Assessing effective population size over time with MSMC 20
2.12 Runs of Homozygosity 21
2.13 Genetic diversity within and between complete ancient southern African genomes 21
2.14 Estimating genetic differentiation with F_{ST} 22
2.15 Examining the frequency spectra of full genomes and amino-acid altering sites 22
2.16 Assessing population continuity 24
2.17 Estimating population divergence times 24

3 Supplementary results 26
3.1 Dietary analysis and radiocarbon dating 26
3.2 Mitochondrial haplogroups 28
3.3 Y-chromosome haplogroups 28
3.4 Variants associated with traits 29
3.5 How far back in time does the ancient southern African ancestry extend? 29
3.6 Recent gene-flow from eastern and western African groups 32
3.7 Population continuity at Matjes River 34

3.8 Kinship and stratification among ancient southern Africans . . 35
3.9 Modern-day Khoe-San groups carry the greatest level of ancient southern African ancestry 38
3.10 Past population sizes of ancient southern Africans 40
3.11 Population divergence times 41
3.12 *Sapiens*-specific variants 47
3.12.1 *Sapiens*-specific variants and kidney function 50
3.12.2 *Sapiens*-specific variants enriched among the ancient southern Africans 52

4 Supplementary discussion 53
4.1 Palaeoanthropological context 53
4.2 On population stratification and large diversity 54

1 Supplementary background

1.1 The individuals in the southern African archaeological sequence

Southern Africa has an archaeological record that spans the last 2 million years. All individuals included in this study date to the Holocene epoch, that is, the last 12,000 years, which contains archaeological phases of the Stone Age (Lombard *et al.*, 2022, 2013) and Iron Age (Huffman, 2007, 2009) (SI Table 1).

In the Stone Age context, the older three phases, i.e., Oakhurst, Wilton, and the final Later Stone Age, all represent *bona fide* hunter-gatherer populations and subsistence patterns. The Oakhurst phase, which is the oldest Holocene Stone Age technocomplex, starting at about 12,000 years ago, is defined by a largely macrolithic, flake-based industry characterized by round, end, and D-shaped scrapers and adzes with few or no microliths. During this phase, a wide range of polished bone tools also occur (Deacon, 1984; Lombard *et al.*, 2013, 2022). The only individual with a certain Oakhurst date represented in this study is the Matjes River 6 boy (flo006) from Matjes River in the Western Cape (SI Fig. 1). Matjes River was excavated extensively, but poorly controlled, and interpretations of the archaeological material remain inadequately published in the form of theses (e.g., Louw, 1960; Ludwig, 2005). The radiocarbon dates for two other individuals from Matjes River; Matjes River 1 and Matjes River 10 (flo001, flo010), fall within the final stage of the Oakhurst that overlaps with the subsequent Wilton technocomplex starting at ∼8,000 years ago (SI Table 1, SI Fig. 1). Both Matjes River 6 boy (flo006) and Matjes River 1 (flo001) were found in layers associated with a macrolithic technology, previously thought to be of the Middle Stone Age, but the assemblage is too small and undiagnostic to be conclusive (e.g., Ludwig, 2005). Based on their radiocarbon ages and the macrolithic artefacts, the Later Stone Age Oakhurst phase is the best fit for these individuals.

The Wilton phase generally lasts until about 4,000 years ago, but at some sites continues into the final Later Stone Age as regional variants. During this phase we see the introduction of a fully developed microlithic tradition with numerous formal tools, highly standardised backed microliths often made from blades/bladelets and small convex scrapers. Ochre and ostrich eggshell are common, and a range of bone, shell and wooden artefacts occur (Deacon,

Supplementary Information Table 1: Synthesised sequence for the archaeological phases of southern Africa (after Huffman, 2007, 2009; Lombard *et al.*, 2013, 2022; Loftus *et al.*, 2024). NOTE: The ceramic final Later Stone Age and the Iron Age pertains to South Africa (the current geopolitical country) only, because people associated with these phases arrived earlier in the northern parts of southern Africa (the region south of the Zambezi River).

HOLOCENE	
Iron Age sequence synthesized	
Phase	Broad duration (years ago)
Late Iron Age	~200-700
Middle Iron Age	~700-1,000
Early Iron Age	~1,000-1,800
Stone Age sequence synthesized	
ceramic final Later Stone Age	<2,000
final Later Stone Age	~1,000-4,000
Wilton	~4,000-8,000
Oakhurst	~7,000-12,000
PLEISTOCENE	
Robberg	~12,000-22,000
early Later Stone Age	~12,000-18,000
final Middle Stone Age	~18,000-40,000
post-Howiesons Poort	~20,000-40,000
Howiesons Poort	~45,000-58,000
Still Bay	~58,000-66,000
Mossel Bay	~70,000-77,000
early Middle Stone Age	~130,000-300,000
Fauresmith	~200,000-600,000
Acheulean (Stellenbosch)	~300,000-1,500,000
Oldowan	~1,500,000-2,000,000

1984; Lombard *et al.*, 2013, 2022). Individuals from the Wilton phase represented in this study include Matjes River 5 (flo005) and Matjes River 11 (flo011) woman. Matjes River 5 (who could not be karyotyped to determine the sex) was found on top of the macrolithic context at the site, and the Matjes River 11 (flo011) woman within the site's Wilton context (Morris, 1992a; Ludwig, 2005).

The final Later Stone Age starting at about 4,000 years ago shows much variability in its archaeological assemblages across southern Africa, but usu-

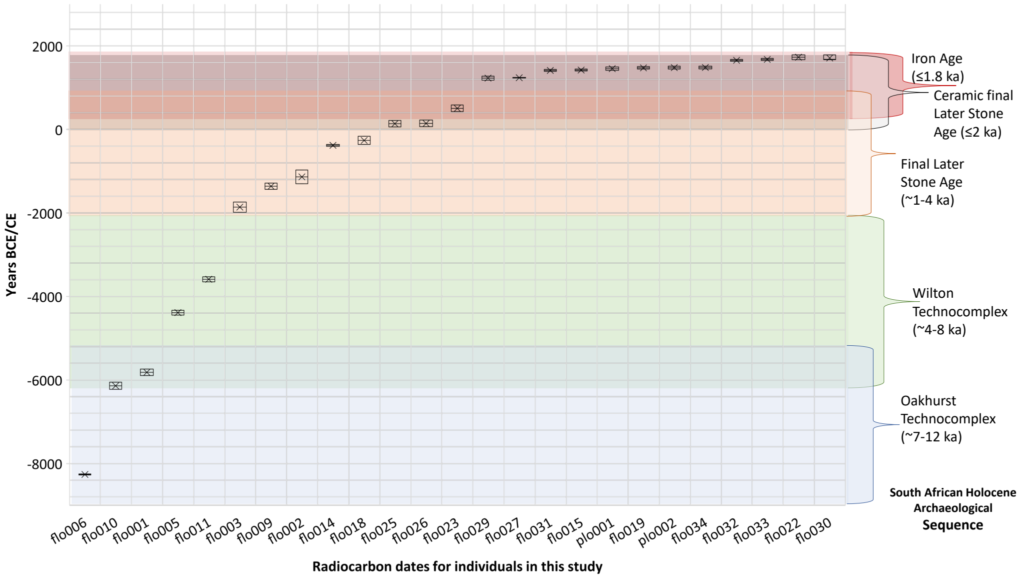

Supplementary Information Figure 1: Radiocarbon years (un-calibrated point estimate ± standard deviation) for the individuals in this study (Supplementary Data 2) plotted against the Holocene archaeological sequence of southern Africa.

ally include macrolithic assemblages that are mostly informal, often characterised by large, untrimmed flakes. Microlithic scrapers, blades and bladelets, backed tools and adzes may occur. Worked bone, ochre, and ostrich eggshell are common, and rare metal objects start to occur after about 2,000 years ago, but pottery is absent (Deacon, 1984; Lombard *et al.*, 2013, 2022). From Matjes River three individuals (Matjes River 3/flo003, Matjes River 9/flo009, Matjes River 2/flo002,) date to this phase, which is associated with a thick shellfish deposit (Hoffman, 1958), and Ludwig's (2005) description of the material culture of the layer is consistent with the final Later Stone Age. Other individuals included in this study with ages falling within the final Later Stone Age include two men, namely flo014 from Groot Brakrivier (Great Brak River), and flo018 from Cape St Francis (SI Fig. 1), both in the Western Cape. Today, Groot Brakrivier is a coastal village about 130 km west of the Matjes River site. The male individual (Great Brak River/flo014) and several other skeletons were excavated from shallow graves in a cave near the village in 1936 (Morris, 1992a). Morris (1992a) cites Dreyer (1933) as an author related to these remains, but the Dreyer publication pertains to Mat-

jes River only, and we could find archaeological context other than that this individual was found in a shallow grave (Morris, 1992a). For Cape St Francis (flo18) there is also no directly linked archaeological information (Morris, 1992a). Binneman (2004), however, reported on final Later Stone Age archaeology from Cape St Francis that dates between 5180±65 bp (Pta-1089) and 1770±50 bp (Pta-9311).

The dates of three women in our study (flo025, flo026, flo023) fall within the overlap between the final Later Stone Age and the ceramic final Later Stone Age (SI Fig. 1). Lithic assemblages of the ceramic final Later Stone Age are broadly similar to the final Later Stone Age, but now they are accompanied by grit- or grass-tempered pottery that can be coarse, or well-fired and thin-walled. The pottery may also have lugs, spouts and conical bases that differentiate it from local Iron Age pottery. Ochre and ostrich eggshell and ground-stone objects are common. Metal objects and glass beads also occur, mostly thought of as goods traded from Iron Age groups and later from Europeans (Deacon, 1984; Lombard *et al.*, 2013, 2022). This phase is generally associated with the hunting-foraging-dairying economy of the Khoekhoe people (Lombard & Parsons, 2015). All three women were found at sites in the Free State. The woman (flo025) in the group with the oldest radiocarbon date was found at Molenhoek in the Free State where she was excavated in 1961 from an 8-ft-deep fodder pit, but no further information is available (Morris, 1992a). Flo026 was excavated in 1962 from an isolated grave 55 m from the Riet River near Jacobsdal. She was buried at 1.2 m deep with flat stones on her body, some stone tools, beads and shells (Morris, 1992a). She is one of 83 skeletons associated with 79 Riet River graves (Morris, 1992b). These graves overlap geographically with the distribution of poorly preserved, circular stone-walled enclosures (Maggs, 1971). These settlements are thought to have been used by people who mainly followed a hunter-gatherer economy (Humphreys, 1972), perhaps supplemented with milk. They contain Later Stone Age artefacts with Khoekhoe pottery, and rare remains of domesticated animals. Although iron is sometimes found in these settlements, there is no evidence of iron smelting or working (Morris, 1992b), so that flo026 can probably be assigned to the ceramic final Later Stone Age. The cranium and mandible of the woman from Springbokvlakte (flo023) near Bethulie was found in 1953 in an aardvark hole, her radiocarbon age can be associated with the final Later Stone Age, the ceramic final Later Stone Age or the Iron Age. Because no archaeology was reported for

her (Morris, 1992a), it is impossible to position her more precisely in the sequence.

The remaining individuals all fall within the overlap between the ceramic final Later Stone Age and the Iron Age (SI Fig. 1). The oldest of these (flo029) is another woman from the Free State found at Marokasvlug in 1960 with ostrich eggshell beads, no further archaeological context is available (Morris, 1992a). Flo027 is of similar age, and her cranium and mandible, together with some beads, were excavated in 1936 from what was described as a "typical Bushman/San grave" near Koffiefontein in the Free State with no further information (Morris, 1992a). The Koffiefontein burials, however, form part of the Riet River group as discussed above (Morris, 1992b). The description of grave goods for other Koffiefontein burials is in line with a ceramic final Later Stone Age context for this woman (Humphreys, 1970). Near Hartenbos in the Western Cape, a man (flo015) was buried in a sand dune with a small pot and no further context provided (Morris, 1992a). However, based on the presence of pottery, and the fact that no Iron Age settlements are known from the region, we may accept that this person was associated with the ceramic final Later Stone Age phase (Loftus *et al.*, 2024). The man (flo032) from Renosterkop near Augrabies in the Northern Cape was excavated in 1936 from a large oval grave of a meter deep, where he was found with a small copper earring similar to those of other Khoekhoe people of the region (Morris, 1981, 1992a), a ceramic final Later Stone Age designation can thus be inferred. Although the cranium and mandible of a woman (flo031) from Houdenbek and a fairly complete skeleton of a man (flo033) from Stinkwater near Abrahamsdam, both in the Northern Cape, are void of any archaeological context (Morris, 1992a), their radiocarbon dates and location on the landscape suggest a ceramic final Later Stone Age association rather than an Iron Age one. The skeleton of another woman (flo030) was excavated in 1935 from a "typical Bushman/San" grave near Vaalbank on the Riet River in the Fee State, and with her she had an iron object (Morris, 1992a), it is therefore not possible to ascribe her to either the ceramic final Later Stone Age or the Iron Age.

The two men from Plovers Lake in Gauteng (plo001, plo002) were previously thought to date from the Middle Stone Age (de Ruiter *et al.*, 2008). We have since shown that their mitochondrial DNA corresponds with that of Iron Age farmers, and that their diet was similar to that of Iron Age farmers from the same region (Lombard *et al.*, 2019). What is more, the DNA of bovids

from the same context is consistent with that of South African Nguni cattle (Lombard *et al.*, 2019), instead of with that of the Cape Buffalo as previously identified by de Ruiter *et al.* (2008). Their associated radiocarbon dates place them in the Late Iron Age comprising several phases that take their names from associated ceramic styles, and are associated with iron production, and a cattle-and-crop economy (Boeyens, 2003; Huffman, 2007).

1.2 The Matjes River individuals in archaeological context

Dreyer (1933) started to excavate the Matjes River Rock Shelter during the 1920s, followed by Hoffman and Meiring during the 1950s (Hoffman, 1958; Louw, 1960). These excavations removed several tons of soil, > 30,000 artefacts, and numerous skeletons and fragmentary human remains (L'abbe *et al.*, 2008). Because most work focused on the human remains (Dreyer, 1931, 1933; Keith, 1933; Meiring, 1937; Hoffman, 1962; Clayton *et al.*, 2006; Neuweger, 2007), interpretation of the archaeological material remains only published in the form of theses (e.g., Louw, 1960; Ludwig, 2005). There is, however, broad consensus about five stratigraphic layers named A-E from youngest to oldest.

Layer A is the only context that contains a few pieces of pottery (Louw, 1960), which is usually associated with the arrival of, or contact with, Stone Age dairying communities in southern Africa (e.g., Breton *et al.*, 2014). Currently this phase is referred to as the ceramic final Later Stone Age in southern Africa and usually dates to <2,000 years ago in South Africa (Lombard *et al.*, 2013). Layer B is associated with a thick shellfish deposit (Hoffman, 1958) that is comparable to other mega-middens of the Cape Holocene (e.g., Jerardino, 2007, 1998). Ludwig (2005)'s description of the material culture of the layer is consistent with what we currently refer to as the final Later Stone Age in southern Africa. Such assemblages can date to as young as 100 years ago, reaching back in time to ~4,000 years ago. They are associated with a hunter-gatherer economy with mostly informal lithic assemblages augmented by abundant worked-bone assemblages (Deacon, 1984; Lombard *et al.*, 2013). At Matjes River, Döckel (2007) interpreted this phase to potentially represent a so-called "strandloper" subsistence of impoverished herders who relied on shellfish and fish when their herds had been depleted, or a "watermans" foraging group who possessed no stock but moved seasonally to the coast liv-

ing on the edge of starvation and subsisting on shellfish and decaying meat (e.g., Raven-Hart, 2007; Döckel, 2007).

Layer C is generally associated with the blade-based Wilton technocomplex thought to have spanned ~4,000 to ~8,000 years ago, and associated with well-developed hunting technologies in a hunter-gatherer economy (e.g., Lombard, 2020a,b). These assemblages contain fully developed microlithic stone tool technologies with frequent formal artefacts (Deacon, 1984; Lombard *et al.*, 2013). Layer D is associated with the Albany variant of the Oakhurst technocomplex, generally dating in southern Africa to between ~7,000 and ~12,000 years ago. Oakhurst lithic assemblages are flake-based, indicating a clear technological shift to the blade-based industries of the subsequent Wilton phase. These assemblages also usually include a wide range of polished bone tools as recorded in the large quantity of such artefacts from Layer D at Matjes River. Layer E was initially thought to contain Middle Stone Age artefacts, but the assemblage is too small and undiagnostic to reach any conclusion (e.g., Ludwig, 2005). SI Fig. 1 illustrates how the human remains sampled for this study relates to the Holocene Later Stone Age of South Africa (Lombard *et al.*, 2013), with material from each of the phases.

1.3 Relationship of ancient southern Africans and modern-day indigenous Khoe-San groups

African hunter-gatherer populations capture the deepest divergences among modern humans (Gronau *et al.*, 2011; Schlebusch *et al.*, 2012, 2017, 2020; Breton *et al.*, 2021), and of these, the southern African indigenous Khoe-San populations represent the earliest diverging branch in the population tree of living peoples (Veeramah *et al.*, 2012; Schlebusch, 2010; Schlebusch *et al.*, 2020; Schlebusch & Jakobsson, 2018). The San and Khoekhoe communities of present-day southern Africa are remnant groups of a larger and more widely distributed population of hunter-gatherers and pastoralists who occupied southern Africa before the migration of Bantu-speaking peoples and East African pastoralists within the past 2,000 years, and the more recent influence of colonialism within the past 400 years (Schlebusch *et al.*, 2017).

Contemporary Khoe-San populations are genetically distinct from each other,

with a clear geographic structuring among groups (Montinaro *et al.*, 2017; Schlebusch *et al.*, 2012; Vicente *et al.*, 2019; Schlebusch *et al.*, 2020). Northern San (K'xa speakers from southern Angola and northern Namibia) and southern San (Tuu speakers from southern Botswana and South Africa) display the highest levels of genetic stratification, with central San groups (Kalahari-Khoe speakers from Botswana) being intermediate. Traditionally nomadic pastoralist herding groups such as the Nama (Khoekhoe speakers from Namibia), show some level of admixture with East African populations. This East African ancestral component has been traced to the introduction of pastoralism to southern Africa between 2,000 and 1,200 years ago (Schlebusch *et al.*, 2017; Vicente *et al.*, 2019; Breton *et al.*, 2014; Coutinho *et al.*, 2021; Skoglund *et al.*, 2017; Vicente *et al.*, 2021). The sequencing of the genome of an ancient southern African individual from ∼2,000 cal. BP provided evidence that a later admixture introduced this East African genetic component to Khoekhoe herding groups (Schlebusch *et al.*, 2017).

2 Supplementary material & methods

2.1 Sampling and permits

To increase our understanding of southern African Later Stone Age peoples, we mainly collected samples from human remains from presumed hunter-gatherer associated sites, and sampled both in a time-depth fashion (at Matjes River) and on a wide geographical scale. The majority of the samples (labelled "flo") were housed by the National Museum of Bloemfontein at Florisbad Quarternary Research Station, Free State, South Africa. Permission to sample human remains were approved by Eastern Cape Provincinal heritage Resources Authority (no. 2/2APM-PERMIT/15/03/002-) and Heritage Western Cape (no. 14120409GT0812E) and permission for export and destructive sampling was approved by the South African Heritage Resources Agency (SAHRA no. 1987). Remaining samples (labelled "plo" and "tob") were housed at the School of Anatomical Sciences and Evolutionary Studies Institute, University of Witwatersrand, Gauteng, South Africa. Permission for sampling (SAHRA no. 1934) and export (SAHRA no. 1935) were approved by the South African Heritage Resources Agency. Sampling was done on site and the skeletal remains were immediately returned.

2.2 DNA and isotope data collection

Two to three samples were taken from each of 28 individuals (or museum accession number), the majority of which were from different bone elements, for ancient DNA analyses, radiocarbon dating and stable isotope analyses (Supplementary Data 1-2). The bone elements were UV irradiated (254 nm) for 30 minutes to one hour per side and stored in plastic zip-lock bags until sampled. Further handling of the specimens was done in a bleach-decontaminated (DNA Away, ThermoScientific) enclosed sampling tent with adherent gloves (Captair Pyramide portable isolation enclosure, Erlab). Teeth were wiped with 0.5% bleach (NaOH) and UV-irradiated sterile water (HPLC grade, Sigma-Aldrich). The outer surface was removed by drilling at low speed using a portable Dremel 8100, and between 60 and 200 mg of bone powder was sampled for DNA analyses from the interior of the bones and teeth. All plastics and equipment used had been decontaminated with DNA-away and/or UV irradiation prior to their use. The researchers wore full-zip suits with caps, face-masks with visors and double latex gloves and the tent was frequently cleaned with DNA-away during sampling. A total of 20 of the 28 individuals were sampled for AMS radiocarbon dating either through cutting off a small piece of bone (1.8-4 cm) or through drilling out bone powder (600-750 mg). Previous radiocarbon and stable isotope measurements was available for five of the individuals (Lombard *et al.*, 2019; Sealy *et al.*, 2006) and for three individuals (tob001, tob002 and tob005), only associated human remains was dated. The sampling was conducted on-site, either at Florisbad Quarternary Research Station or at the School of Anatomical Sciences at Wits Medical School at the University of Witwatersrand and all the sampled bone elements were directly returned to the Museum and the ancient DNA samples and radiocarbon samples were transported to the Ancient DNA Laboratory at Uppsala University, Sweden. Samples were also sent to Beta Analytic where bone collagen extraction were performed for Accelerator Mass Spectrometry (AMS) radiocarbon dating and for stable dietary isotope analyses using Isotope ratio mass spectrometry (IRMS). Conventional radiocarbon ages were corrected for isotopic fraction using the δ^{13}C and the modern reference standard was 95% of the 14C signature of NBS SRM-4990 (oxalic acid) and calculated using Libby 14C half life (5568 years) with errors represent 1 sd statistics (68% probability) counting errors based on the combined measurements of the sample, background and modern reference standards. IRMS values are reported as δ^{13}C relative to the PBD-standard

(± 0.3 ‰) and δ^{15}N relative to the AIR-standard (± 0.5 ‰). Conventional radiocarbon dates obtained from Beta were modelled using BetaCal 3.21 and SHCal13 (Hogg *et al.*, 2013) while radiocarbon ages for three previously dated individuals were modelled using OxCal v.4.4 and SHCal20 calibration curves (Ramsey, 2009; Hogg *et al.*, 2020) (Supplementary Data 2). We avoided to correct for marine reservoir effects (MRE) due to the large variability in MRE for different individuals across time and for different coastal subregions, similar to previous studies (Döckel, 2007; Richards & Hedges, 2003; Sealy, 2006; Alves *et al.*, 2019).

DNA was extracted either as in Yang *et al.* (1998) with modifications as in Malmström *et al.* (2007) or as in Dabney *et al.* (2013). DNA extracts were prepared from the 28 human remains (between 1-7 DNA extracts from each individual). One blunt-end library was prepared for each DNA extract and sequenced for screening of endogenous human DNA (Meyer & Kircher, 2010; Günther *et al.*, 2015). The presence of authentic ancient DNA ($> 1\%$ human DNA) was established in 32% of the sequencing libraries that were used for screening. For DNA extracts where the proportion of human ancient DNA were $> 2\%$, sequencing libraries were prepared using UDG treatment to minimise post-mortem deaminations and increase sequence depth (Briggs & Heyn, 2012). For DNA libraries where the proportion of human ancient DNA was $<2\%$, additional blunt-end libraries were prepared in order to increase sequence depth. Due to low amounts of endogenous human DNA, some of the libraries were enriched using MYbait African Human Whole Genome Capture Kit (MYcroarray) following the manufacturer´s instructions (MYbaits manual version 2.3.1) and amplified as in Schlebusch *et al.* (2017). This enrichment was performed on one out of four blunt-end libraries for Tobias Cave 1 (tob001), 11 out of 21 blunt-end libraries for Tobias Cave 5 (tob005), two out of three blunt-end libraries and one out of three UDG-treated libraries for Plovers Lake 1 (plo001) and two out of five blunt-end libraries and two out of two UDG-treated libraries for Plovers Lake 2 (plo002).

2.3 Sequencing and data processing

All libraries were sequenced on either an HiSeq X10 or a NovaSeq 6000 (SP flow cell) Illumina sequencer with either 100 bp or 150 bp paired-end chemistry (Supplementary Data 1). The raw fastq sequences were prepared for downstream analysis by first trimming off adapter sequences and, if an lover-

lap of at least 11bp was detected between forward and reverse read, merging paired-end reads using either the script MergeReadsFastQcc.py (Kircher, 2012) (before September 2017) or Adapter Removal v2.1.7 (Schubert *et al.*, 2016) (after September 2017). Subsequently the merged and trimmed reads were mapped against the human reference genome build 37 (hs37d5) using bwa aln (-l 16500 -n 0.01 -o 2) (Li & Durbin, 2009; Lazaridis *et al.*, 2014; Skoglund *et al.*, 2014). In order to remove PCR duplicates, reads with identical start and end positions were identified and collapsed using a slightly modified version of FilterUniqeSAMCons.py (Kircher, 2012). Reads shorter than 35bp and with <90% consensus with the reference sequence were filtered out using percidentity_threshold.py (Skoglund *et al.*, 2012) and genetic sex was determined using the X/Y ratio (Skoglund *et al.*, 2013).

In order to ensure maximal retention of reads, sequence data were merged to library level using samtools merge v0.1.19 (Li *et al.*, 2009) before removal of PCR duplicates. Non-UDG and UDG-treated libraries were then separately merged per individual before pseudohaplodized genotype calling was performed. Variants from the human genome diversity project (HGDP) (Bergström *et al.*, 2020) lifted to hg19 were selected as known variants to call genotypes from the ancient DNA data. For non-UDG treated sequence data, all transition (C/T and A/G) sites were coded as missing data to avoid effects of post-mortem damage. For those sequenced individuals for whom we had both UDG-treated and non-UDG treated libraries, a read from either of the two libraries was randomly sampled for transversion sites, and only reads from damage-repaired libraries were sampled from transition sites. At each SNP site, a random read with a minimum mapping and base quality of 30 was drawn and the allelic status at that read was coded to be the hemizygous genotype of the individual. Sites showing more than two alleles or indels were removed from the data.

Two different methods were used to estimate mitochondrial contamination; Green *et al.* (2008) and contamMix (Fu *et al.*, 2013) (Extended Data Table 1 and Supplementary Data 1). Additional contamination estimates were performed on all individuals with genome-wide coverage >2x. VerifyBamID checks whether reads are contaminated as a mixture of two samples (Jun *et al.*, 2012), and all these individuals had estimated contamination-levels <3.2% (Supplementary Data 5). In all cases the autosomal and mitochondrial contamination estimates were concordant, showing low levels of contamination (<5%, Supplementary Data 1 & 5).

The six ancient southern African individuals with coverage > 7x used in this study (Matjes River 1, Matjes River 3, Matjes River 11, Great Brak River Cave, Cape St Francis and Springbokvlakte), as well as comparative high coverage ancient genomes from southern Africa (n=1) (Schlebusch *et al.*, 2017), eastern Africa (n=1) (Llorente *et al.*, 2015), western Africa (n=1) (Lipson *et al.*, 2020), northern Africa (n=1) (Simões *et al.*, 2023), pre-Neolithic Eurasia (n=7) (Günther *et al.*, 2018; Fu *et al.*, 2014; Jones *et al.*, 2015; Lazaridis *et al.*, 2014; Simões *et al.*, 2024) and archaic genomes (n=4) (Prüfer *et al.*, 2014; Meyer *et al.*, 2012) were subjected to diploid genotype calling on a per chromosome basis using snpAD (Prüfer, 2018). We will refer to this set of 21 genomes as the "complete ancient genomes". Before genotype calling, base quality recalibration was performed on the five outermost bases of each read by reducing the quality of all T's on the 5' end and all A's on the 3' end, to Phred score 2 (#). Picard v1.118 (http://broadinstitute.github.io/picard/) was used to add read groups (RG) to the bam files ("blunt" to non-UDG treated bam file and "dr" to UDG treated bam file) and local realignment around indels was performed with GATK v3.5.0 (Van der Auwera *et al.*, 2013). For individuals with both non-UDG treated and UDG treated bam files, the files were merged to a single bam file that was used for the actual genotype calling. In order to estimate different error profiles for non-UDG and the UDG reads of merged individuals, we set the parameter --offset 31 for all reads with RG "dr" in the Bam2snpAD step. The raw VCF files were then filtered so that only regions with unique mappability of 35 bp were kept (UCSC wgEncodeDukeMapabilityUniqueness35bp.bigWig) and where positions had a covering depth (DP) of at least 4 and a quality of at least 30. All samples VCF files, on a per chromosome basis, were then merged and annotated with dbSNP version 142 using BCFtools annotate (Danecek *et al.*, 2021). The command LiftoverVCF implemented in picard v3.1.1 (`http://broadinstitute.github.io/picard/`) and the chain file "hg19ToHg38.over.chain.gz" from UCSC was used in order to create a version of the dataset in the GRCh38 reference coordinate system. Positions that switched chromosome were removed before downstream analyses.

2.4 Inference of uniparental haplogroups

Mitochondrial haplogroups were confidently inferred for all individuals with Haplogrep and Phylotree Build 17 (Weissensteiner *et al.*, 2016; Van Oven,

2015). Where available, damage repaired libraries were used, except for Houdenbek (flo031) in which the coverage and support for the determined haplogroup was higher using the merged blunt end library. Mitochondrial haplogroups from the Plover's Lake individuals were previously published (Lombard *et al.*, 2019).

Y-chromosome haplogroups were assigned by using Samtools v.1.3 (Li *et al.*, 2009) mpileup to call single base substitutions from Phylotree (version 09/03/2016, `http://www.phylotree.org/Y/tree/`) (van Oven *et al.*, 2014) from bam files mapped to hs37d5 (hg19). Sites with mapping quality and base quality of at least 30 were extracted. Indels, transitions and A/T and C/G SNPs were excluded to account for deamination damage and strand missidentification.

2.5 Comparative genomic data

To investigate population stratification and genetic affinities among individuals, we compiled a genome-wide dataset of all ancient southern Africans in this study (Extended Data Table 1) merged to comparative modern-day individuals (Mallick *et al.*, 2016; Bergström *et al.*, 2020; Choudhury *et al.*, 2017; Fan *et al.*, 2019; Schlebusch *et al.*, 2012, 2020) (Supplementary Data 8) and published ancient African individuals (Llorente *et al.*, 2015; Skoglund *et al.*, 2017; Wang *et al.*, 2020; Schlebusch *et al.*, 2017; Lipson *et al.*, 2020, 2022; Prendergast *et al.*, 2019; Gurdasani *et al.*, 2015; Coutinho *et al.*, 2021) (Supplementary Data 7). Published ancient African sequence bam files were downloaded and processed with the same pipeline as described above, while comparative modern-day whole genome sequencing data was processed as follows: Genomic VCF-files were downloaded where available. The data was lifted from hg38 to hg19 if applicable, and positions that switched chromosomes or ended up as duplications were removed from further processing. The data was filtered for 10% missingness and Hardy Weinberg equilibrium with a p-value of 0.000001. The genomic VCF files from Schlebusch *et al.* (2020) were additionally filtered for quality and 10% missingness prior to processing.

For high-coverage ancient diploid analyses, we used two versions of the Phase 3 data from the 1000 Genome project (KGP). A filtered version of the dataset in hg19 coordinates was downloaded from `https://hgdownload.soe.ucsc.edu/gbdb/hg19/1000Genomes/phase3/`, while the CRAM files, in GRCh38

coordinates, for 8 randomly collected individuals per population were downloaded from ENA (a total of 208 individuals, see Supplementary Data 8). Diploid genotype calling was then performed according to Schlebusch *et al.* (2020). In order to retrieve the full spectra of genetic variation in present-day Southern Africa, Khoe-San populations (Ju|'hoansi, Nama, !Xun, Karretjie people, and Gui and Ghana) from Schlebusch *et al.* (2020) were also used as comparative data. Details of the processing and diploid genotype calling can be found in Schlebusch *et al.* (2020).

2.6 Filtering genome-wide data

We assess population stratification with model-free (Multi Dimensional Scaling/Principal Coordinate Analysis/Principal Component Analysis, MDS/PCoA/PCA), "mild-model-assumption" (Admixture), and "explicit-topology-model" (*f*-statistics) approaches. After initial data-summaries using PCA that detect a number of outliers among the ancient southern Africans (including tob001, plo001, plo002, flo006, flo019, flo027), the genome-wide data was filtered with PLINK v.1.9 (www.cog-genomics.org/plink/1.9) for a minimum allele frequency of 10% and LD pruned using command --indep-pairwise 200 25 0.7. All modern-day individuals were further pseudohaplodized prior to population stratification analyses.

2.7 Assessing stratification with MDS/PCoA/PCA

The PCoA was performed with PLINK --pca, which bases the dimension reduction estimate on the variance-standardized relationship matrix rather than the genotype matrix itself as used in Principal Component Analyses (PCA). PCA can prone to biases for high levels of missing data, which has resulted in the practice of projecting ancient individual on-top of principal components build from modern-day individuals. This practice is a compromise between the possibility to visualize genetic affinities for low-coverage ancient individuals and limiting the outcome to modern-day genetic variation. For most studies of relatively recent, and non-African, ancient humans, this compromise works well since the modern-day comparative data is rich (e.g., 1000 Genomes Project Consortium, 2015). In order to compare the results of a MDS/PCoA and PCA, we also performed both unprojected and projected PCA (with parameter lsqproject: YES) with the software smartpca from the Eigensoft package (Price *et al.*, 2006; Patterson *et al.*, 2006)

and the non-default parameters r2thresh: 0.7 and shrinkmode: YES (compare SI Fig. 2 and 3, also with main text Fig. 2A and Extended Data Fig. 1). Projecting the ancient genome variation onto axes build on modern-day variation creates a severe bias towards modern-day variation, leaving an impression of overlapping variation among e.g. modern-day Khoe-San and ancient southern Africans.

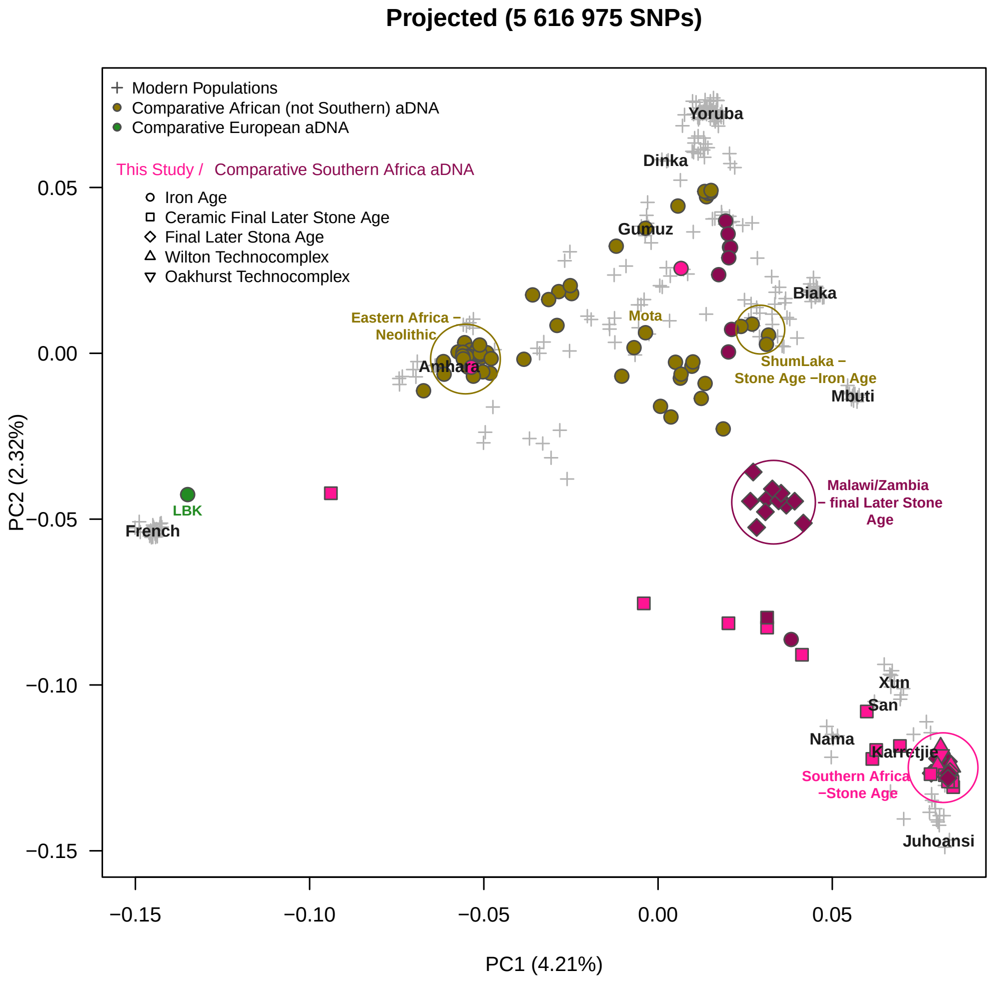

Supplementary Information Figure 2: Principal Component analysis (PCA) using the software smartpca. Ancient samples are projected on top of the modern variation using the parameter lsqproj: YES

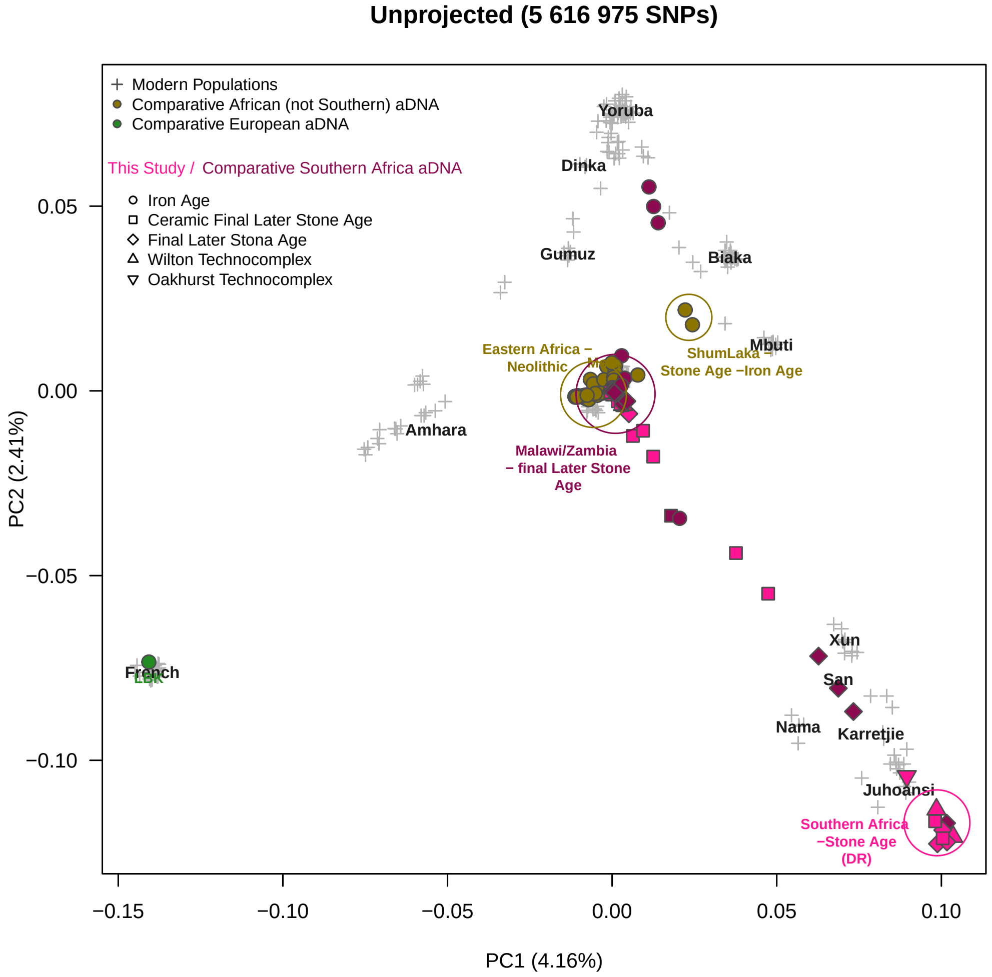

Supplementary Information Figure 3: Principal Component analysis (PCA) using the software smartpca. Components are estimated on both the ancient and modern samples using the parameter lsqproj: NO.

2.8 Estimating ancestry components

The program ADMIXTURE v1.3.0 (Alexander *et al.*, 2009) was used for unsupervised estimation of ancestry components. A total of 25 iterations were run for each value of assumed number of clusters (K=2−10), and ADMIXTURE results were summarized using PONG (Behr *et al.*, 2016) and

visualized with a custom R-script.

2.9 Tests of genetic affinity with f-statistics

To investigate genetic relationships among the ancient southern African hunter-gatherers and each of the modern-day Khoe-San populations, outgroup-f_3 statistics were performed to test for degree of shared drift among samples (using Chimpanzee and Denisovan as outgroups). In addition, the results of pairwise outgroup-f_3 tests for all ancient samples were summarized in a distance matrix (Euclidean) and pairwise-distances visualized as a heatmap using R package "pheatmap".

In order to specifically test for signals of gene-flow from eastern Africa into ancient southern Africans, we use f_4-tests of the form f_4(Denisovan, [eastern Africa]; *test*, Matjes River 1), where we use both ancient eastern Africans (Mota) and modern-day eastern Africans (Amahara) as the eastern African source, and we use Matjes River 1 as baseline for ancient southern Africans (the oldest high-coverage genome), Extended Data Fig. 6A-B. A similar set-up is used for testing for western African gene-flow into ancient southern Africans (Extended Data Fig. 6C-D).

To further investigate potential gene-flow, we contrast genetic affinity of ancient southern Africans to other African groups using an F_4-test of the form F_4(D,aSA,aEA,aWA). This test-statistic would be negative if there is gene-flow between ancient southern Africans (aSA) and ancient eastern Africans (aEA), or positive if there is gene-flow between ancient southern Africans and ancient western Africans (aWA). See SI section 3.5 and SI Fig. 5 for results of these tests.

To minimize bias introduced by ascertainment when performing f-statistics, only alleles that are polymorphic between the Altai Neanderthal and Denisovan samples were investigated (Skoglund *et al.*, 2017) (approximately 500,000 sites). The program popstats (Skoglund *et al.*, 2015) was used to estimate f_4 statistics and $f3$ statistics using the –f3vanilla option.

2.10 Correlations between genetic and geographic distance, and time

We estimated the correlation between (i) genetic distance and (ii) geographic distance or (ii) difference in chronological time (difference in radiocarbon dates). We estimated genetic distance by computing the number of differences between 2 individuals' pseudohaplodized genomes (similar to the computation of pairwise conditional nucleotide diversity (CND), in (Skoglund *et al.*, 2014)). This computation was conducted for the dataset described in section 2.6, and restricted to the set of ancient southern Africans. Geographic distance between sample locations was calculated using the R-package "geosphere" and the function "distGeo" which converts two sets of geographic coordinates (longitude and latitude) into a distance estimate in meters. Correlation between the genetic distance and geographic distance and time was then estimated using a linear regression model.

2.11 Assessing effective population size over time with MSMC

The highest coverage ancient southern African hunter-gatherer individual, Matjes River 11 woman (flo011) and the Ballito Bay A boy genome (Schlebusch *et al.*, 2017) were used as input to multiple sequentially coalescent (MSMC v0.1.0) (Schiffels & Durbin, 2014) in order to estimate changes in effective population size over time. When a single diploid genome per population is used as input to MSMC, phasing is not necessary. Genomic regions considered accessible for short paired end reads were obtained from the 1000 genomes project (`ftp://ftp.1000genomes.ebi.ac.uk/vol1/ftp/phase1/analysis_results/supporting/accessible_genome_masks/20120824_pilot_style_mask.bed`). Default parameters were used alongside a fixed recombination rate and a recombination to mutation rate ratio of 0.88. When plotting results, a mutation rate of 1.45×10^{-8} and generation time of 29 years were assumed. The preparation of the genome data follows (Schlebusch *et al.*, 2020). Please see (Schlebusch *et al.*, 2020; Simões *et al.*, 2023) for estimates of N_e as a function of time from comparative genomes from other regions, including modern-day Khoe-San, and ancient northwestern Africa.

2.12 Runs of Homozygosity

Runs of Homozygosity (RoH) were calculated with PLINK v1.9. A sliding-window approach was used observing a protocol previously used for estimating RoH in ancient African samples (Schlebusch *et al.*, 2017). RoH were calculated for each of 14 ancient humans in a bi-allelic filtered version of the "complete genome dataset" (7 South African, 4 European and 3 West-East and North African) as well as for a filtered version of KGP and modern day Khoe-Sans datasets using the following parameters: "--homozyg-snp 200" sets the minimum required number of SNPs to be classified as RoH at 200, "--homozyg-kb 200" sets the length of the sliding window to 200 kb, "--homozyg-density 20" sets a required minimum density of 1 SNP per 20 kb, "--homozyg-window-snp 50" sets the number of SNPs the sliding window must have, "--homozyg-gap 50" sets the length between two SNPs to be considered two different segments to 50 kb, "--homozyg-window-het 1" sets the number of heterozygous SNPs allowed in each window to 1, "--homozyg-window-missing 15" sets the number of missing calls allowed in a window to 15, and "--homozyg-window-threshold 0.05" sets the proportion of overlapping windows that must be called homozygous to define a given SNP as occurring in a "homozygous" segment. We calculated mean RoH size, sum of RoH segments and total length of *RoH* and results were plotted (Extended Data Fig. 4) using custom R scripts.

2.13 Genetic diversity within and between complete ancient southern African genomes

Summary statistics of diploid genotype calls for the high quality ancient human genomes and archaic human genomes was estimated on a per chromosome basis with bcftools stats (Danecek *et al.*, 2021) and the "-s -" parameter set. Per individual heterozygosity level (main text Fig. 3A) was estimated as the number of heterozygous genotype calls over total number of genotype calls ("HomRef", "Het", "HomAlt") using custom R script.

Pairwise genetic differences was estimated for all combinations of eleven ancient human genomes (4 South African; 4 European and 3 West- East- and North Africans) and 4 archaic human genomes with genome coverage >10x (Extended Data Fig. 5). A pseudohaploized genome of a Chimpanzee were also included in order to root the estimates outside of the Hominid

branch. Per chromosome distance matrices were estimated using the software VCF2Dis (Xu *et al.*, 2025) and further processed in R v.4.2.0 (R Core Team, 2021). Chromosome length differences were handled by weighting each chromosome by its contribution to the autosomal genome (chromosome length / total autosomal length). A UPGMA clustering approach, using the package "phangorn" and the "average" agglomeration method, was used in order to display the pairwise differences and cluster the individuals into a bifurcating genetic distance tree (main text Fig. 2D).

2.14 Estimating genetic differentiation with F_{ST}

In order to estimate how different the ancient South African population is from modern-day Khoe-San populations, F_{ST} values were estimated between the high-coverage ancient South African population (n=7) and five modern-day Khoe-San populations (n=5 per population). To compare this genetic differentiation to a global level, F_{ST} values were also computed on all possible combinations of populations from the 1000 genomes project (325 comparisons), but where we randomly draw 8 individuals per population to reduce the effect of sample size. The analyses were conducted with VCFTools v0.1.16 (Danecek *et al.*, 2011) using the --weir-fst-pop parameter (set twice, one per each population in comparison), which estimates Weir and Cockerham's (Weir & Cockerham, 1984) fixation index on a per-site basis. By estimating the fixation index per site (all called sites), the results will be addable over all chromosome based VCF files. To obtain a genome-wide average estimate of F_{ST}, we sum the individual-site F_{ST}s and divide by total number of sites.

2.15 Examining the frequency spectra of full genomes and amino-acid altering sites

Amino-acid altering variants (mis-sense mutations) are potentially important for conveying biological functions in any organism, including humans. In order to investigate amino-acid altering variants unique to the human lineage, we determined the ancestral and derived state by comparing to the genomes of three Great Apes. To further assess the distribution of variants that are unique for humans, we compared with the four high-quality archaic genomes (three Neandertals and one Denisovan; Prüfer *et al.*, 2014; Reich *et al.*, 2010; Mafessoni *et al.*, 2020; Green *et al.*, 2010), and look for variants

where all the archaic individuals are fixed for the ancestral variant, and humans have (at least one) derived variant (Zeberg *et al.*, 2024). We computed the site-frequency-spectra (SFS) for all biallelic SNPs as well as for amino-acid altering variants that were (i) fixed for the ancestral variant among the four archaic genomes, and (ii) fixed for the derived variant among the four archaic genomes. In order to compare the SFS from seven ancient southern Africans to other populations, we both estimated the SFS over all 26 populations from the 1000 Genomes set of individuals (1000 Genomes Project Consortium, 2015), as well as per population for western Africa (YRI), European descent (CEU) and East Asia (JPT). We also compared to all three high-quality ancient genomes from other parts of Africa (northern (Simões *et al.*, 2023), western (Lipson *et al.*, 2020) and eastern (Llorente *et al.*, 2015)) and seven high-quality genomes from pre-Neolithic Eurasians (Simões *et al.*, 2024; Fu *et al.*, 2014; Günther *et al.*, 2018; Lazaridis *et al.*, 2014; Jones *et al.*, 2015) as well as seven individuals from the Ju|'hoansi population and five individuals from the Karretjie people population (Schlebusch *et al.*, 2020). Biallelic SNPs (lifted over to GRCh38) of this high-coverage dataset were annotated with SnpEff (Cingolani *et al.*, 2012) for functional effect (with annotated effect "missense_variant" using VCFtools –freq command), using the "hg38kg" genome supported by the program. For each set of individuals, allele frequencies were estimated for all such amino-acid altering positions.

The ancestral state was determined for all variable sites by assessing the genomes of three Great Apes (gorilla, orangutan and chimpanzee). Only sites with data from at least one Great Ape and consensus among the Great Apes were analysed further. We further restricted our analyses of both the full-genome spectra as well as the amino-acid altering sites to where the 4 archaic genomes (three Neanderthals and one Denisovan) were fixed (either for the ancestral or the derived variant). For each group, we plotted the SFS for derived variants that were fixed for the ancestral variant among four archaic genomes (amino-acid altering variants in main text Fig. 4A; full variable spectra in Extended Data Fig. 7A). Among all the sites, we computed the frequency of sharing among different groups (including ancient southern Africans, pre-Neolithic Eurasian, modern-day Khoe-San (Schlebusch *et al.*, 2020) and a representative set of 8 individuals from 26 populations from the KGP dataset (1000 Genomes Project Consortium, 2015) as a Venn-diagram (using the R-package "VennDiagram" (Chen & Boutros, 2011)) and custom

R scripts.

GO-term enrichment analyses were performed by linking amino-acid altering variants to genes and then uploaded the gene list to WEB-based GEne SeT Analysis Toolkit (`https://www.webgestalt.org`), using the "Over-Representation model" for *Homo sapiens*. As functional database we used geneontology for Biological Processes using the reference set "genome". We used default parameter settings except changing "significance level" from 10 to 15.

2.16 Assessing population continuity

To investigate patterns of population continuity among ancient southern African hunter-gatherer individuals, we used an approach developed by McKenna *et al.* (2024). This method conditions on heterozygous sites in the oldest (called the "anchor") individual and counts the proportion of derived alleles occurring at those sites in more recent individuals. Genetic drift does not affect this statistic forwards in time from the population the anchor was sampled from, but causes it to decrease backwards in time. Therefore, an unchanging proportion forwards in time from the anchor indicates continuity with the anchor population, while a reduction in proportion in a more recent individual indicates that individual has been sampled from a population diverging prior to the anchor, or that an admixture event from such a population has occurred between the anchor population and the sampled individual. The oldest high-coverage individual, Matjes River 1 (approximately 7,800 calBP), was used as the anchor and levels of continuity were assessed among all ancient South African individuals, both from this study and previously published. Heterozygote positions with known ancestral state (based on the three Great Apes gorilla, orangutan and chimpanzee) were extracted from the anchor individual's VCF-files. Samtools mpileup (Danecek *et al.*, 2021) with parameter set: −q 30 −Q 30 −R "het.positions.txt" was then used to summarise the overlapping reads in test individuals' BAM files, were the probability of drawing the ancestral allele was used for the analysis.

2.17 Estimating population divergence times

Estimates of population divergence time among individuals in the "complete (>7x) ancient genomes" dataset were obtained using the Two-Two-outgroup

(TTo) site-frequency-based method (Sjödin *et al.*, 2021). The method estimates population divergence times (in generations) from complete genomes (so that diploid genotypes can be called for the full genome) from a pair of individuals (one individual per population), returning independent estimates for each branch in a population split that is unaffected by genetic drift. The approach assumes an infinite-number-of-sites-model, small mutation rate per site, independence between sites and a pure split model with no gene-flow between branches. The TTo approach has been shown to give robust divergence time estimates despite modest levels of gene-flow (Sjödin *et al.*, 2021). By ascertaining using an outgroup (here we have used both Denisovan and Neandertal), the TTo method does not need to assume a constant ancestral population size, removing possible bias in divergence time estimates related to population bottlenecks in the ancestral population (Sjödin *et al.*, 2021). A weighted block jackknife procedure with 5 Mb blocks is used to estimate the confidence intervals of estimates. In order to rescale the estimated divergence times from generations to chronological years, a mutation rate of $1.45{\times}10^{-8}$ (per base-pair per generation) and a generation time of 29 years was used.

Comparative modern genomes used for divergence time estimations were from the Simons Genomes Diversity Project (SGDP) (Mallick *et al.*, 2016), the Human Genome Diversity Project (HGDP) (Bergström *et al.*, 2020) and the KSP (Schlebusch *et al.*, 2020). Comparative data comprising ancient African genomes were chosen to represent as temporally and geographically broad a sample as possible, and included the ShumLaka (Lipson *et al.*, 2020), Mota (Llorente *et al.*, 2015), Ballito Bay boy (Schlebusch *et al.*, 2017) and Ifri Ouberrid (Simões *et al.*, 2023). VCFs were filtered to pass only biallelic sites with a QUAL>30 and a reference or alternative allele matching the ancestral state present in all three Great Apes (gorilla, orangutan and chimpanzee). For a site to be considered informative, it must pass allele depth thresholds set by the lower and upper 5% of site coverage distributions, and a minimum allele depth of 4 in all compared individuals.

3 Supplementary results

3.1 Dietary analysis and radiocarbon dating

Twenty of the samples had not previously been radiocarbon dated and were therefore directly radiocarbon dated successfully for this study and measured for the dietary - indicating stable carbon and nitrogen isotopes, while a different and loose tooth was used to represent the Tobias Cave individuals as we were not permitted to directly date Tobias Cave 1, 2 and 5 (Supplementary Data 2).

Humans are omnivores and generally consume quite diverse foods making dietary inferences quite challenging. There are, however, some useful patterns among δ^{13}C and δ^{15}N values that can be used to infer prehistoric food consumption. For example, low δ^{13}C values are indicative of a high intake of terrestrial C_3-based foods such as meat from browsing animals, fruits and nuts and freshwater fish (Schoeninger *et al.*, 1984; Sealy & Van Der Merwe, 1986) while the highest values indicate C_4-based foods such as meat from grazing animals, sorghum and millet followed by marine food sources (Schoeninger *et al.*, 1984; Sealy & Van Der Merwe, 1986; Van Der Merwe, 1982; Ribot *et al.*, 2010). The δ^{15}N value, on the other hand, increase with each trophy level in the food chain and is lower in terrestrial foods while being especially high in marine mammals that are high up in the food web (Minagawa & Wada, 1984).

To assess the general dietary trend of the ancient southern Africans, we collated comparative isotope data from several African groups including farmers, foragers and pastoralists from different bioregional contexts for which comparable data are available (SI Table 2). SI Fig. 4 shows the ancient southern Africans plotted against the hypothetical ranges for a pure C_3 terrestrial diet, a pure C_4 terrestrial diet and a pure marine diet, together with the ranges for foraging and farming groups presented in SI Table 2.

The isotopic data from our individuals predating 2 kya, which includes all individuals from Matjes River and two individuals from Great Brak River and Cape St Francis, have relatively high δ^{13}C values (ranging from -16.5 to –12.9‰) and also high δ^{15}N values (ranging from 12.8 to 17.4‰). These values are consistent with what can be expected of hunter-gatherer/forager diets where marine resources (to varying degrees) and meat from terrestrial

Supplementary Information Table 2: Comparative isotope data for several groups of farmers, foragers and one group of pastoralists from sub-Saharan African contexts.

Population	δ^{13}C				δ^{15}N				Source
	Mean	SD	Range	Median	Mean	SD	Range	Median	
Pastoralist diet, Kenya (n=10)	-7.3	0.8	-8.1/-6.5	-5.9	12.6	0.8	13.4/11.8	12.8	(Ambrose & DeNiro, 1986)
Farmers, sub-escarpement savanna, SA farmer (n=7)	-10.6	2.4	-12.6/-8.2	-10.4	10.1	2.1	12.2/8.0	10.1	(Ribot *et al.*, 2010)
Farmers, western mesic grassland, SA (n=11)	-7.5	1.8	-9.3/-5.7	-7.0	10.3	0.9	11.2/9.4	10.4	(Lee-Thorp *et al.*, 1993)
Foragers inland SA (n=15)	-14.3	3.7	-10.6/-18.0	-13.8	14.4	2.5	11.9/16.9	13.5	(Ribot *et al.*, 2010)
Foragers Fynbos SA (n=6)	-17.5	1.2	-18.7/-16.3	-17.7	13.0	2.5	15.5/10.5	13.4	(Sealy *et al.*, 2000)
Foragers Cape Coastal (n=34)	-12.8	1.4	-14.2/-11.4	-12.5	14.9	1.6	16.5/13.3	15.3	(Lewis & Sealy, 2018)

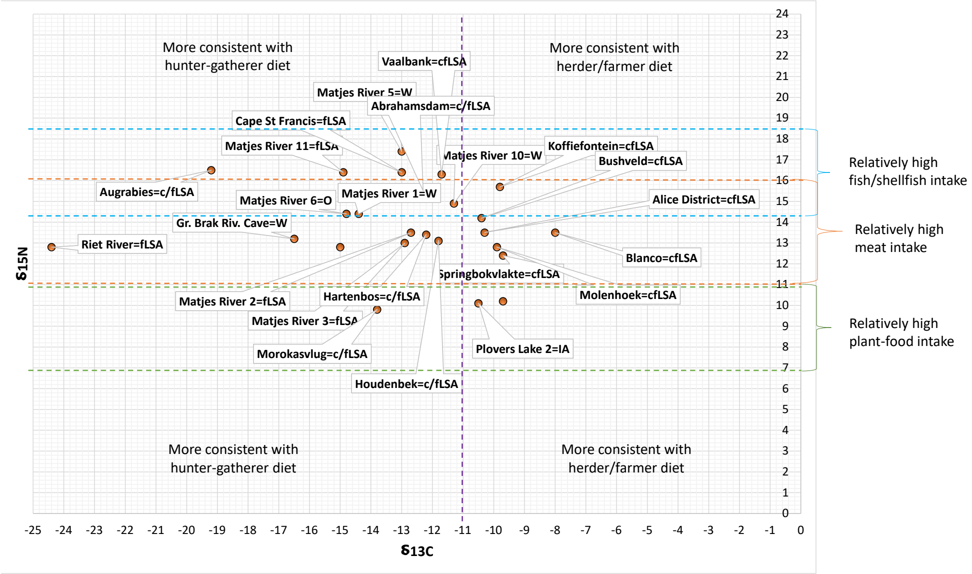

Supplementary Information Figure 4: Isotope values for individuals from ancient southern Africans included in this study plotted against general dietary blocks derived from Mays (1988) and supplementary table 2.

animals are consumed (Sealy, 2006; Lewis & Sealy, 2018). Individuals post-dating 2 kya display much more varied signals, where some broadly sharing

the previous hunter-gatherer/forager signals and others, such as Riet River and Blanco, have extreme terrestrial C_3 and C_4-signals, with $\delta^{13}C$ values of -24.4‰ and -8.0‰, respectively. Three individuals, Plovers Lake 1 and 2 (Lombard *et al.*, 2019) and Morokasvlug, shared some of the highest $\delta^{13}C$ values (ranging from -13.8 to -9.7‰) and the lowest $\delta 15N$ values (ranging from 9.8 to 10.2‰) and indicate a diet associated with farmers who consumed an entirely terrestrial diet which included C_4-plants and meat from C_4-grazers (Ribot *et al.*, 2010).

3.2 Mitochondrial haplogroups

A vast majority, 25 of 28 individuals, carried different variants of the mitochondrial haplogroup L0d (which is common among with current-day Khoe-San populations, (Schlebusch *et al.*, 2013; Barbieri *et al.*, 2016)). The individual from Alice District (flo019, dated to 330-505 cal. BP) carried an L0g haplogroup. Two (out of three) individuals from Tobias Cave carried haplogroup L0a.

3.3 Y-chromosome haplogroups

Y-chromosomal haplogroups could be confidently inferred for five out of eight males (Extended Data Table 1, SI Table 3). The oldest individual, Matjes River 6 (flo006) (10,255-10,155 cal. BP) shares the same haplogroup as Great Brak River Cave (flo014) and Cape St Francis (flo018) (2,100-2,355 cal. BP) A1b1b2a, which has previously been found among Khoe-San groups (Underhill *et al.*, 2000; Naidoo *et al.*, 2010; Batini *et al.*, 2011; Barbieri *et al.*, 2016; Naidoo *et al.*, 2020). Individuals Hartenbos (flo015) and Abrahamsdam (flo033) are younger, dating to 75-505 cal. BP. Both carried haplogroup E1b1b1, which has been found both in modern-day eastern African, western African and Khoe-San populations (Trombetta *et al.*, 2015), and has been associated with the migrations of eastern African pastoralists into southern Africa (Henn *et al.*, 2008). Individuals Augrabies (flo032), Plovers Lake 1 (plo001), and Plovers Lake 2 (plo002) did not have sufficient coverage to infer Y-chromosomal haplotype.

Supplementary Information Table 3: Y-chromosomal haplotype assignments.

Sample name	Y-chromosomal haplogroup	SNPs with derived state
flo006	A1b1b2a	L1120, L1130, L1135, M229, Z11918, Z17896, L1053, Z1013, Z11907, Z11899, V221, L985, V238, F3643, PF2276, Y6224, V156, V56, Y2986, BY451, Y4010
flo014	A1b1b2a	L1116, L1120, L1130, L1135, L1098, M229, Z11918, Z17896, L1053,L1013, Z11892, Z11899, V221, L985, V238, M9410, M5798, PF2276, V156, V56, Y2986, BY451, Y4010
flo015	E1b1b1	L1116, Z17896, L1013, M9081, M9228, M9262, M9295, PF961, PF210, M5607, PF1442, PF2276, Z837, PF2009, PF1635, PF1716, PF1882, FGC6230
flo018	A1b1b2a	L1116, L1120, L1130, L1135, L1098, M229, Z11918, Z17896, L1053, L1013, Z11892, Z11907, Z11899, V221, L985, V238, F3643, PF2276, Y6224, V156, V56, Y2986, BY451, Y4010, L348.3
flo033	E1b1b1b2	L1098, Z17896, M9124, M9343, Z17365, M11760, Z17710, PF821, PF1046, M5649, M5609, Z837, PF1724, PF1871, PF1894, PF1901, FGC6230

3.4 Variants associated with traits

Variants associated with potentially interesting traits, including infectious disease resistance were called according to (Schlebusch *et al.*, 2017). Supplementary Data 6 shows the genotypes of the six complete genomes of ancient southern Africans. The genotypes of the Ballito Bay A boy can be found in Table S25 in Schlebusch *et al.* (2017).

3.5 How far back in time does the ancient southern African ancestry extend?

From investigating the genomes of the ancient southern Africans together with radiocarbon dates going back to 10,200 years BP, we can concretely demonstrate that the ancient southern African ancestry extend towards the Holocene-Pleistocene transition. How far back in time did the distinct genepool exist? Population divergences (Sjödin *et al.*, 2021) between individuals representing the ancient southern African group (in total seven individuals with >7x genome coverage) to any other individual (representing ancient

western, eastern, and northern Africans, ancient non-Africans, modern-day non-Africans and modern-day western, eastern, and central Africans) were estimated to between ∼240,000 and 310,000 years ago (Supplementary Data 15-31). While this population divergence does not exclude small levels of gene-flow from eastern, central or western Africans, we note that the genetic affinity between ancient southern Africans and ancient (and modern-day) eastern Africans is similar to the genetic affinity between ancient southern Africans and ancient (and modern-day) western Africans (e.g. F_4(Denisova, Matjes River 1, Mota, ShumLaka)∼0, SI Fig. 5, main text Fig. 2B; see also Extended Data Table 1 for labIDs of ancient southern Africans). If there was long-term gene-flow (prior to 1300 years ago) between eastern Africa and southern Africa, it would have manifested in a stronger genetic affinity between these two regions in comparison with other regions (e.g. western Africa). To further investigate potential gene-flow, we contrast genetic affinity of ancient southern Africans to other African groups using an F_4-test of the form F_4(D,aSA,aEA,aWA). This test-statistic would be negative if there is gene-flow between ancient southern Africans (aSA) and ancient eastern Africans (aEA), or positive if there is gene-flow between ancient southern Africans and ancient western Africans (aWA). Since our test set-up hinges on diversification of western, eastern (and central) African groups, we note that we can only detect gene-flow after the diversification of these groups, which was estimated to 210,000-140,000 (Supplementary Data 25, 26, and 28) (Hollfelder *et al.*, 2021; Schlebusch *et al.*, 2020; Sjödin *et al.*, 2021). We note that ancient southern Africans that lived >1300 years BP show no greater affinity to ancient western Africans than to ancient eastern Africans, e.g. F_4(D,Matjes River 1, Mota, ShumLaka)∼0 (SI Fig. 5). We further explore a number of combinations of eastern, central or western Africans as potential sources for gene-flow with the ancient southern Africans, and we only find clear indications of gene-flow to (ancient) south-eastern Africa (Malawi) and the modern-day eastern African population isolate of the forager Hadza, which have previously been attributed to a larger geographic extent of the ancient southern African ancestry (Skoglund *et al.*, 2017). We further note an indication for some comparisons involving modern-day western Africans (Yoruba and Mandenka) to show negative values, which would be consistent with modest levels of archaic admixture into these groups (Skoglund *et al.*, 2017; Schlebusch *et al.*, 2017; Durvasula & Sankararaman, 2020; Wall *et al.*, 2009). In order to assess power to detect the gene-flow into ancient southern Africans, we replace Matjes River 1 by Hartenbos, Vaalkrans, and Kasteel-

berg, individuals with known eastern African admixture from a source similar to the Amhara (∼25% eastern African ancestry and the remaining fraction being ancient southern African ancestry (SI Fig. 5). If we use Amhara as the source population of the eastern African admixture, the admixture signal in Hartenbos, Vaalkrans, and Kasteelberg is clear, also for other individuals with less admixture and from other sources (Extended Data Fig. 6B). If we instead use Mota as a source population (which is genetically distinct from Amhara, see e.g. main text Fig. 2C), there is a trend of more negative values for this test (when contrasting Mota and ShumLaka, SI Fig. 5), but the test is only statistically significant for one of three comparisons (the one involving Vaalkrans), showing that if we have a poor genetic resemblance between the true source population and the proxy in an f_4 test, power to detect admixture decreases.

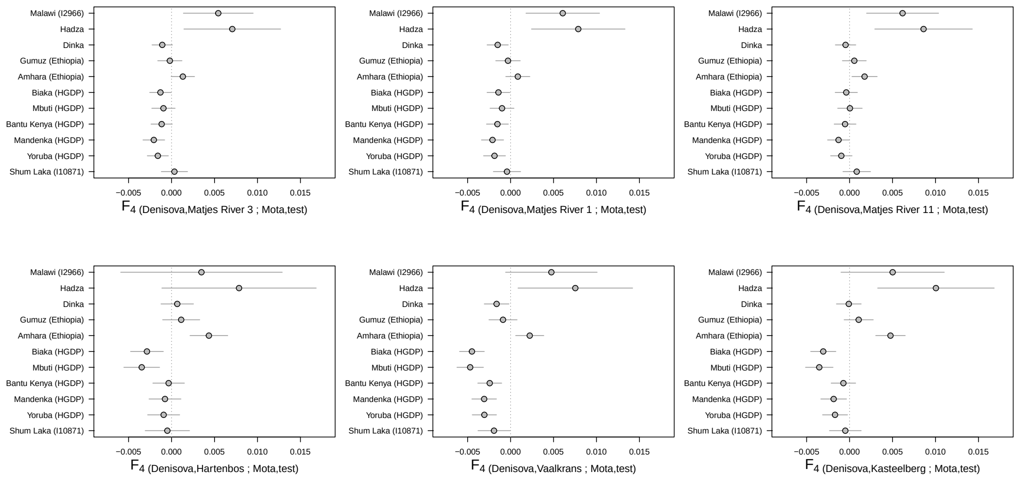

Supplementary Information Figure 5: F_4 test for gene-flow from ancient eastern or ancient western Africa into ancient southern Africa. Error bars indicate 95% confidence interval (CI).

Hence, in summary, we found no evidence of modest to large levels of gene-flow from western, eastern or central African regions *into* southern Africa in the last ∼150,000 years; it is only in the last 1,300 years that gene-flow *into* southern Africa is detectable. We note here that we can only detect and exclude gene-flow from modern-day and Holocene western, eastern or central African populations. Gene-flow from unsampled and/or unknown groups

cannot be assessed with f-statistics. Gene-flow *out* from southern Africa to southeastern Africa (Malawi and Zambia), can be detected as early as 8,000 years ago. These observations are compatible with gene-flow going *out* from southern Africa, potentially as pulses during favorable climatic conditions (Lahr & Foley, 1998), and it happened at least during the early Holocene to reach latitude 10°South (Skoglund *et al.*, 2017; Lipson *et al.*, 2022).

3.6 Recent gene-flow from eastern and western African groups

Among the individuals that lived more recent than 1300 cal BP, some stand out. For instance, individuals Blanco (flo022), Houdenbek (flo031), Augrabies (flo032), Hartenbos (flo015), Alice District (flo019) and Koffiefontein (flo027) fall outside the core ancient southern African cluster, close to between modern-day Khoe-San individuals and modern-day eastern African individuals in a plot of PC1 vs PC2 (main text Fig. 2A, Extended Data Fig. 1). All of these individuals, with the exception of Alice District (flo019), carry the Khoe-San associated mtDNA haplogroup L0d, and it is interesting to note that all these individuals were dated to between 0-720 calBP. These ancient individuals also show components of eastern African ancestry in an analysis of ancestry components, likely reflecting a level of eastern African migration of pastoralists followed by admixture, which appear first at 1300 calBP in our data (the date of the oldest of these admixed ancient southern Africans, Kasteelberg, main text Fig. 2C, Extended Data Fig. 2, SI Fig. 6). The estimated eastern African ancestry component based on ADMIXTURE (Alexander *et al.*, 2009), largely coincide with estimates based on f_4-ratios (SI Table 4).

Interestingly, individual Plovers Lake 1 (plo001) from Plovers Lake, once thought to possibly represent an individual who lived in the Pleistocene (which highlights the importance of directly radiocarbon-dating any human remain to be investigated), is in fact genetically similar to modern-day eastern African individuals, clustering together with published ancient pastoralists from Tanzania and Kenya (main text Fig. 2, Extended Data Figs. 1-2). The fact that this individual dates to $\sim$500 calBP and carries an L3d mitochondrial haplotype (Lombard *et al.*, 2019), common for eastern Africa, suggests the possibility that this is a first-generation migrant with eastern African pastoralist ancestry.

Another individual from Plovers Lake, Plovers Lake 2 (plo002), clusters closely to West African individuals and ancient Iron Age individuals dating to 500-300 calBP from sites in KwaZulu Natal (Eland Cave, Newcastle, Mfongosi and Champagne Castle, main text Fig. 2, Extended Data Figs. 1-2). This individual carries an L3d mitochondrial haplotype, and a substantial western African ancestry (main text Fig. 2C, SI Fig. 6).

Tobias Cave 1 woman (tob001), who carries a L0a mitochondrial haplotype common in southeastern African populations, is an unusual outlier. On the PCoA (main text Fig. 2A, Extended Data Fig. 1), she is placed between modern-day European, modern-day eastern African and modern-day Khoe-San individuals. Estimated ancestry fractions indicate a significant fraction of its ancestry clusters with a component maximized in Europeans (for a wide range of assumed number of ancestry components, SI Fig. 6). Although this genome is low coverage (0.02x), there is no evidence of significant contamination (Extended Data Table 1). The high level of apparent European-associated ancestry in this individual is surprising given the age of the site (radiocarbon dated to ∼600 BP). However we note that as for all individuals from Tobias Cave, there are only indirectly radiocarbon dates from associated material, and it is possible that the human remains are from a more recent date.

Supplementary Information Table 4: Estimated eastern African admixture using an f_4-ratio test of $f4$(A, O; X, C)/f4(A, O; B, C) where A=Dinka, B=Amhara, C=Ballito Bay A, O=Denisovan, and X=ancient individual tested.

Ancient individual	East African admixture proportion	SE	Z-score
flo015	0.502	0.060	8.304
flo032	0.409	0.070	5.803
flo027	0.531	0.092	5.771
flo033	0.188	0.078	2.416
plo001	1.234	0.202	6.124
plo002	0.788	0.137	5.763
tob005	0.071	0.051	1.381
flo031	0.208	0.090	2.325
flo019	0.257	0.200	1.287

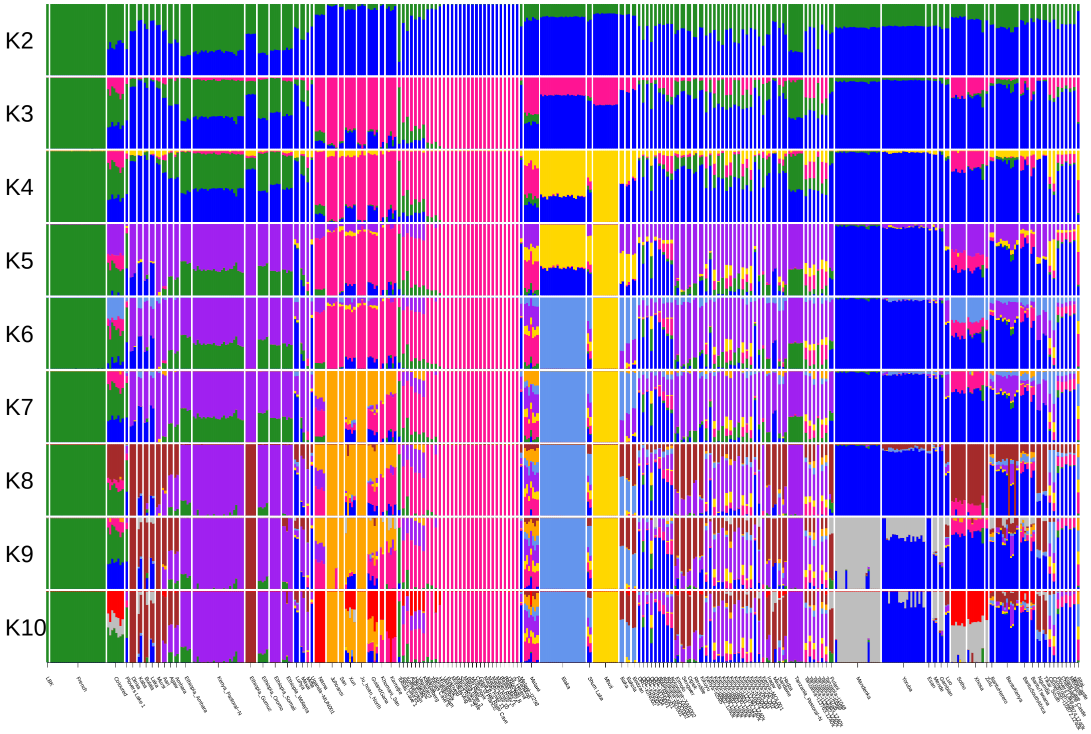

Supplementary Information Figure 6: ADMIXTURE analyses showing results for K=2-10, 25 iterations per K, for all analyzed individuals.

3.7 Population continuity at Matjes River

The strong clustering of ancient southern African individuals who lived between 10,200 and 1,400 years BP, suggests a continuous ancient population existing across southern Africa throughout the Holocene. To further explore this genetic continuity through time, we applied the "anchor test" (McKenna *et al.*, 2024), which uses the fraction of shared derived variants as a measure of population continuity, to investigate population continuity. Using the oldest high-coverage genome of the ancient southern Africans, Matjes River 1 (∼7,800 calBP) as the anchor individual, we find a pattern of population continuity existing among individuals inhabiting southern Africa for a period of at least ∼7,000 years (Extended Data Fig. 3). The anchor statistic is unaffected forwards in time from the anchor population by the action of genetic drift, but decreases for individuals sampled from population split-

ting off prior to the anchor, or where an admixture event has occurred from such a diverged population. The anchor shows little difference among the ancient southern Africans before 1,300 BP, but after that point in time, several individuals show partial discontinuity. However, we note that for the majority of the investigated time, the individuals come from Matjes River or from sites close by, with a few exceptions (see main text Fig. 1B). Hence, we can only be certain of Holocene continuity for Matjes River. Finally, all other modern-day and ancient Africans (including ShumLaka and Mota) together with non-African populations share a similar anchor statistic, indicating a shared divergence event of all these populations with the ancestors of ancient southern African hunter-gatherers.

3.8 Kinship and stratification among ancient southern Africans

In order to assess relationships among ancient southern Africans, we plotted pairwise outgroup-f_3-values using the chimpanzee genome as outgroup displayed in SI Fig. 7. We first note closer relationships among Alice District (flo019), Blanco (flo022), and Molenhoek (flo025), and, to some degree, Augrabies (flo032), which all are low-coverage genomes. Overall, the clustering of individuals closely reflects the clustering of samples in both PCoA and ADMIXTURE results. There is a time-associated clustering of individuals, with more ancient individuals (10,200-1,000 calBP) sharing genetic affinity distinct from the more recent individuals (1,000-0 calBP). Therefore it seems that the only appreciable genetic differentiation among our individuals is likely caused by admixture within the past 1,000 years. Intriguingly, these ancient individuals showing homogeneous genetic affinities come from sites in the Eastern Cape, Western Cape, KwaZulu-Natal and Free State provinces of South Africa. None of the more recent, highly admixed individuals come from KwaZulu-Natal, instead they were excavated in the northern and central provinces of the Northern Cape, Limpopo and Free State.

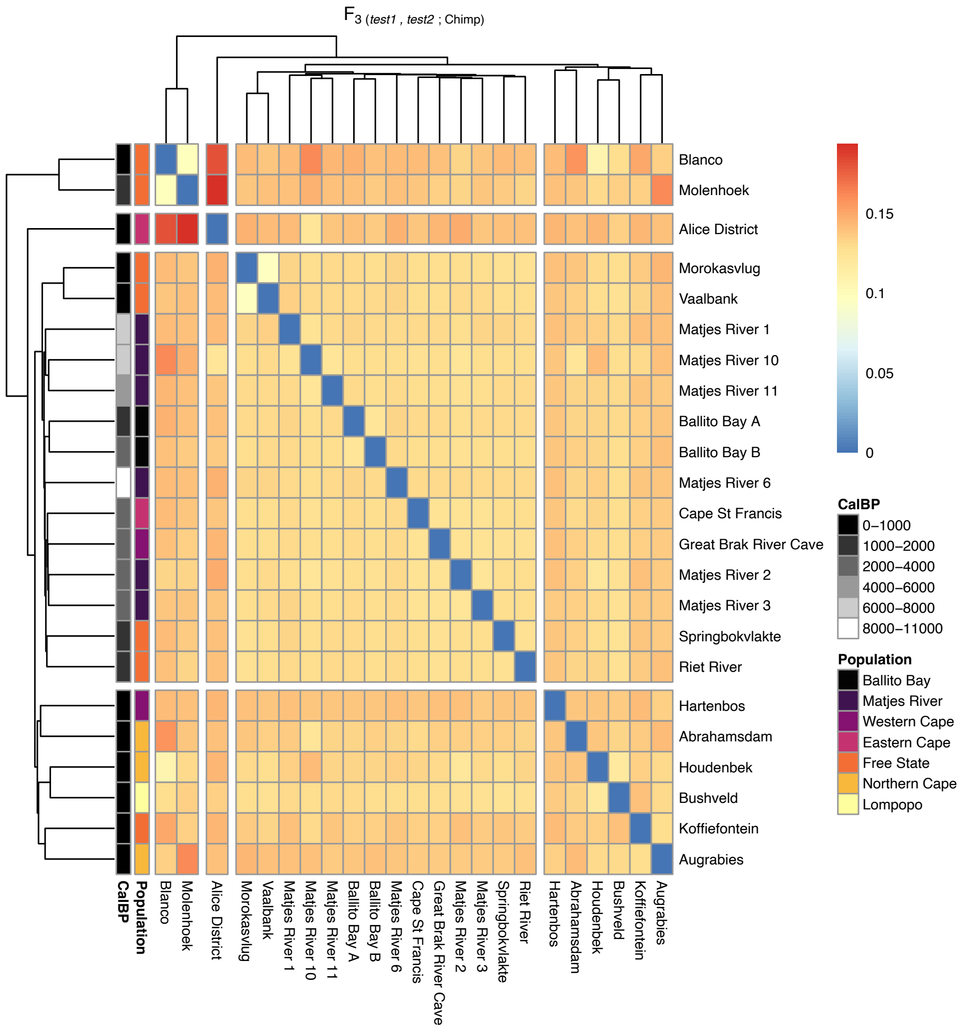

Supplementary Information Figure 7: A heatmap representing a pairwise dissimilarity matrix (Euclidean distance) of outgroup-f_3 values estimated for ancient southern African individuals. Warmer colors indicate higher levels of shared drift between individuals.

To test the hypothesis of an unstratified ancient population in South Africa before 1,300 years ago, we estimated the correlation between pairwise genetic distance and geographic distance and time (SI Fig. 8). All ancient individuals older than 1,300 years were included in the analysis (17 individuals and 136 pairwise comparisons in total). For these comparisons, there

was no indication of genetic stratification across time (r^2=0.02, p=0.06), and low levels of stratification across space (r^2=0.07, p=0.001) despite spanning ~9,000 years and a vast geographic area (SI Fig. 8).

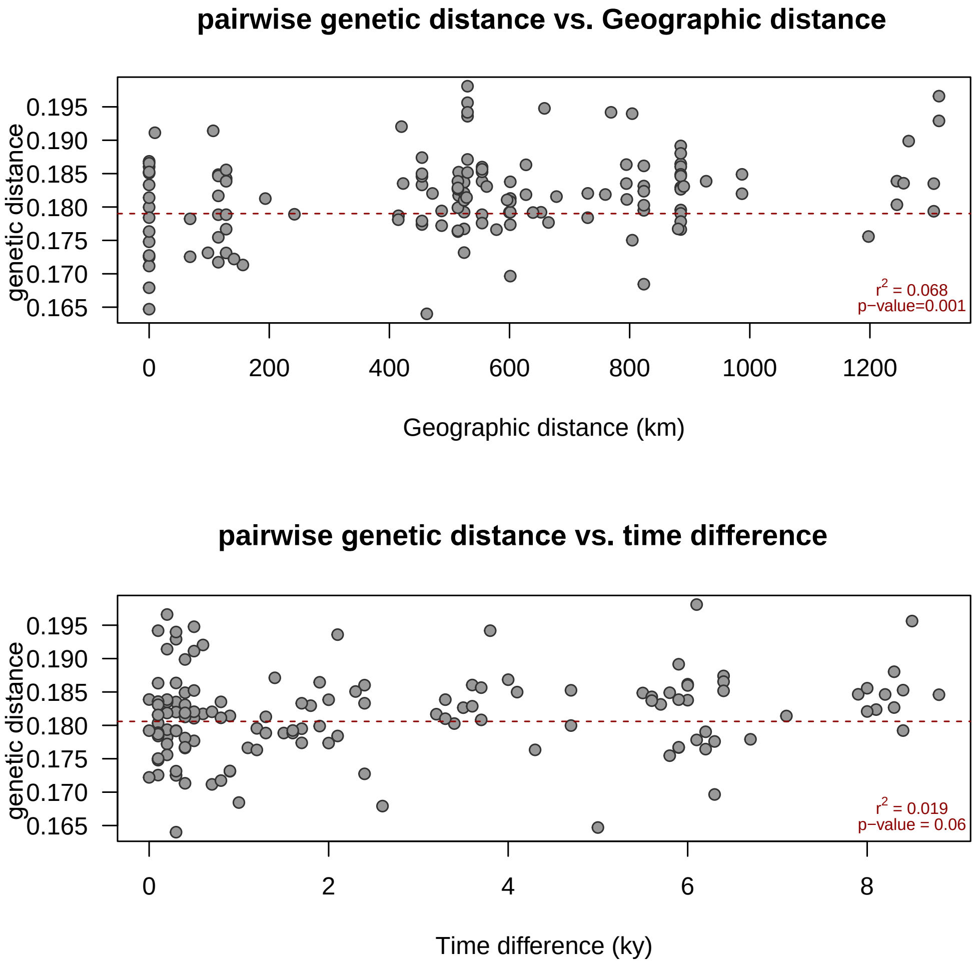

Supplementary Information Figure 8: Genetic distance between pairs of individuals compared to geographic distance and difference in time. Top panel shows correlation with geographic distance in kilometers (km) and bottom panel shows correlation through time in 1,000 years (ky). Dashed line (dark red) indicates correlation estimate from linear model.

3.9 Modern-day Khoe-San groups carry the greatest level of ancient southern African ancestry

In order to determine which modern Khoe-San group is genetically most similar to ancient southern Africans, an outgroup-f_3 test was used. Using Denisovan as an outgroup, Southern Khoe-San populations Karretjie people and Khomani-San show the closest genetic affinity (the most shared drift) with ancient southern African hunter-gatherers (SI Fig. 9). Interestingly, there is no significant correlation between outgroup-f_3 results and the dates of the ancient southern Africans (Karretjie people: Pearson correlation = -0.050, p-value = 0.814, and Khomani-San: Pearson correlation = 0.052, p-value = 0.805). In fact, in both cases the individual with the highest shared drift with modern-day Khoe-San groups is Matjes River 2 (flo002), dating to ~2,970 calBP.

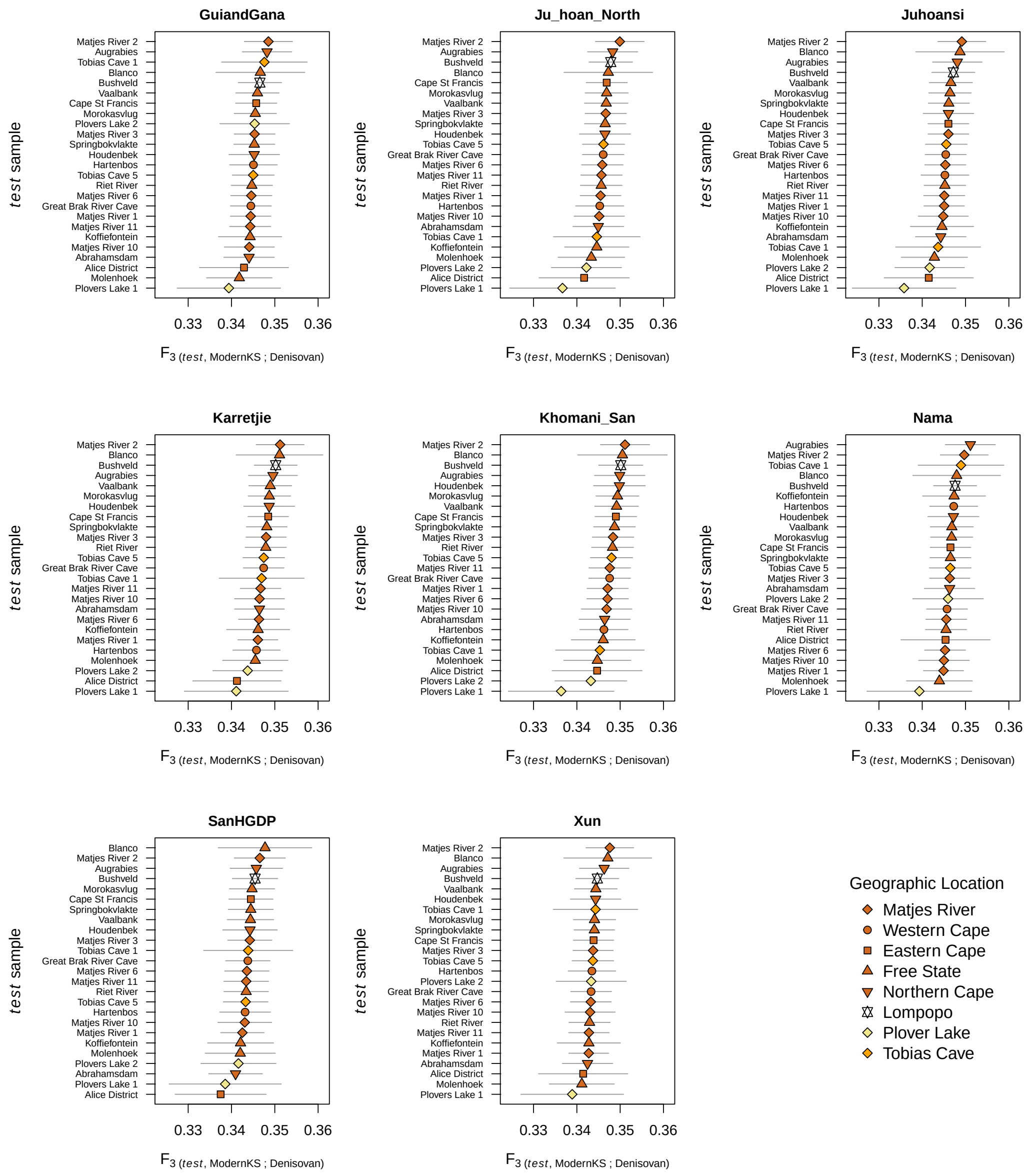

Supplementary Information Figure 9: Outgroup-f_3 statistics of form f_3(A, B; O) where A=ancient test sample, B=Modern Khoe-San population and O=outgroup (Denisovan). Error bars indicate 95% confidence interval (CI). Estimating shared drift between ancient southern African hunter-gatherers and modern Khoe-San groups, with results showing greatest shared drifts to southern San populations Khomani-San and Karretjie people.

We further note (e.g. assuming ≥7 ancestry components, Extended Data Fig. 2, SI Fig. 6) that the ancient southern African hunter-gatherer component separates from that of the northern Khoe-San populations. Although the northern Khoe-San population Ju|'hoansi seem the least admixed among the Khoe-San modern-day groups, it is the southern Khoe-San group Karretjie who share closest genetic affinity to the majority of the ancient southern Africans. The close affinity between the ancient southern African individuals and published Late Stone Age (∼2 kya) southern African hunter-gatherer individuals from Doonside, Ballito Bay, Faraoskop and St. Helena is again reflected here at all investigated number of clusters.

3.10 Past population sizes of ancient southern Africans

We used MSMC to estimate effective population sizes (N_e) through time using single diploid genomes from the highest coverage ancient southern African individual, Matjes River 11, and compare to the previously analysed genome of the Ballito Bay A boy (Schlebusch *et al.*, 2017). For plotting results we assume a generation time of 29 years and mutation rate of 1.45×10^{-8}. We compare the ancient southern African trajectories to genomes from modern day groups (including Khoe-San and Bantu-speakers from southern Africa, see Fig. 4A in Schlebusch *et al.* (2020), and ancient northwestern Africans, see Fig. 2 in Simões *et al.* (2023)). All groups were inferred to have similar N_e of ∼30,000 around 200 kya (SI Fig. 10), followed by a slow decline in N_e backwards in time. While all groups begin to experience a decrease in N_e around 100 kya, this reduction in N_e is stronger for all ancient individuals than the modern-day Khoe-San (Schlebusch *et al.*, 2020). The reduction in N_e for ancient southern Africans has been shown previously (Schlebusch *et al.*, 2020), and the fact that the ancient southern Africans show similarly strong reductions in N_e suggest that there really was a reduction in population size among all (yet investigated) groups across the globe. The relatively modest reductions in N_e among modern-day Khoe-San groups is an artefact of recent admixture (<2 kya), which can veil true changes in census population size by increasing genetic diversity (hence N_e) (Mazet *et al.*, 2016).

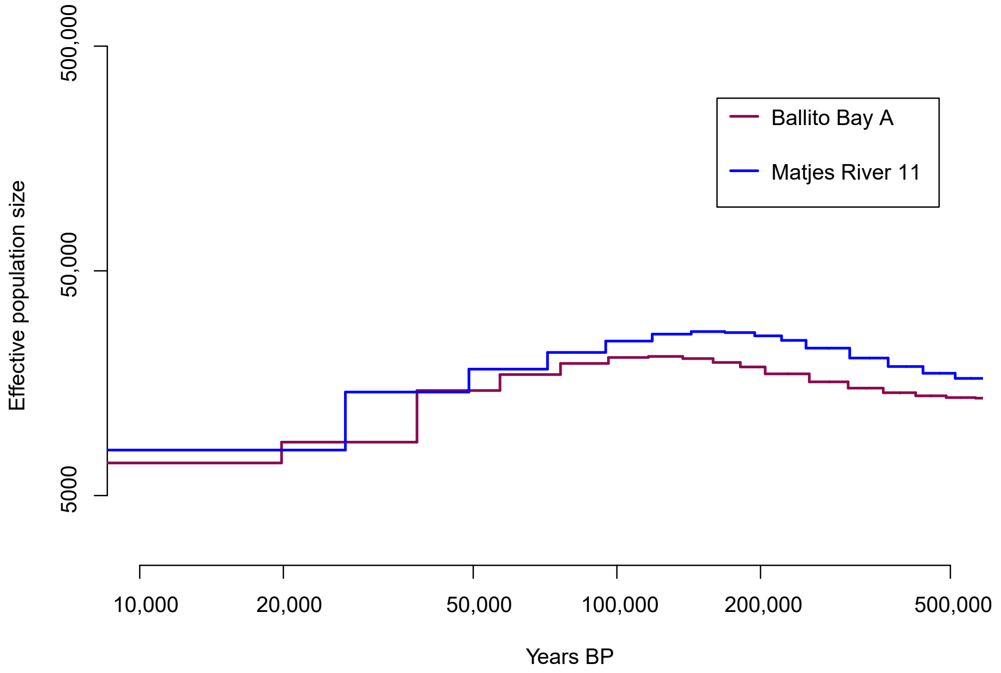

Supplementary Information Figure 10: Trajectories of effective population size through time for ancient and modern African populations estimated using MSMC. Assumed mutation rate (μ) of 1.45×10^{-8} and generation time (g) of 29 years.

3.11 Population divergence times

Using seven complete (>7x) genomes from ancient southern Africans, we investigated population divergence times (Sjödin *et al.*, 2021) between ancient southern African hunter-gatherers and comparative ancient and modern-day individuals.

Population divergences were estimated to ~285-240 kya between ancient southern Africans on the one hand and ancient northern, eastern, and western Africans on the other (Supplementary Data 24, 27, and 29). These estimates were similar to comparisons with modern-day eastern, central, western Africans as well as non-Africans (~310-260 kya, Supplementary Data 20, 27,

29, and 30). These estimates recapitulate findings that the divergence between the southern African hunter-gatherer population and all non Khoe-San populations captures the deepest divergences among modern humans (Schlebusch *et al.*, 2017, 2020). The possibility of using genomes from individuals that predate recent large-scale movements of people in Africa associated with food production and colonialism, allows us to avoid effects of recent admixture among modern-day populations that impact many analyses of population history, including reducing divergence time estimates (Schlebusch *et al.*, 2017, 2020; Sjödin *et al.*, 2021).

The average population split-time estimates between ancient southern African individuals and modern-day Khoe-San groups range from ∼96 kya to ∼147 kya (averages across the seven comparisons involving ancient southern Africans), with Karretjie people and Khomani representing the most recent splits, followed by the Northern Khoe-San populations including Xun, Nama and Ju|'hoansi (Supplementary Data 31). We note however that due to the recent admixture into modern-day Khoe-San groups, a clean split model will estimate a divergence time between the two ancestries in the modern-day Khoe-San (SI Fig. 11).

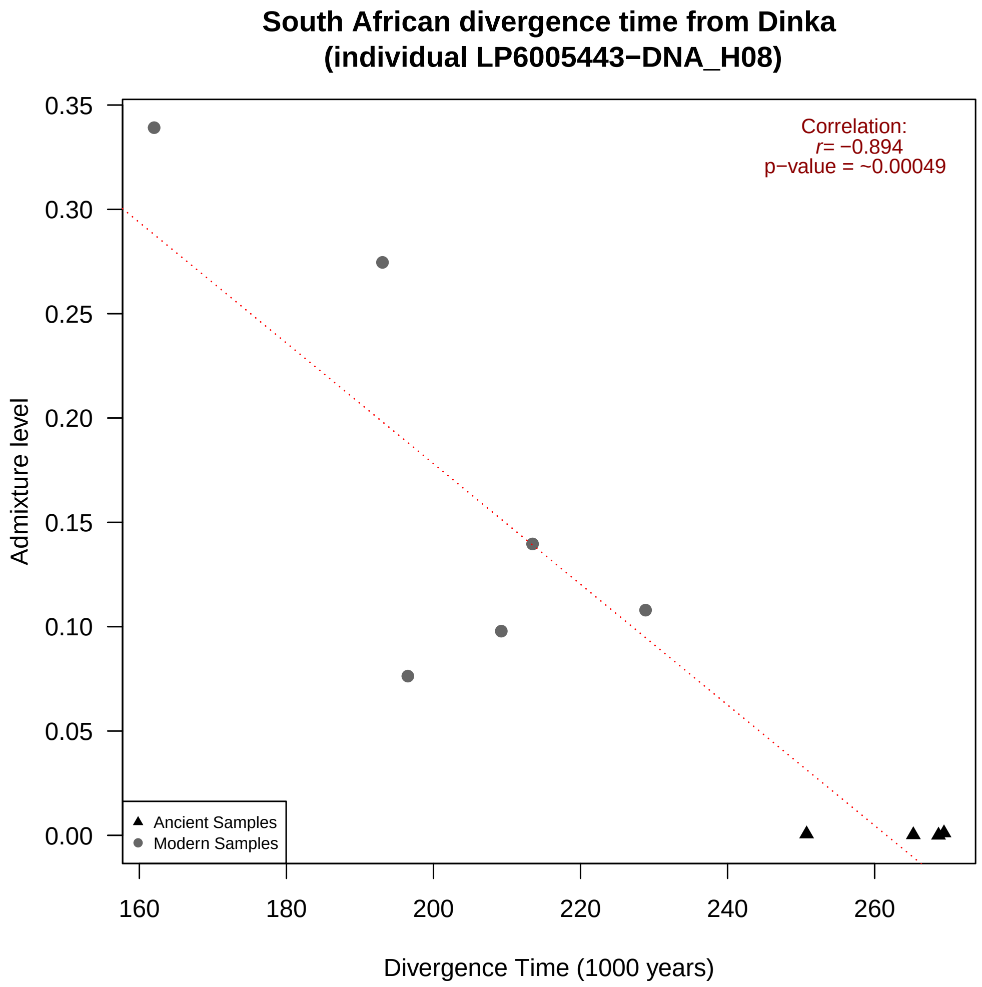

Supplementary Information Figure 11: Average population divergence times between eastern African Dinka (x-axis) and 5 modern-day Khoe-San groups (Schlebusch *et al.*, 2020) with varying eastern African admixture (y-axis), as well as for 4 ancient southern Africans (with >10 genome coverage). Dashed line (dark red) indicates correlation estimate from linear model.

Indications of deep gene-trees, particularly in West African populations, has led to investigations of archaic hominin introgression into African populations, including from unsampled "ghost" populations (Durvasula & Sankararaman, 2020; Lipson *et al.*, 2020) or back-to-Africa migrations from Eurasia

(Bergström *et al.*, 2020; Chen *et al.*, 2020; Wang *et al.*, 2013; Cole *et al.*, 2020). An alternative possibility is a complex demography of deep and long-lasting ancestral stratification within human populations in Africa (Scerri *et al.*, 2018; Ragsdale *et al.*, 2023; Fan *et al.*, 2023), though discriminating between models of archaic introgression and deep stratification is difficult (Hollfelder *et al.*, 2021). Although direct comparisons of complete genomes (main text Fig. 2D) does not rule out complex demographic histories, nor low levels of archaic admixture, the deep human history in Africa can nevertheless be represented by deep stratification – possibly isolation – between southern Africa on the one hand and western/central/eastern Africa and the rest of the world on the other hand.

It has been hypothesized that deep ancestral stratification (e.g. in Africa) may upwardly bias estimates of population divergences using methods that rely on the assumption of a clean-split model, such as the TTo method (Sjödin *et al.*, 2021). To test the robustness of estimates of population divergence in the scenario of deep ancestral stratification, we conducted a simulation study. Simulations were performed using msprime under two demographic models (SI Fig. 12) and the simulation parameters are given in SI Table 5. We simulate a total of 500 genome regions of 5 Mb size to mimic a complete human genome sequence, with mutation rate of 1.45×10^{-8} and recombination rate of 1.2×10^{-8}. The generation time is assumed to be 29 years. The "No Ancestral Stratification" model includes a population divergence event at 300,000 years ago. The "Ancestral Stratification" model includes the same population divergence event at 300,000 years ago, with an additional period in which the ancestral population splits into two isolated populations for 200,000 years, representing deep ancestral population stratification. 100 replicate simulations were performed under each model. Based on 100 simulations for each model and for two populations, with one diploid genome drawn from each population, we estimate population divergence time using the TTo approach (Sjödin *et al.*, 2021). The 100 estimated population divergences for each model are plotted in SI Fig. 13. For the "No Ancestral Stratification" model, the population divergence is tightly estimated around 300,000 years as expected. For the "Ancestral Stratification" model, the population divergence is estimated slightly below 300,000 years, demonstrating that the TTo approach is mildly downward biased in the case of deep ancestral structure (and not significantly upwardly biased). The intuition behind this robustness comes from the fact that the TTo method ascertains sites that are polymor-

phic in an outgroup (e.g. Neandertals or Denisovans) when calculating key parameters of the split model. Since the derived alleles at those sites arose prior to the divergence of the outgroup, they are not impacted by subsequent population stratification events occurring in the population directly ancestral to the test populations involved in the divergence event under study.

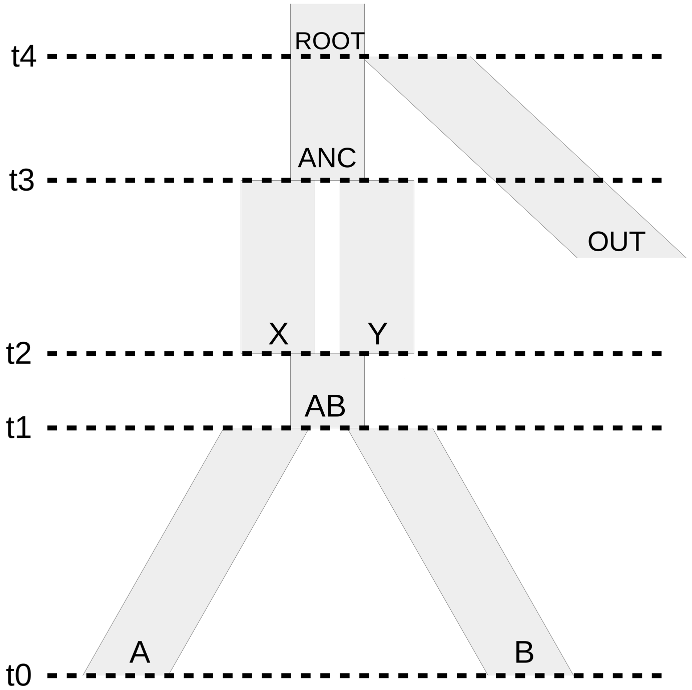

Supplementary Information Figure 12: A diagram showing the general structure of the demographic models simulated to test the effects of deep ancestral stratification on divergence time estimation. Looking forwards in time, a ROOT population splits into two populations ANC and OUT at time t4. OUT is sampled and goes extinct at 40,000 years. ANC splits into populations X and Y at time t3, which remain isolated until time t2, at which point they merge to form ancestral population AB. AB splits at time t1 to form populations A and B, remaining isolated until being sampled at time t0.

Supplementary Information Table 5: Parameters used in msprime simulations of demographic models involving "No Ancestral Stratification" and "Ancestral Stratification". The general structure of the split models simulated is shown in SI Fig. 12. The code for running simulations is available at https://github.com/jammc313/Ancestral-Stratification.git

Simulation parameter	No Ancestral Stratification	Ancestral Stratification
Ne (all populations)	10,000	
Replicates	100	
Mutation rate	1.45e-8	
Recombination rate	1.2e-8	
Generation time (years)	29	
Outgroup sampling (years)	40,000	
t0 (years)	0	
t1 (years)	300,000	
t2 (years)	350,000	
t3 (years)	350,001	550,000
t4 (years)	600,000	

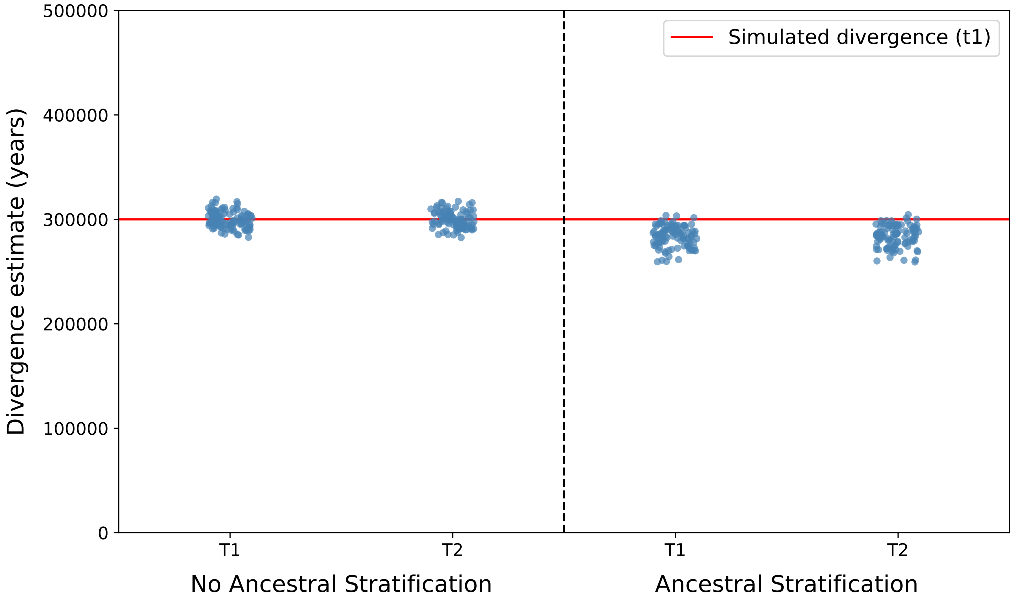

Supplementary Information Figure 13: Results of TTo divergence time estimation in simulated models of "No Ancestral Stratification" and "Ancestral Stratification". The general demographic model structure is shown in SI Fig. 12 and the parameters used in the simulations are listed in SI Table 5. Two split times (T1 and T2) are estimated, one for each branch of the population split model. One hundred replicate simulations are performed under each model, and the resulting mean estimates plotted against the true simulated split time of 300,000 years.

3.12 *Sapiens*-specific variants

Based on archaeological, paleoanthropological and genomic data, we have gained a coarse-grained understanding of the demographic history as *H. sapiens* emerge and diverge from archaic humans, but our understanding of the genetic architecture of the traits involved in that evolutionary process remains limited. Recent large-scale Genome-Wide Association studies have tended towards describing adaptation of (quantitative) traits as a collective effect caused by small shifts in the frequencies of a large number of underlying (small-effects) genetic variants (polygenic adaptation) (Boyle *et al.*, 2017; Lappalainen *et al.*, 2024). The more traditional population-genetic view of selective sweeps entails a small number of favourable variants which sweep towards fixation. These two descriptions are likely the extremes of a contin-

uous landscape of genetic architecture designs involving few gene-variants of larger effects to many gene-variants with small effects. How these descriptions of genetic architecture fit with human evolution is still unknown. Here, we will investigate all *sapiens*-specific variants across the genome and contrast that to *sapiens*-specific variants at amino-acid-altering sites, where the latter category represent variants that alter the protein sequence and may have functional impact.

To assess genetic variants that are unique to *H. sapiens* – variants that arose somewhere on the branch leading to *H. sapiens* or on an internal branch among humans (shown as thick black lines in main text Fig. 2D), we extracted all variable sites across the complete genomes (>7.2x coverage) of the 7 ancient southern Africans, 3 ancient Africans from the east (n = 1), west (n = 1) and north (n = 1), 7 pre-Neolithic Eurasians, 7 Northern San (Ju|'hoansi), 5 Southern San (Karretjie people), 208 individuals (8 individuals each from 26 populations) from the 1KG project, and four archaic humans (3 Neandertals and 1 Denisovan). We coin the term '*sapiens*-specific' variants for derived variants in the set of *H. sapiens* where the four archaic humans were fixed for the ancestral variant.

A striking 50.4% (10,556) of the *sapiens*-specific amino-acid-altering variants were unique to the ancient southern Africans (in total 20,956 such sites were variable among the aSAs) compared to a representative set of 208 individuals from the 1KG project (main text Fig. 4B). In contrast, only 15.5% (2,243 of 14,489) *sapiens*-specific amino-acid-altering variants were unique among pre-Neolithic Eurasians compared to the same set of individuals (main text Fig. 4B). Replacing the ancient southern Africans with northern San (SI Fig. 14) or southern San (SI Fig. 15) gives similar numbers, but the sets of variants from the modern-day San groups and the ancient southern Africans were only partly overlapping (main text Fig. 4C, Extended Data Fig. 8), demonstrating that these three groups carry large amounts of private *sapiens*-specific amino-acid-altering.

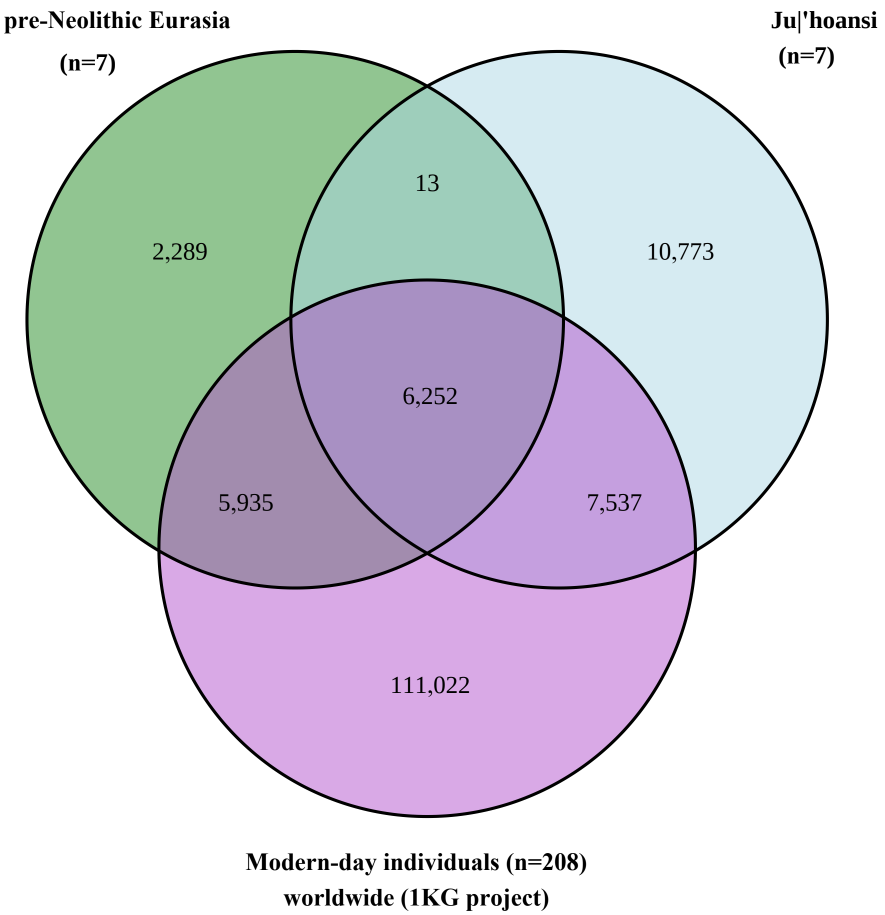

Supplementary Information Figure 14: Venn-diagram of 143,821 *sapiens*-specific amino-acid-altering variants in 7 modern-day Northern San (Ju|'hoansi) and 7 pre-Neolithic Eurasians compared to 208 individuals from the 1KG project (see main text Fig. 4B for comparison.)

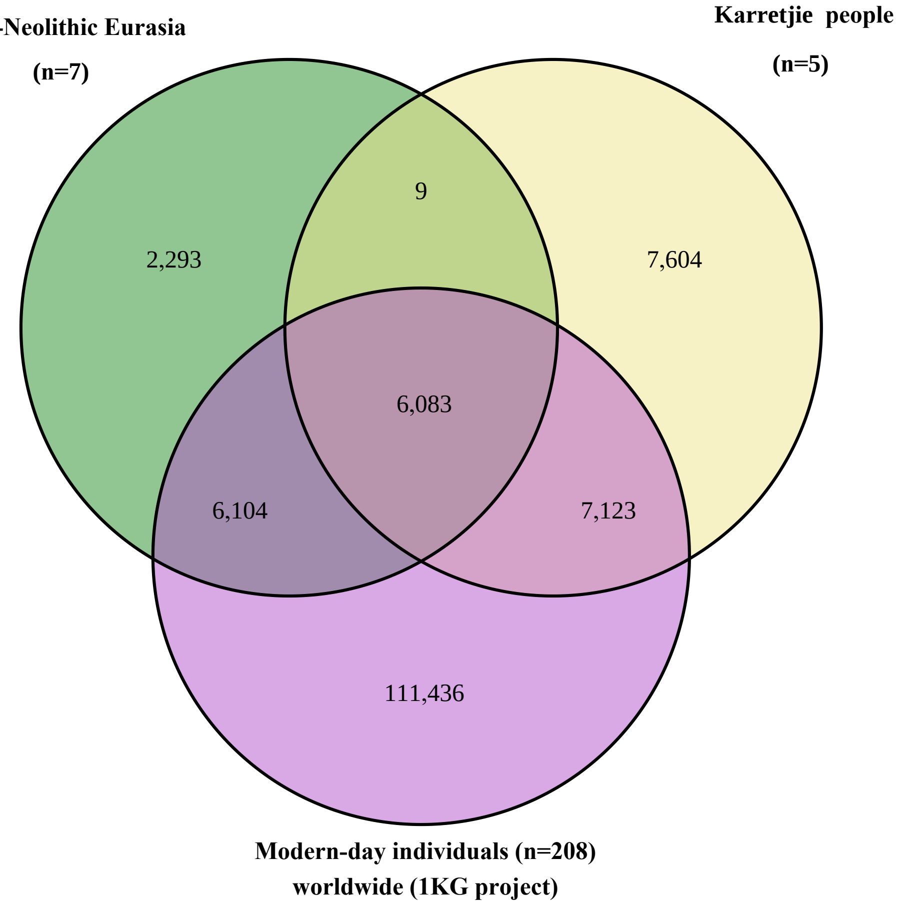

Supplementary Information Figure 15: Venn-diagram of 140,652 *sapiens*-specific amino-acid-altering variants in 7 modern-day Southern San (Karretjie People) and 7 pre-Neolithic Eurasians compared to 208 individuals from the 1KG project (see main text Fig. 4B for comparison.)

3.12.1 *Sapiens*-specific variants and kidney function

We found 87 *sapiens*-specific amino-acid-altering variants (located in 79 genes) fixed among all investigated individuals – including the ancient southern Africans. Among these, a remarkable enrichment and over-representation

of kidney function (14 of the top-15 gene ontology (GO) categories) was observed (Supplementary Data 12). 7 of the 87 *sapiens*-specific amino-acid-altering variants in 7 of these 79 genes (DCHS1, GREB1L, KIF26B, SCAP, SHANK2, SLC6A4, and ADSL) are connected to kidney associated GO-categories. This observation suggests a rapid adaption of kidney functions on the *sapiens* lineage, potentially connected to improved water-retention, a trait that is linked to humans compared to other great apes (Pontzer *et al.*, 2021). These 7 genes all have established functions and disease-associations connected to the kidney, although the genes have many additional associations to other traits and organs.

The *DCHS1* gene is crucial for kidney development, playing a role in the formation and patterning of nephrons through its interaction with the *FAT4* protein. Mutations in *DCHS1* can lead to kidney defects, including reduced size, cystic kidneys, and impaired nephron formation. This gene is involved in a signalling pathway that regulates the growth and branching of the ureteric bud and is important for the survival of renal tubular epithelial cells (Mao *et al.*, 2015). In the mouse, gene-targeted mutation analyses have shown that *DCHS1* cooperates with *FAT4* to regulate planar cell polarity and tissue morphogenesis not only in kidney, but also other organs including the ear, intestine, heart, lung, and skeleton (Mao *et al.*, 2011).

The *GREB1L* gene is a protein-coding gene crucial for early development, particularly of the kidneys, uterus, and inner ear. Mutations in *GREB1L* are linked to congenital anomalies of the kidneys and urinary tract like renal agenesis and other conditions such as certain types of deafness. It functions as a co-activator for retinoic acid receptors, which are critical for regulating gene cascades involved in development (Wu *et al.*, 2022).

The *KIF26B* gene is crucial for kidney development, and its disruption can lead to kidney agenesis due to impaired ureteric bud attraction. In adults, the gene has been linked to progressive kidney disease (renal fibrosis) and has been identified as a potential tumor promoter in bladder cancer. *KIF26B* regulates mesenchymal cell adhesion and microtubule association during development (Uchiyama *et al.*, 2010).

The *SCAP* gene in the kidney encodes a protein that regulates cholesterol and fatty acid homeostasis. In the kidney, this process helps manage lipid metabolism, and dysfunction of the *SCAP* pathway is implicated in kidney diseases such as diabetic nephropathy, where high cholesterol can worsen

kidney damage (Mukhi *et al.*, 2024).

The *SHANK2* gene is involved in kidney function, where it is expressed in epithelial cells of the renal proximal tubule and podocytes. It plays a crucial role in endocytosis, particularly in the reabsorption of albumin by podocytes and the retrieval of the sodium-phosphate co-transporter in proximal tubules. Dysfunction of the *SHANK2* gene is linked to increased risk and poor prognosis for renal cell carcinoma, as well as albuminuria in studies of mice (Chang *et al.*, 2022).

The *SLC6A4* gene encodes the serotonin transporter, which is involved in serotonin reuptake in the brain, but also locally synthesized in organs like the heart and kidney. While its primary role is in the nervous system, serotonin transport in the kidney plays a role in local serotonin signalling and function. Genetic variants in the *SLC6A4* gene may be linked to kidney diseases like chronic kidney disease and polycystic kidney disease (Hasan *et al.*, 2021).

The *ADSL* gene, and mutations in the gene, has been linked to a deficiency of purine metabolism, which affects the production of purine nucleotides. This deficiency can impact various organs, including the kidney, by impairing enzymatic activity and leading to the accumulation of toxic substances. Symptoms are wide-ranging and can include severe neurological issues and deficient enzyme activity in the kidney. While the most severe effects of *ADSL* deficiency are neurological, a kidney involvement can manifest as progressive loss of kidney function, similar to other genetic kidney diseases (Spiegel *et al.*, 2006).

3.12.2 *Sapiens*-specific variants enriched among the ancient southern Africans

By focusing on *sapiens*-specific amino-acid-altering variants fixed among the ancient southern Africans that display low frequencies in other human groups (i.e., high frequency of the ancestral variant), we can isolate local adaptations. Among the top-10 variants that showed the greatest difference between ancient southern Africans and the 1KG individuals, 3 variants were located in genes associated with UV-light protection, skin-diseases, and/or skin-pigmentation (Supplementary Data 14, Ijaz *et al.*, 2019; Engelken *et al.*, 2014; Hernandez-Pacheco *et al.*, 2017). Previous selection scans have also

found enrichment of UV-light protecting variants among modern-day Khoe-San (Schlebusch *et al.*, 2012) and we speculate that UV-light protection was important and adaptive in southern Africa due to arid, grassland and savanna ecologies offering limited natural protection from UV-light.

4 Supplementary discussion

4.1 Palaeoanthropological context

While our ancient genomic data support the interpretation of long-term isolation among southern African forager populations until at least ∼1.3 kya, we emphasize that this conclusion applies specifically to gene flow and not necessarily to cultural or economic interactions. That being said, there are several studies from other disciplines that commented on isolation or migration in southern Africa. For example, multiple lines of evidence suggest that contact between hunter-gatherers and incoming herders or farmers did occur during the last 1,000 years, particularly in the northern and eastern parts of South Africa (Forssman, 2023). Craniometric studies have also been used to argue for continuity across Later Stone Age (LSA) populations, regardless of subsistence strategy. Stynder (2009) found that LSA herders and hunter-gatherers from the southwestern and southern coastal regions exhibit only a modest increase in cranial variation following the introduction of herding – an increase suggested to be attributed to small-scale gene flow. Similarly, Stynder *et al.* (2007) demonstrated that early to mid-Holocene crania already displayed the craniofacial morphology characteristic of later Khoe-San populations. These data suggest a degree of morphological continuity across millennia. At the same time, localized boundaries and low mobility are inferred from isotope and archaeological studies along the southern and western coasts (Sealy, 2016; Lewis & Sealy, 2018), consistent with our observation of minimal gene flow into these regions before 1.3 kya. Taken together, the evidence supports a scenario in which forager populations in the far south remained genetically relatively isolated until the last millennium.

Craniometric analyses further suggests that this phenotype – defined by gracile cranial morphology, reduced facial prognathism, and other traits typical of recent Khoe-San populations – was already well established among Later Stone Age individuals during the mid- to late Holocene (Stynder, 2009; Stynder *et al.*, 2007). However, the ∼36,000-year-old Hofmeyr cranium from

the interior of southern Africa bears little resemblance to these Holocene individuals and instead shows close morphological affinities to Upper Paleolithic Europeans (Grine, 2023; Ribot *et al.*, 2023). This contrast implies that the emergence of the distinct Khoe-San phenotype likely postdates the Last Glacial Maximum (LGM), and may have been shaped by regional isolation during the late Pleistocene and early Holocene. Our findings of long-term population continuity in the far south of Africa during the Holocene are consistent with the idea that this region was a key area for human occupation. The deep population divergence of ancient southern Africans (as well as modern-day Khoe-San) to other African groups vastly surpasses the age of the Hofmeyr cranium, which in turn raises interesting questions about the genetic and morphometric diversity in southern Africa before the LGM.

4.2 On population stratification and large diversity

The observation of large genetic diversity among ancient southern Africans (main text Fig. 2D-E) at the same time as observing little stratification (explained by geographic distance or time) can appear as perplexing. For instance, the ancient southern Africans do have large genetic diversity, both as individuals (e.g. heterozygosity in main text Fig. 2E) and as a group (pairwise differences in main text Fig. 2D). One could expect that of this diversity appear as stratification, in that some individuals would be genetically more similar and form a group in contrast to other individuals. However, that is not what we observe (except for the individuals with admixture from non-southern African sources, SI Fig. 7). Further, by comparing pairwise genetic distance and cal BP difference, there is no significant correlation (SI Fig. 11). Similarly, we detect a significant, but weak correlation between pairwise genetic distance and geographic distance (SI Fig. 11). Both these observations are consistent with the other population structure results (see e.g. main text Fig. 2), that fail to detect some clear pattern among the ancient southern Africans who lived before 1.3 kya.

There is no contradiction in large genetic differentiation between individuals on the one hand, and the lack of temporal or geographic stratification. It is simply a consequence of the fact that the genetic variation is not co-varying with time and only mildly co-varying with geography. An even larger sample of ancient southern African individuals may reveal finer levels of stratification that may be correlated with time and/or geography. Future studies will shed

light on this question.

References

1000 Genomes Project Consortium 2015. A global reference for human genetic variation, *Nature* **526** (7571), 68.

Alexander, D., Novembre, J., and Lange, K. 2009. Fast model-based estimation of ancestry in unrelated individuals, *Genome Res* **19** (9), 1655–1664.

Alves, E., Macario, K., Urrutia, F., Cardoso, R., and Ramsey, C. 2019. Accounting for the marine reservoir effect in radiocarbon calibration, *Quaternary Science Reviews* **209**, 129–138.

Ambrose, S. and DeNiro, M. 1986. Reconstruction of african human diet using bone collagen carbon and nitrogen isotope ratios, *Nature* **319** (6051), 321–324.

Barbieri, C., Hübner, A., Macholdt, E., Ni, S., Lippold, S., Schröder, R., Mpoloka, S., Purps, J., Roewer, L., Stoneking, M., and Pakendorf, B. 2016. Refining the y chromosome phylogeny with southern african sequences, *Human genetics* **135** (5), 541–553.

Batini, C., Ferri, G., Destro-Bisol, G., Brisighelli, F., Luiselli, D., Sanchez-Diz, P., Rocha, J., Simonson, T., Brehm, A., Montano, V., Elwali, N. E., Spedini, G., D'Amato, M. E., Myres, N., Ebbesen, P., Comas, D., and Capelli, C. 2011. Signatures of the preagricultural peopling processes in sub-saharan africa as revealed by the phylogeography of early y chromosome lineages, *Molecular Biology and Evolution* **28** (9), 2603–2613.

Behr, A., Liu, K., Liu-Fang, G., Nakka, P., and Ramachandran, S. 2016. Pong: fast analysis and visualization of latent clusters in population genetic data, *Bioinformatics* **32** (18), 2817–2823.

Bergström, A., McCarthy, S., Hui, R., Almarri, M., Ayub, Q., Danecek, P., Chen, Y., Felkel, S., Hallast, P., Kamm, J., Blanché, H., Deleuze, J.-F., Cann, H., Mallick, S., Reich, D., Sandhu, M. S., Skoglund, P., Scally, A., Xue, Y., Durbin, R., and Tyler-Smith, C. 2020. Insights into human genetic variation and population history from 929 diverse genomes, *Science* **367** (6484).

Binneman, J. 2004. Archaeological research along the south-eastern cape coast part 1: Open-air shell middens, *Southern African Field Archaeology* **13** (14), 49–77.

Boeyens, J. 2003. The late iron age sequence in the marico and early tswana history, *South African Archaeological Bulletin* **58** (178), 63–78.

Boyle, E. A., Li, Y. I., and Pritchard, J. K. 2017. An Expanded View of Complex Traits: From Polygenic to Omnigenic, *Cell* **169**, 1177–1186.

Breton, G., Fortes-Lima, C., and Schlebusch, C. 2021. Revisiting the demographic history of central african populations from a genetic perspective, *Human Population Genetics and Genomics* **1** (1).

Breton, G., Schlebusch, C., Lombard, M., Sjödin, P., Soodyall, H., and Jakobsson, M. 2014. Lactase persistence alleles reveal partial east african ancestry of southern african khoe pastoralists, *Current Biology* **24** (8), 852–858.

Briggs, A. and Heyn, P. 2012. Preparation of next-generation sequencing libraries from damaged dna, *in* "Ancient DNA", pp. 143–154, Springer.

Chang, C.-F., Huang, S.-P., Hsueh, Y.-M., Geng, J.-H., Huang, C.-Y., and Bao, B.-Y. 2022. Genetic analysis implicates dysregulation of shank2 in renal cell carcinoma progression, *International Journal of Environmental Research and Public Health* **19** (19).

Chen, H. and Boutros, P. 2011. Venndiagram: a package for the generation of highly-customizable venn and euler diagrams in r, *BMC Bioinformatics* **12** (35).

Chen, L., Wolf, A., Fu, W., Li, L., and Akey, J. 2020. Identifying and interpreting apparent neanderthal ancestry in african individuals, *Cell* **180** (4), 677–687.

Choudhury, A., Ramsay, M., Hazelhurst, S., Aron, S., Bardien, S., Botha, G., Chimusa, E. R., Christoffels, A., Gamieldien, J., Sefid-Dashti, M. J., Joubert, F., Meintjes, A., Mulder, N., Ramesar, R., Rees, J., Scholtz, K., Sengupta, D., Soodyall, H., Venter, P., Warnich, L., and Pepper, M. S. 2017. Whole-genome sequencing for an enhanced understanding of genetic variation among south africans, *Nature Communication* **8** (1), 1–12.

Cingolani, P., Platts, A., Coon, M., Nguyen, T., Wang, L., Land, S., Lu, X., and Ruden, D. 2012. A program for annotating and predicting the effects of single nucleotide polymorphisms, snpeff: Snps in the genome of drosophila melanogaster strain w1118; iso-2; iso-3, *Fly* **6** (2), 80–92.

Clayton, F., Sealy, J., and Pfeiffer, S. 2006. Weaning age among foragers at matjes river rock shelter, south africa, from stable nitrogen and carbon isotope analyses, *American Journal Of Physical Anthropology* **129** (2), 311–317.

Cole, C. B., Zhu, S. J., Mathieson, I., Prüfer, K., and Lunter, G. 2020. Ancient admixture into africa from the ancestors of non-africans, *bioRxiv* , doi: 10.1101/2020.06.01.127555.

Coutinho, A., Malmström, H., Edlund, H., Henshilwood, C., van Niekerk, K., Lombard, M., Schlebusch, C., and Jakobsson, M. 2021. Later stone age human hair from vaalkrans shelter, cape floristic region of south africa, reveals genetic affinity to khoe groups, *American Journal of Physical Anthropology* **174** (4), 701–713.

Dabney, J., Knapp, M., Glocke, I., Gansauge, M.-T., Weihmann, A., Nickel, B., Valdiosera, C., Garcia, N., Paeaebo, S., Arsuaga, J.-L., and Meyer, M. 2013. Complete mitochondrial genome sequence of a middle pleistocene cave bear reconstructed from ultrashort dna fragments, *Proceedings of the National Academy of Sciences* **110** (39), 15758–15763.

Danecek, P., Auton, A., Abecasis, G., Albers, C., Banks, E., DePristo, M., Handsaker, R., Lunter, G., Marth, G., Sherry, S., McVean, G., Durbin, R., and 1000 Genomes Project Analysis Group 2011. The variant call format and vcftools, *Bioinformatics* **27** (15), 2156–2158.

Danecek, P., Bonfield, J. K., Liddle, J., Marshall, J., Ohan, V., Pollard, M. O., Whitwham, A., Keane, T., McCarthy, S. A., Davies, R. M., and Li, H. 2021. Twelve years of samtools and bcftools, *GigaScience* **10** (2), giab008.

de Ruiter, D., Brophy, J., Lewis, P., Churchill, S., and Berger, L. 2008. Faunal assemblage composition and paleoenvironment of plovers lake, a middle stone age locality in gauteng province, south africa, *Journal of Human Evolution* **55** (6), 1102–1117.

Deacon, J. 1984. “The later stone age of southernmost Africa”, BAR Publishing, Oxford.

Dreyer, T. 1931. The bushmen-hottentot-strandlooper tangle, *Transactions of the Royal Society of South Africa* **20** (1), 79–92.

Dreyer, T. 1933. The archaeology of the matjes river rock shelter, *Transactions of the Royal Society of South Africa* **21** (2), 187–209.

Durvasula, A. and Sankararaman, S. 2020. Recovering signals of ghost archaic introgression in african populations, *Science Advances* **6** (7).

Döckel, W. 2007. “Re-investigation of the Matjes River rock shelter”, PhD Thesis. Stellenbosch University, Stellenbosch.

Engelken, J., Carnero-Montoro, E., Pybus, M., Andrews, G. K., Lalueza-Fox, C., Comas, D., Sekler, I., de la Rasilla, M., Rosas, A., Stoneking, M., Valverde, M. A., Vicente, R., and Bosch, E. 2014. Extreme population differences in the human zinc transporter zip4 (slc39a4) are explained by positive selection in sub-saharan africa, *Plos Genetics* **10** (2).

Fan, S., Kelly, D. E., Beltrame, M. H., Hansen, M. E. B., Mallick, S., Ranciaro, A., Hirbo, J., Thompson, S., Beggs, W., Nyambo, T., Omar, S. A., Meskel, D. W., Belay, G., Froment, A., Patterson, N., Reich, D., and Tishkoff, S. A. 2019. African evolutionary history inferred from whole genome sequence data of 44 indigenous african populations, *Genome Biology* **20** (1), 1–14.

Fan, S., Spence, J., Feng, Y., Hansen, M., Terhorst, J., Beltrame, M., Ranciaro, A., Hirbo, J., Beggs, W., Thomas, N., Nyambo, T., Mpoloka, S., Mokone, G., Njamnshi, A., Folkunang, C., Meskel, D., Belay, G., Song, Y., and Tishkoff, S. 2023. Whole-genome sequencing reveals a complex african population demographic history and signatures of local adaptation, *Cell* **186** (5), 923–939.

Forssman, T. 2023. The end of the later stone age in the middle limpopo valley, central southern africa, *Journal of Paleolithic Archaeology* **6**.

Fu, Q., Li, H., Moorjani, P., Jay, F., Slepchenko, S., Bondarev, A., Johnson, P., Aximu-Petri, A., Pruefer, K., de Filippo, C., Meyer, M., Zwyns, N., Salazar-Garcia, D., Kuzmin, Y., Keates, S., Kosintsev, P., Razhev, D., Richards, M., Peristov, N., Lachmann, M., Douka, K., Higham, T., Slatkin,

M., Hublin, J.-J., Reich, D., Kelso, J., Viola, T., and Paeaebo, S. 2014. Genome sequence of a 45,000-year-old modern human from western siberia, *Nature* **514** (7523), 445+.

Fu, Q., Mittnik, A., Johnson, P., Bos, K., Lari, M., Bollongino, R., Sun, C., Giemsch, L., Schmitz, R., Burger, J., Ronchitelli, A., Martini, F., Cremonesi, R., Svoboda, J., Bauer, P., Caramelli, D., Castellano, S., Reich, D., Pääbo, S., and Krause, J. 2013. A revised timescale for human evolution based on ancient mitochondrial genomes, *Nature Reviews Genetics* **23**, 553–559.

Green, R., J.Krause, Briggs, A., Maricic, T., Stenzel, U., Kircher, M., Patterson, N., Li, H., Zhai, W., Fritz, M.-Y., Hansen, N., Durand, E., Malaspinas, A.-S., Jensen, J., T.Marques-Bonet, Alkan, C., Prüfer, K., Meyer, M., Burbano, H., Good, J., Schultz, R., Aximu-Petri, A., Butthof, A., Höber, B., Höffner, B., Siegemund, M., Weihmann, A., Nusbaum, C., Lander, E., Russ, C., Novod, N., Affourtit, J., Egholm, M., Verna, C., Rudan, P., Brajkovic, D., Kucan, Z., Gusic, I., Doronichev, V., Golovanova, L., Lalueza-Fox, C., de la Rasilla, M., Fortea, J., Rosas, A., Schmitz, R., Johnson, P., Eichler, E., Falush, D., Birney, E., Mullikin, J., Slatkin, M., Nielsen, R., Kelso, J., Lachmann, M., Reich, D., and Pääbo, S. 2010. A draft sequence of the neandertal genome, *Science* **328** (5979), 710–722.

Green, R. E., Malaspinas, A.-S., Krause, J., Briggs, A. W., Johnson, P. L. F., Uhler, C., Meyer, M., Good, J. M., Maricic, T., Stenzel, U., Pruefer, K., Siebauer, M., Burbano, H. A., Ronan, M., Rothberg, J. M., Egholm, M., Rudan, P., Brajkovic, D., Kucan, Z., Gusic, I., Wikstrom, M., Laakkonen, L., Kelso, J., Slatkin, M., and Paeaebo, S. 2008. A complete neandertal mitochondrial genome sequence determined by high-throughput sequencing, *Cell* **134** (3), 416–426.

Grine, F. 2023. Description and comparative morphology of the hofmeyr skull, *in* "Hofmeyr: a Late Pleistocene human skull from South Africa", pp. 71–118, Cham: Springer International Publishing.

Gronau, I., Hubisz, M., Gulko, J., Danko, B., and Siepel, C. 2011. Bayesian inference of ancient human demography from individual genome sequences., *Nature Genetics* **43**, 1031–1035.

Gurdasani, D., Carstensen, T., Tekola-Ayele, F., Pagani, L., Tachmazidou, I.,

Hatzikotoulas, K., Karthikeyan, S., Iles, L., Pollard, M. O., Choudhury, A., Ritchie, G. S., Xue, Y., Asimit, J., Nsubuga, R. N., Young, E. H., Pomilla, C., Kivinen, K., Rockett, K., Kamali, A., Doumatey, A. P., Asiki, G., Seeley, J., Sisay-Joof, F., Jallow, M., Tollman, S., Mekonnen, E., Ekong, R., Oljira, T., Bradman, N., Bojang, K., Ramsay, M., Adeyemo, A., Bekele, E., Motala, A., Norris, S. A., Pirie, F., Kaleebu, P., Kwiatkowski, D., Tyler-Smith, C., Rotimi, C., Zeggini, E., and Sandhu, M. S. 2015. The african genome variation project shapes medical genetics in africa, *Nature* **517** (7534), 327–332.

Günther, T., Malmström, H., Svensson, E., Omrak, A., Sánchez-Quinto, F., Kılınç, G., Krzewińska, M., Eriksson, G., Fraser, M., Edlund, H., Munters, A., Coutinho, A., Simões, L., Vicente, M., Sjölander, A., SB., J., Jørgensen, R., Claes, P., Shriver, M., Valdiosera, C., Netea, M., Apel, J., Lidén, K., Skar, B., Storå, J., Götherström, A., and Jakobsson, M. 2018. Population genomics of mesolithic scandinavia: Investigating early postglacial migration routes and high-latitude adaptation, *PLoS Biology* **16** (1), 1–22.

Günther, T., Valdiosera, C., Malmstrom, H., Urena, I., Rodriguez-Varela, R., Sverrisdottir, O. O., Daskalaki, E. A., Skoglund, P., Naidoo, T., Svensson, E. M., Maria Bermudez de Castro, J., Carbonell, E., Dunn, M., Stora, J., Iriarte, E., Arsuaga, J. L., Carretero, J.-M., Götherstrom, A., and Jakobsson, M. 2015. Ancient genomes link early farmers from atapuerca in spain to modern-day basques, *Proceedings of the National Academy of Sciences* **112** (38), 11917–11922.

Hasan, M. A., Hakim, F. T., Islam Shovon, M. T., Islam, M. M., Islam, M. S., and Islam, M. A. 2021. The investigation of nonsynonymous snps of human slc6a4 gene associated with depression: An in silico approach, *Heliyon* **7** (8), e07815.

Henn, B., Gignoux, C., Lin, A., Oefner, P., Shen, P., Scozzari, R., Cruciani, F., Tishkoff, S., Mountain, J., and Underhill, P. 2008. Y-chromosomal evidence of a pastoralist migration through tanzania to southern africa, *Proceedings of the National Academy of Sciences* **105** (31), 10693–10698.

Hernandez-Pacheco, N., Flores, C., Alonso, S., Eng, C., Mak, A. C. Y., Hunstman, S., Hu, D., White, M. J., Oh, S. S., Meade, K., Farber, H. J., Avila, P. C., Serebrisky, D., Thyne, S. M., Brigino-Buenaventura, E., Rodriguez-

Cintron, W., Sen, S., Kumar, R., Lenoir, M., Rodriguez-Santana, J. R., Burchard, E. G., and Pino-Yanes, M. 2017. Identification of a novel locus associated with skin colour in african-admixed populations, *Scientific Reports* **7**.

Hoffman, A. 1958. New excavations in the matjes river rock shelter, *South African Museums Association Bulletin* **6** (11), 342–348.

Hoffman, A. 1962. Pigmyation of the bushman race, *Navorsinge van die Nasionale Museum: Researches of the National Museum* **1** (12), 281–286.

Hogg, A., Heaton, T., Hua, Q., Palmer, J., Turney, C., Southon, J., Bayliss, A., Blackwell, P., Boswijk, G., Ramsey, C., Pearson, C., Petchey, F., Reimer, P., Reimer, R., and Wacker, L. 2020. Shcal20 southern hemisphere calibration, 0-55,000 years cal bp, *Radiocarbon* **62** (4), 759–778.

Hogg, A., Hua, Q., Blackwell, P., Niu, M., Buck, C., Guilderson, T., Heaton, T., Palmer, J., Reimer, P., Reimer, R., Turney, C., and Zimmerman, S. 2013. Shcal13 southern hemisphere calibration, 0-50,000 years cal bp, *Radiocarbon* **55** (4, SI), 1889–1903.

Hollfelder, N., Breton, G., Sjodin, P., and Jakobsson, M. 2021. The deep population history in africa, *Human Molecular Genetics* **30** (R1), R2–R10.

Huffman, T. 2007. Leokwe and k2: Ethnic stratification during the middle iron age in southern africa, *Journal Of African Archaeology* **5** (2), 163–188.

Huffman, T. 2009. A cultural proxy for drought: ritual burning in the iron age of southern africa, *Journal Of Archaeological Science* **36** (4), 991–1005.

Humphreys, A. 1970. The remains from koffiefontein burials excavated by w. fowler and preserved in the mcgregor museum, kimberley, *The South African Archaeological Bulletin* **25** (99/100), 104–115.

Humphreys, A. 1972. "The Type R settlements in the context of the later prehistory and early history of the Riet River valley", Master's Dissertation, University of Cape Town.

Ijaz, A., Wolf, S., Mandukhail, S. R., Basit, S., Betz, R. C., and Wali, A. 2019. Uv-sensitive syndrome: Whole exome sequencing identified a nonsense mutation in the gene uvssa in two consanguineous pedigrees from pakistan, *Journal Of Dermatological Science* **95** (3), 113–118.

Jerardino, A. 1998. Excavations at pancho's-kitchen-midden, western cape coast, south africa: Further observations into the megamidden period, *South African Archaeological Bulletin* **53** (167), 16–25.

Jerardino, A. 2007. "Changing social landscapes of the western Cape coast of southern Africa over the last 4500 years", PhD Thesis. University of Cape Town, Cape Town.

Jones, E., Gonzalez-Fortes, G. ans Connell, S., Siska, V., Eriksson, A., Martiniano, R., McLaughlin, R., Llorente, M., Cassidy, L., Gamba, C., Meshveliani, T., Bar-Yosef, O., Müller, W., Belfer-Cohen, A., Matskevich, Z., Jakeli, N., Higham, T., Currat, M., Lordkipanidze, D., Hofreiter, M., Manica, A., Pinhasi, R., and Bradley, D. 2015. Upper palaeolithic genomes reveal deep roots of modern eurasians, *Nat Commun* **6** (8912).

Jun, G., Flickinger, M., Hetrick, K., Romm, J., Doheny, K., Abecasis, G., Boehnke, M., and Kang, H. 2012. Detecting and estimating contamination of human dna samples in sequencing and array-based genotype data, *The American Journal of Human Genetics* **91** (5), 839–848.

Keith, A. 1933. A descriptive account of the human skulls from matjes river cave, cape province, *Transactions of the Royal Society of South Africa* **21** (2), 151–185.

Kircher, M. 2012. Analysis of high-throughput ancient dna sequencing data, *in* "Ancient DNA", pp. 197–228, Springer.

L'abbe, E., Loots, M., and Keough, N. 2008. The matjes river rock shelter:: A description of the skeletal assemblage, *South African Archaeological Bulletin* **63** (187), 61–68.

Lahr, M. and Foley, R. 1998. Towards a theory of modern human origins: Geography, demography, and diversity in recent human evolution, *Yearbook of Physical Anthropology* **27**, 137–176.

Lappalainen, T., Li, Y., Ramachandran, S., and Gusev, A. 2024. Genetic and molecular architecture of complex traits, *Cell* **187** (5), 1059–1075.

Lazaridis, I., Patterson, N., Mittnik, A., Renaud, G., Mallick, S., Kirsanow, K., Sudmant, P., Schraiber, J., Castellano, S., Lipson, M., Berger, B., Economou, C., Bollongino, R., Fu, Q., Bos, K., Nordenfelt, S., Li, H., de Filippo, C., Prüfer, K., Sawyer, S., Posth, C., Haak, W., Hallgren, F.,

Fornander, E., Rohland, N., Delsate, D., Francken, M., Guinet, J., Wahl, J., Ayodo, G., Babiker, H., Bailliet, G., Balanovska, E., Balanovsky, O., Barrantes, R., Bedoya, G., Ben-Ami, H., Bene, J., Berrada, F., Bravi, C., Brisighelli, F., Busby, G., Cali, F., Churnosov, M., Cole, D., Corach, D., Damba, L., van Driem, G., Dryomov, S., Dugoujon, J., Fedorova, S., Gallego Romero, I., Gubina, M., Hammer, M., Henn, B., Hervig, T., Hodoglugil, U., Jha, A., Karachanak-Yankova, S., Khusainova, R., Khusnutdinova, E., Kittles, R., Kivisild, T., Klitz, W., Kučinskas, V., Kushniarevich, A., Laredj, L., Litvinov, S., Loukidis, T., Mahley, R., Melegh, B., Metspalu, E., Molina, J., Mountain, J., Näkkäläjärvi, K., Nesheva, D., Nyambo, T., Osipova, L., Parik, J., Platonov, F., Posukh, O., Romano, V., Rothhammer, F., Rudan, I., Ruizbakiev, R., Sahakyan, H., Sajantila, A., Salas, A., Starikovskaya, E., Tarekegn, A., Toncheva, D., Turdikulova, S., Uktveryte, I., Utevska, O., Vasquez, R., Villena, M., Voevoda, M., Winkler, C., Yepiskoposyan, L., Zalloua, P., Zemunik, T., Cooper, A., Capelli, C., Thomas, M., Ruiz-Linares, A., Tishkoff, S., Singh, L., Thangaraj, K., Villems, R., Comas, D., Sukernik, R., Metspalu, M., Meyer, M., Eichler, E., Burger, J., Slatkin, M., Pääbo, S., Kelso, J., Reich, D., and Krause, J. 2014. Ancient human genomes suggest three ancestral populations for present-day europeans, *Nature* **513**, 409–13.

Lee-Thorp, J., Sealy, J., and Morris, A. 1993. Isotopic evidence for diets of prehistoric farmers in south africa, *in* "Prehistoric Human Bone", pp. 99–120, Springer, Berlin, Heidelberg.

Lewis, M. and Sealy, J. 2018. Coastal complexity: Ancient human diets inferred from bayesian stable isotope mixing models and a primate analogue, *PLoS One* **13** (12).

Li, H. and Durbin, R. 2009. Fast and accurate short read alignment with burrows–wheeler transform, *Bioinformatics* **25** (14), 1754–1760.

Li, H., Handsaker, B., Wysoker, A., Fennell, T., Ruan, J., Homer, N., Marth, G., Abecasis, G., and Durbin, R. 2009. The sequence alignment/map format and samtools, *Bioinformatics* **25** (16), 2078–2079.

Lipson, M., Ribot, I., Mallick, S., Rohland, N., Olalde, I., Adamski, N., Broomandkhoshbacht, N., Lawson, A. M., Lopez, S., Oppenheimer, J., Stewardson, K., Asombang, R. N., Bocherens, H., Bradman, N., Culleton, B. J., Cornelissen, E., Crevecoeur, I., de Maret, P., Fomine, F. L. M.,

Lavachery, P., Mindzie, C. M., Orban, R., Sawchuk, E., Semal, P., Thomas, M. G., Van Neer, W., Veeramah, K. R., Kennett, D. J., Patterson, N., Hellenthal, G., Lalueza-Fox, C., MacEachern, S., Prendergast, M. E., and Reich, D. 2020. Ancient west african foragers in the context of african population history, *Nature* **577** (7792), 665–670.

Lipson, M., Sawchuk, E. A., Thompson, J. C., Oppenheimer, J., Tryon, C. A., Ranhorn, K. L., de Luna, K. M., Sirak, K. A., Olalde, I., Ambrose, S. H., Arthur, J. W., Arthur, K. J. W., Ayodo, G., Bertacchi, A., Cerezo-Roman, I, J., Culleton, B. J., Curtis, M. C., Davis, J., Gidna, A. O., Hanson, A., Kaliba, P., Katongo, M., Kwekason, A., Laird, M. F., Lewis, J., Mabulla, A. Z. P., Mapemba, F., Morris, A., Mudenda, G., Mwafulirwa, R., Mwangomba, D., Ndiema, E., Ogola, C., Schilt, F., Willoughby, P. R., Wright, D. K., Zipkin, A., Pinhasi, R., Kennett, D. J., Manthi, F. K., Rohland, N., Patterson, N., Reich, D., and Prendergast, M. E. 2022. Ancient dna and deep population structure in sub-saharan african foragers, *Nature* **603** (7900), 290–296.

Llorente, M., Jones, E., Eriksson, A., Siska, V., Arthur, K., Arthur, J., Curtis, M., Stock, J., Coltorti, M., Pieruccini, P., Stretton, S., Brock, F., Higham, T., Park, Y., Hofreiter, M., Bradley, D., Bhak, J., Pinhasi, R., and Manica, A. 2015. Ancient ethiopian genome reveals extensive eurasian admixture in eastern africa, *Science* **350** (6262), 820–822.

Loftus, E., Lombard, M., and Steyn, M. 2024. Dated holocene human remains from south africa: Recalibration and broad contextualization, *Radiocarbon* **66** (1), 118–133.

Lombard, M. 2020a. The tip cross-sectional areas of poisoned bone arrowheads from southern africa, *Journal Of Archaeological Science-Reports* **33**.

Lombard, M. 2020b. Testing for poisoned arrows in the middle stone age: A tip cross-sectional analysis of backed microliths from southern africa, *Journal Of Archaeological Science-Reports* **34** (A).

Lombard, M., Bradfield, J., Caruana, V., Makhubelal, V., Dusseldorp, G., Kramers, J., and Wurz, S. 2022. The southern african stone age ssquence updated (ii), *South African Archaeological Bulletin* **77** (217), 172–212.

Lombard, M., Malmström, H., Schlebusch, C., Svensson, E., Günther, T., Munters, A., Coutinho, A., Edlund, H., Zipfel, B., and Jakobsson, M.

2019. Genetic data and radiocarbon dating question plovers lake as a middle stone age hominin-bearing site, *Journal of Human Evolution* **131**, 203–209.

Lombard, M. and Parsons, I. 2015. Milk not meat: the role of milk amongst the khoe peoples of southern africa, *Journal Of African Archaeology* **13** (2), 149–166.

Lombard, M., Schlebusch, C., and Soodyall, H. 2013. Bridging disciplines to better elucidate the evolution of early homo sapiens in southern africa, *South African Journal of Sciences* **109**.

Louw, J. T. 1960. "Prehistory of the Matjes River Rock Shelter", National Museum of Bloemfontein.

Ludwig, B. 2005. "A comparison of hunter-gatherer material culture from Matjes River Rock Shelter and Nelson Bay Cave", Master's Dissertation, University of Cape Town.

Mafessoni, F., Grote, S., de Filippo, C., Slon, V., Kolobova, K., Viola, B., Markin, S., Chintalapati, M., Peyrégne, S., Skov, L., Skoglund, P., Krivoshapkin, A., Derevianko, A., Meyer, M., Kelso, J., Peter, B., Prüfer, K., and Pääbo, S. 2020. A high-coverage neandertal genome from chagyrskaya cave, *Proceedings of the National Academy of Sciences* **117** (26), 15132–15136.

Maggs, T. 1971. Pastoral settlements on the riet river, *The South African Archaeological Bulletin* **26**, 37–63.

Mallick, S., Li, H., Lipson, M., Mathieson, I., Gymrek, M., Racimo, F., Zhao, M., Chennagiri, N., Nordenfelt, S., Tandon, A., Skoglund, P., Lazaridis, I., Sankararaman, S., Fu, Q., Rohland, N., Renaud, G., Erlich, Y., Willems, T., Gallo, C., Spence, J. P., Song, Y. S., Poletti, G., Balloux, F., van Driem, G., de Knijff, P., Romero, I. G., Jha, A. R., Behar, D. M., Bravi, C. M., Capelli, C., Hervig, T., Moreno-Estrada, A., Posukh, O. L., Balanovska, E., Balanovsky, O., Karachanak-Yankova, S., Sahakyan, H., Toncheva, D., Yepiskoposyan, L., Tyler-Smith, C., Xue, Y., Abdullah, M. S., Ruiz-Linares, A., Beall, C. M., Di Rienzo, A., Jeong, C., Starikovskaya, E. B., Metspalu, E., Parik, J., Villems, R., Henn, B. M., Hodoglugil, U., Mahley, R., Sajantila, A., Stamatoyannopoulos, G., Wee, J. T. S., Khusainova, R., Khusnutdinova, E., Litvinov, S., Ayodo, G., Co-

mas, D., Hammer, M. F., Kivisild, T., Klitz, W., Winkler, C. A., Labuda, D., Bamshad, M., Jorde, L. B., Tishkoff, S. A., Watkins, W. S., Metspalu, M., Dryomov, S., Sukernik, R., Singh, L., Thangaraj, K., Paeaebo, S., Kelso, J., Patterson, N., and Reich, D. 2016. The simons genome diversity project: 300 genomes from 142 diverse populations, *Nature* **538** (7624), 201–206.

Malmström, H., Svensson, E., Gilbert, M., Willerslev, E., Götherström, A., and Holmlund, G. 2007. More on contamination: the use of asymmetric molecular behavior to identify authentic ancient human dna, *Molecular Biology and Evolution* **24** (4), 998–1004.

Mao, Y., Francis-West, P., and Irvine, K. D. 2015. Fat4/dchs1 signaling between stromal and cap mesenchyme cells influences nephrogenesis and ureteric bud branching, *Development* **142** (15), 2574–U69.

Mao, Y., Mulvaney, J., Zakaria, S., Yu, T., Morgan, K. M., Allen, S., Basson, M. A., Francis-West, P., and Irvine, K. D. 2011. Characterization of a dchs1 mutant mouse reveals requirements for dchs1-fat4 signaling during mammalian development, *Development* **138** (5), 947–957.

Mazet, O., Rodriguez, W., Grusea, S., Boitard, S., and Chikhi, L. 2016. On the importance of being structured: instantaneous coalescence rates and human evolution–lessons for ancestral population size inference?, *Heredity* **116**, 362–371.

McKenna, J., Bernhardsson, C., Waxman, D., Jakobsson, M., and Sjödin, P. 2024. Investigating population continuity and ghost admixture among ancient genomes, *Hum Popul Genet Genom* **4** (3), 0009.

Meiring, A. 1937. The frontal convolutions on the endocranial cast of the skull mri from the deepest levels of the matjes river cave, cp, *South African Journal of Science* **33** (3), 960–970.

Meyer, M. and Kircher, M. 2010. Illumina sequencing library preparation for highly multiplexed target capture and sequencing, *Cold Spring Harbor Protocols* **2010** (6), pdb–prot5448.

Meyer, M., Kircher, M., Gansauge, M., Li, H., Racimo, F., Mallick, S., Schraiber, J., Jay, F., Prüfer, K., de Filippo, C., Sudmant, P., Alkan, C., Fu, Q., Do, R., Rohland, N., Tandon, A., Siebauer, M., Green, R., Bryc, K., Briggs, A., Stenzel, U., Dabney, J., Shendure, J., Kitzman, J.,

Hammer, M., Shunkov, M., Derevianko, A., Patterson, N., Andrés, A., Eichler, E., Slatkin, M., Reich, D., Kelso, J., and Pääbo, S. 2012. A high-coverage genome sequence from an archaic denisovan individual, *Science* **338** (6104), 222–226.

Minagawa, M. and Wada, E. 1984. Stepwise enrichment of n-15 along food-chains - further evidence and the relation between delta-n-15 and animal age, *Geochimica Et Cosmochimica Acta* **48** (5), 1135–1140.

Montinaro, F., Busby, G., Gonzalez-Santos, M., Oosthuitzen, O., Oosthuitzen, E., Anagnostou, P., Destro-Bisol, G., Pascali, V., and Capelli, C. 2017. Complex ancient genetic structure and cultural transitions in southern african populations, *Genetics* **205** (1), 303–316.

Morris, A. 1981. Copper discolouration of bone and the incidence of copper artefacts with human burials in south africa, *The South African Archaeological Bulletin* **36**, 36–42.

Morris, A. 1992a. "A master catalogue: Holocene human skeletons from South Africa", Witwatersrand University Press, Johannesburg.

Morris, A. 1992b. "The skeletons of contact: A study of protohistoric burials from the Lower Orange River Valley, South Africa", Witwatersrand University Press, Johannesburg.

Mukhi, D., Li, L., Liu, H., Doke, T., Kolligundla, L. P., Ha, E., Kloetzer, K., Abedini, A., Mukherjee, S., Wu, J., Dhillon, P., Hu, H., Guan, D., Funai, K., Uehara, K., Titchenell, P. M., Baur, J. A., Wellen, K. E., and Susztak, K. 2024. Acss2 gene variants determine kidney disease risk by controlling de novo lipogenesis in kidney tubules, *Journal Of Clinical Investigation* **134** (4).

Naidoo, T., Schlebusch, C., Makkan, H., Patel, P., Mahabeer, R., Erasmus, J., and Soodyall, H. 2010. Development of a single base extension method to resolve y chromosome haplogroups in sub-saharan african populations, *Investigative genetics* **1** (1), 1–11.

Naidoo, T., Xu, J., Vicente, M., Malmström, H., Soodyall, H., Jakobsson, M., and Schlebusch, C. 2020. Y-chromosome variation in southern african khoe-san populations based on whole-genome sequences, *Genome Biology and Evolution* **12** (7), 1031–1039.

Neuweger, D. 2007. "Khoe San Cranial Variation: A Study of the Matjes River Rock Shelter Crania", PhD Thesis. University of New South Whales, Sydney.

Patterson, N., Price, A., and Reich, D. 2006. Population structure and eigenanalysis, *PLoS Genetics* **2** (12), e190.

Pontzer, H., Brown, M. H., Wood, B. M., Raichlen, D. A., Mabulla, A. Z. P., Harris, J. A., Dunsworth, H., Hare, B., Walker, K., Luke, A., Dugas, L. R., Schoeller, D., Plange-Rhule, J., Bovet, P., Forrester, T. E., Thompson, M. E., Shumaker, R. W., Rothman, J. M., Vogel, E., Sulistyo, F., Alavi, S., Prasetyo, D., Urlacher, S. S., and Ross, S. R. 2021. Evolution of water conservation in humans, *Current Biology* **31** (8), 1804+.

Prendergast, M. E., Lipson, M., Sawchuk, E. A., Olalde, I., Ogola, C. A., Rohland, N., Sirak, K. A., Adamski, N., Bernardos, R., Broomandkhoshbacht, N., Callan, K., Culleton, B. J., Eccles, L., Harper, T. K., Lawson, A. M., Mah, M., Oppenheimer, J., Stewardson, K., Zalzala, F., Ambrose, S. H., Ayodo, G., Gates, Jr., H. L., Gidna, A. O., Katongo, M., Kwekason, A., Mabulla, A. Z. P., Mudenda, G. S., Ndiema, E. K., Nelson, C., Robertshaw, P., Kennett, D. J., Manthi, F. K., and Reich, D. 2019. Ancient dna reveals a multistep spread of the first herders into sub-saharan africa, *Science* **365** (6448), eaaw6275.

Price, A., Patterson, N., Plenge, R., Weinblatt, M., Shadick, N., and Reich, D. 2006. Principal components analysis corrects for stratification in genome-wide association studies, *Nature Genetics* **38** (8), 904–909.

Prüfer, K. 2018. snpAD: an ancient DNA genotype caller, *Bioinformatics* **34** (24), 4165–4171.

Prüfer, K., Racimo, F., Patterson, N., Jay, F., Sankararaman, S., Sawyer, S., Heinze, A., Renaud, G., Sudmant, P., de Filippo, C., Li, H., Mallick, S., Dannemann, M., Fu, Q., Kircher, M., Kuhlwilm, M., Lachmann, M., Meyer, M., Ongyerth, M., Siebauer, M., Theunert, C., Tandon, A., Moorjani, P., Pickrell, J., Mullikin, J., Vohr, S., Green, R., Hellmann, I., Johnson, P., Blanche, H., Cann, H., Kitzman, J., Shendure, J., Eichler, E., Lein, E., Bakken, T., Golovanova, L., Doronichev, V., Shunkov, M., Derevianko, A., Viola, B., Slatkin, M., Reich, D., Kelso, J., and Pääbo, S. 2014. The complete genome sequence of a neanderthal from the altai mountains, *Nature* **505**, 43–49.

R Core Team 2021. "R: A Language and Environment for Statistical Computing". R Foundation for Statistical Computing Vienna, Austria.

Ragsdale, A. P., Weaver, T. D., Atkinson, E. G., Hoal, E. G., Moeller, M., Henn, B. M., and Gravel, S. 2023. A weakly structured stem for human origins in africa, *Nature* **620** (7972), E11.

Ramsey, C. 2009. Bayesian analysis of radiocarbon dates, *Radiocarbon* **51** (1), 337–360.

Raven-Hart, R. 2007. "Before Van Riebeeck: Callers at South Africa from 1488 to 1652", Struik, Cape Town.

Reich, D., Green, R., Kircher, M., Krause, J., Patterson, N., Durand, E., Viola, B., Briggs, A., Stenzel, U., Johnson, P., Maricic, T., Good, J., Marques-Bonet, T., Alkan, C., Fu, Q., Mallick, S., Li, H., Meyer, M., Eichler, E., Stoneking, M., Richards, M., Talamo, S., Shunkov, M., Derevianko, A., Hublin, J., Kelso, J., and Slatkin, M. Pääbo, S. 2010. Genetic history of an archaic hominin group from denisova cave in siberia, *Nature* **468**, 1053–1060.

Ribot, I., Ghalem, Y., and Crevecoeur, I. 2023. The position of the hofmeyr skull within late pleistocene and holocene african regional diversity: 2d and 3d morphometric analyses, *in* "In Hofmeyr: A Late Pleistocene human skull from South Africa", pp. 119–141, Cham: Springer International Publishing.

Ribot, I., Morris, A., Sealy, J., and Maggs, T. 2010. Population history and economic change in the last 2000 years in kwazulu-natal, rsa, *Southern African Humanities* **22**, 89–112.

Richards, M.P.and Schulting, R. and Hedges, R. 2003. Sharp shift in diet at onset of neolithic, *Nature* **425**, 366.

Scerri, E., Thomas, M., Manica, A., Gunz, P., Stock, J., Stringer, C., Grove, M., Groucutt, H., Timmermann, A., Rightmire, G., d'Errico, F., Tryon, C., Drake, N., Brooks, A., Dennell, R., Durbin, R., Henn, B., Lee-Thorp, J., deMenocal, P., Petraglia, M., Thompson, J., Scally, A., and Chikhi, L. 2018. Did our species evolve in subdivided populations across aafrica, and why does it matter?, *Trends in Ecology & Evolution* **33** (8), 582 – 594.

Schiffels, S. and Durbin, R. 2014. Inferring human population size and sepa-

ration history from multiple genome sequences, *Nature Genetics* **46**, 919–925.

Schlebusch, C. 2010. “Genetic variation in Khoisan-speaking populations from southern Africa”. PhD thesis University of the Witwatersrand Johannesburg (South Africa).

Schlebusch, C. and Jakobsson, M. 2018. Tales of human migration, admixture, and selection in africa, *Annual Review of Genomics and Human Genetics* **19** (1).

Schlebusch, C., Lombard, M., and Soodyall, H. 2013. Mtdna control region variation affirms diversity and deep sub-structure in populations from southern africa, *BMC Evolutionary Biology* **13**.

Schlebusch, C., Malmström, H., Günther, T., Sjödin, P., Coutinho, A., Edlund, H., Munters, A., Vicente, M., Steyn, M., Soodyall, H., Lombard, M., and Jakobsson, M. 2017. Southern african ancient genomes estimate modern human divergence to 350,000 to 260,000 years ago, *Science* **358** (6363), 652–655.

Schlebusch, C., Skoglund, P., Sjödin, P., Gattepaille, L., Hernandez, D., Jay, F., Li, S., De Jongh, M., Singleton, A., Blum, M., Soodyall, H., and Jakobsson, M. 2012. Genomic variation in seven khoe-san groups reveals adaptation and complex african history, *Science* **338** (6105), 374–379.

Schlebusch, C. M., Sjodin, P., Breton, G., Gunther, T., Naidoo, T., Hollfelder, N., Sjostrand, A. E., Xu, J., Gattepaille, L. M., Vicente, M., Scofield, D. G., Malmstrom, H., de Jongh, M., Lombard, M., Soodyall, H., and Jakobsson, M. 2020. Khoe-san genomes reveal unique variation and confirm the deepest population divergence in homo sapiens, *Molecular Biology and Evolution* **37** (10), 2944–2954.

Schoeninger, M., Nelson, B., and DeNiro, M. 1984. Bone strontium levels in modern animals and strontium isotopic evidence for diagenetic alteration of bone - consequences for diet reconstruction, *American Journal of Physical Anthropology* **63** (2), 216.

Schubert, M., Lindgreen, S., and Orlando, L. 2016. Adapterremoval v2: rapid adapter trimming, identification, and read merging, *BMC Research Notes* **9** (1), 1–7.

Sealy, J. 2006. Diet, mobility, and settlement pattern among holocene hunter-gatherers in southernmost africa, *Current Anthropology* **47** (4), 569–595.

Sealy, J. 2016. Intensification, diet, and group boundaries among later stone age coastal hunter-gatherers along the western and southern coasts of south africa, *in* "The Oxford Handbook of the Archaeology of Diet", Oxford University Press.

Sealy, J., Ludwig, B., and Henderson, Z. 2006. New radiocarbon dates for matjes river rock shelter, *South African Archaeological Bulletin* **61** (183), 98–101.

Sealy, J., Pfeiffer, S., Yates, R., Willmore, K., Manhire, A., Maggs, T., and Lanham, J. 2000. Hunter-gatherer child burials from the pakhuis mountains, western cape: Growth, diet and burial practices in the late holocene, *The South African Archaeological Bulletin* **55** (171), 32–43.

Sealy, J. and Van Der Merwe, N. 1986. Isotope assessment and the seasonal-mobility hypothesis in the southwestern cape of south-africa, *Current Anthropology* **27** (2), 135–150.

Simões, L., Peyroteo-Stjerna, R., Marchand, G., Bernhardsson, C., Vialet, A., Chetty, D., Alaçamlı, E., Edlund, H., Bouquin, D., Dina, C., Garmond, N., Günther, T., and Jakobsson, M. 2024. Genomic ancestry and social dynamics of the last hunter-gatherers of atlantic france, *Proceedings of the National Academy of Sciences* **121** (10), e2310545121.

Simões, L. G., Günther, T., Martínez-Sánchez, R., Vera-Rodríguez, J., Iriarte, E., Rodríguez-Varela, R., Bokbot, Y., Valdiosera, C., and Jakobsson, M. 2023. Northwest african neolithic initiated by migrants from iberia and levant., *Nature* **618**, 550–556.

Sjödin, P., McKenna, J., and Jakobsson, M. 2021. Estimating divergence times from dna sequences, *Genetics* **217** (4), iyab008.

Skoglund, P., Mallick, S., Bortolini, M., Chennagiri, N., Hünemeier, T., Petzl-Erler, M., Salzano, F., Patterson, N., and Reich, D. 2015. Genetic evidence for two founding populations of the americas, *Nature* **525**.

Skoglund, P., Malmström, H., Omrak, A., Raghavan, M., Valdiosera, C., Günther, T., Hall, P., Tambets, K., Parik, J., Sjögren, K.-G., Apel, J., Willerslev, E., Storå, J., Götherström, A., and Jakobsson, M. 2014. Ge-

nomic diversity and admixture differs for stone-age scandinavian foragers and farmers, *Science* **344** (6185), 747–750.

Skoglund, P., Malmström, H., Raghavan, M., Storå, J., Hall, P., Willerslev, E., Gilbert, M., Götherström, A., and Jakobsson, M. 2012. Origins and genetic legacy of neolithic farmers and hunter-gatherers in europe, *Science* **336**, 466–469.

Skoglund, P., Northoff, B., Shunkov, M., Derevianko, A., Pääbo, S., Krause, J., and Jakobsson, M. 2014. Separating endogenous ancient dna from modern day contamination in a siberian neandertal, *Proceedings of the National Academy of Sciences* **111** (6), 2229–2234.

Skoglund, P., Storå, J., Götherström, A., and Jakobsson, M. 2013. Accurate sex identification of ancient human remains using dna shotgun sequencing, *Journal of Archaeological Science* **40** (12), 4477–4482.

Skoglund, P., Thompson, J., Prendergast, M., Mittnik, A., Sirak, K., Hajdinjak, M., Salie, T., Rohland, N., Mallick, S., Peltzer, A., Heinze, A., Olalde, I., Ferry, M., Harney, E., Michel, M., Stewardson, K., Cerezo-Román, J., Chiumia, C., Crowther, A., Gomani-Chindebvu, E., Gidna, A., Grillo, K., Helenius, I., Hellenthal, G., Helm, R., Horton, M., López, S., Mabulla, A., Parkington, J., Shipton, C., Thomas, M., Tibesasa, R., Welling, M., Hayes, V., Kennett, D., Ramesar, R., Meyer, M., Pääbo, S., Patterson, N., Morris, A., Boivin, N., Pinhasi, R., Krause, J., and Reich, D. 2017. Reconstructing prehistoric african population structure, *Cell* **171**, 59–71.

Spiegel, E. K., Colman, R. F., and Patterson, D. 2006. Adenylosuccinate lyase deficiency, *Molecular Genetics and Metabolism* **89** (1), 19–31.

Stynder, D. 2009. South african later stone age herders and hunter-gatherers were a single biological population, *Journal of Archaeological Science* **36**, 798–806.

Stynder, D., Ackermann, R., and Sealy, J. 2007. Craniofacial variation and population continuity during the south african holocene, *American Journal of Physical Anthropology* **134**, 489–500.

Trombetta, B., D'Atanasio, E., Massaia, A., Ippoliti, M., Coppa, A., Candilio, F., Coia, V., Russo, G., Dugoujon, J.-M., Moral, P., Akar, N., Sellitto, D., Valesini, G., Novelletto, A., Scozzari, R., and Cruciani, F. 2015.

Phylogeographic refinement and large scale genotyping of human y chromosome haplogroup e provide new insights into the dispersal of early pastoralists in the african continent, *Genome Biology and Evolution* **7** (7), 1940–1950.

Uchiyama, Y., Sakaguchi, M., Terabayashi, T., Inenaga, T., Inoue, S., Kobayashi, C., Oshima, N., Kiyonari, H., Nakagata, N., Sato, Y., Sekiguchi, K., Miki, H., Araki, E., Fujimura, S., Tanaka, S. S., and Nishinakamura, R. 2010. Kif26b, a kinesin family gene, regulates adhesion of the embryonic kidney mesenchyme, *Proceedings of the National Academy of Sciences of the United States of America* **107** (20), 9240–9245.

Underhill, P., Shen, P., Lin, A., Jin, L., Passarino, G., Yang, W., Kauffman, E., Bonné-Tamir, B., Bertranpetit, J., Francalacci, P., Ibrahim, M., Jenkins, T., Kidd, J., Mehdi, S., Seielstad, M., Wells, R., Piazza, A., Davis, R., Feldman, M., Cavalli-Sforza, L., and Oefner, P. 2000. Y chromosome sequence variation and the history of human populations, *Nature Genetics* **26** (3), 358–361.

Van der Auwera, G., Carneiro, M., Hartl, C., Poplin, R., Del Angel, G., Levy-Moonshine, A., Jordan, T., Shakir, K., Roazen, D., Thibault, J., Banks, E., Garimella, K., Altshuler, D., Gabriel, S., and M.A., D. 2013. From fastq data to high-confidence variant calls: the genome analysis toolkit best practices pipeline, *Current protocols in bioinformatics* **43** (1), 11–10.

Van Der Merwe, N. 1982. Carbon isotopes, photosynthesis, and archaeology, *American Scientist* **70** (6), 596–606.

Van Oven, M. 2015. Phylotree build 17: Growing the human mitochondrial dna tree, *Forensic Science International: Genetics Supplement Series* **5**, e392–e394.

van Oven, M., Van Geystelen, A., Kayser, M., Decorte, R., and Larmuseau, M. 2014. Seeing the wood for the trees: A minimal reference phylogeny for the human y chromosome, *Human Mutation* **35** (2), 187–191.

Veeramah, K., Wegmann, D., Woerner, A., Mendez, F., Watkins, J. C., Destro-Bisol, G.and Soodyall, H., Louie, L., and Hammer, M. 2012. An early divergence of khoesan ancestors from those of other modern humans is supported by an abc-based analysis of autosomal resequencing data, *Molecular Biology and Evolution* **29** (2), 617–630.

Vicente, M., Jakobsson, M., Ebbesen, P., and Schlebusch, C. 2019. Genetic affinities among southern africa hunter-gatherers and the impact of admixing farmer and herder populations, *Molecular Biology and Evolution* **36** (9), 1849–1861.

Vicente, M., Lankheet, I., Russell, T., Hollfelder, N., Coetzee, V., Soodyall, H., Jongh, M., and Schlebusch, C. 2021. Male-biased migration from east africa introduced pastoralism into southern africa, *BMC Biology* **19** (1), 1–16.

Wall, J., Lohmueller, K., and Plagnol, V. 2009. Detecting ancient admixture and estimating demographic parameters in multiple human populations, *Molecular Biology and Evolution* **26** (8), 1823–1827.

Wang, K., Goldstein, S., Bleasdale, M., Clist, B., Bostoen, K., Bakwa-Lufu, P., Buck, L., Crowther, A., Dème, A., McIntosh, R., Mercader, J., Ogola, C., Power, R., Sawchuk, E., Robertshaw, P., Wilmsen, E., Petraglia, M., Ndiema, E., Manthi, F., Krause, J., Roberts, P., Boivin, N., and Schiffels, S. 2020. Ancient genomes reveal complex patterns of population movement, interaction, and replacement in sub-saharan africa, *Science Advances* **6** (24), eaaz0183.

Wang, S., Lachance, J., Tishkoff, S., Hey, J., and Xing, J. 2013. Apparent variation in neanderthal admixture among african populations is consistent with gene flow from non-african populations, *Genome Biology and Evolution* **5** (11), 2075–2081.

Weir, B. S. and Cockerham, C. C. 1984. Estimating f-statistics for the analysis of population structure, *Evolution* **38** (6), 1358–1370.

Weissensteiner, H., Pacher, D., Kloss-Brandstätter, A., Forer, L., Specht, G., Bandelt, H.-J., Kronenberg, F., Salas, A., and Schönherr, S. 2016. Haplogrep 2: mitochondrial haplogroup classification in the era of high-throughput sequencing, *Nucleic Acids Research* **44** (W1), W58–W63.

Wu, S., Wang, X., Dai, S., Zhang, G., Zhou, J., and Shen, Y. 2022. A novel missense mutation in greb1l identified in a three-generation family with renal hypodysplasia/aplasia-3, *Orphanet Journal of Rare Diseases* **17** (1).

Xu, L., He, W., Tai, S., Huang, X., Qin, M., Liao, X., Jing, Y., Yang, J., Fang, X., Shi, J., and Jin, N. 2025. Vcf2dis: an ultra-fast and efficient tool

to calculate pairwise genetic distance and construct population phylogeny from vcf files, *GigaScience* **14**, giaf032.

Yang, D. Y., Eng, B., Waye, J. S., Dudar, J. C., and Saunders, S. R. 1998. Improved dna extraction from ancient bones using silica-based spin columns, *American Journal of Physical Anthropology: The Official Publication of the American Association of Physical Anthropologists* **105** (4), 539–543.

Zeberg, H., Jakobsson, M., and Paabo, S. 2024. The genetic changes that shaped neandertals, denisovans, and modern humans, *Cell* **187** (5), 1047–1058.
