# Supplementary material for: Homo sapiens-specific evolution unveiled by ancient southern African genomes
Source: Nature. 2025 Dec 3;650(8100):156–63. doi: 10.1038/s41586-025-09811-4 (PMC12872451; doi:10.1038/s41586-025-09811-4)
Supplement: Supplementary file 2 — Reporting Summary [file 41586_2025_9811_MOESM2_ESM.pdf]

nature portfolio

Corresponding author(s): Mattias Jakobsson

Last updated by author(s): Oct 16, 2025

Reporting Summary

Nature Portfolio wishes to improve the reproducibility of the work that we publish. This form provides structure for consistency and transparency in reporting. For further information on Nature Portfolio policies, see our Editorial Policies and the Editorial Policy Checklist.

Statistics

For all statistical analyses, confirm that the following items are present in the figure legend, table legend, main text, or Methods section.

n/a	Confirmed	
☐	☒	The exact sample size (*n*) for each experimental group/condition, given as a discrete number and unit of measurement
☐	☒	A statement on whether measurements were taken from distinct samples or whether the same sample was measured repeatedly
☐	☒	The statistical test(s) used AND whether they are one- or two-sided<br>*Only common tests should be described solely by name; describe more complex techniques in the Methods section.*
☒	☐	A description of all covariates tested
☒	☐	A description of any assumptions or corrections, such as tests of normality and adjustment for multiple comparisons
☐	☒	A full description of the statistical parameters including central tendency (e.g. means) or other basic estimates (e.g. regression coefficient) AND variation (e.g. standard deviation) or associated estimates of uncertainty (e.g. confidence intervals)
☐	☒	For null hypothesis testing, the test statistic (e.g. *F*, *t*, *r*) with confidence intervals, effect sizes, degrees of freedom and *P* value noted<br>*Give P values as exact values whenever suitable.*
☒	☐	For Bayesian analysis, information on the choice of priors and Markov chain Monte Carlo settings
☒	☐	For hierarchical and complex designs, identification of the appropriate level for tests and full reporting of outcomes
☒	☐	Estimates of effect sizes (e.g. Cohen's *d*, Pearson's *r*), indicating how they were calculated

Our web collection on statistics for biologists contains articles on many of the points above.

Software and code

Policy information about availability of computer code

Data collection

The majority of the samples (labelled “flo”) were housed by the National Museum of Bloemfontein at Florisbad Quarternary Research Station, Free State, South Africa. Permission to sample human remains were approved by Eastern Cape Provincinalheritage Resources Authority (no. 2/2APM-PERMIT/15/03/002-) and Heritage Western Cape (no. 14120409GT0812E) and permission for export and destructive sampling was approved by the South African Heritage Resources Agency (SAHRA no. 1987). Remaining samples (labelled “plo” and “tob”) were housed at the School of Anatomical Sciences and Evolutionary Studies Institute, University of Witwatersrand, Gauteng, South Africa. Permission for sampling (SAHRA no. 1934) and export (SAHRA no. 1935) were approved by the South African Heritage Resources Agency. Sampling was done on site and the skeletal remains were immediately returned.

Data analysis

A full description of all software and respective packages used for data analysis can be found in the Supplementary Information document and are publicly available. For genomic reads mapping: Burrows-Wheller Aligner (BWA, v. 0.7.13); genomic libraries merging: samtools v. 1.5. mtDNA contamination estimates: contamMix (1.0-10); X-chromosome contamination estimates: ANGSD v. 0.902; autosomal contamination estimates: verifyBamID v.1.1.2. Mt haplogroup assignement: Haplogrep v. 2.1.16 and PhyloTree mtDNA tree Build 17 (18 Feb 2016); Y chromosome haplogroup assignement: ISOGG (10, April 21, 2016) SNPs called using samtools v. 1.5. Pseudohaploid genomic dataset management (including LD pruning and datasets merging): PLINK v. 1.9. . PCA: Plink v.19 (--pca command), smartpca v.10210 (EIGENSOFT package); model-based clustering analysis: ADMIXTURE v. 1.3.0 and PONG v. 1.5. f -statistics: python2 script POPSTATS (https://github.com/pontussk/popstats). Diploid genotype calling: GATK v3.5.0 and snpAD. Diploid genomic dataset management (including SNP selection) Vcftools v. 0.1.16 and Plink v. 1.9. Runs of Homozygosity: Plink v. 1.9. Pairwise Sequentially Markovian Coalescent (PSMC) implemented on MSMC v. 0.1.0. Phenotypic variation analysis: ANGSD v. 0.933. Results visualization and plot generation: R v. 3.4.067. Radiocarbon dates calibration: Oxcal v4.4 and IntCal20.

For manuscripts utilizing custom algorithms or software that are central to the research but not yet described in published literature, software must be made available to editors and reviewers. We strongly encourage code deposition in a community repository (e.g. GitHub). See the Nature Portfolio guidelines for submitting code & software for further information.

Data

Policy information about availability of data

All manuscripts must include a data availability statement. This statement should provide the following information, where applicable:

- Accession codes, unique identifiers, or web links for publicly available datasets
- A description of any restrictions on data availability
- For clinical datasets or third party data, please ensure that the statement adheres to our policy

Simons Genome Diversity Project datasets (https://www.simonsfoundation.org/simons-genome-diversity-project/), Human Genome Diversity Project dataset (https://ftp.1000genomes.ebi.ac.uk/vol1/ftp/data_collections/HGDP/), 1000 Genome Project (https://ftp.1000genomes.ebLac.uk/vol1/ftp/data_collections/1000G_2504_high_coverage/). Other comparative modern individuals' genomic data downloaded according to the the references listed in Supplementary Data File 8. Comparative ancient individuals' genomic data downloaded from the European Nucleotide Archive (ENA), under the accession numbers provided in the references listed in Supplementary Data File 7. Human reference genome build 37 (hs37d5) (https://ftp.1000genomes.ebi.ac.uk/voll/ftp/technical/reference/phase2_reference_assembly_sequence/).

All the generated high-coverage sequence data are available as bamfiles of aligned reads at the European Nucleotide Archive (ENA) under the accession number PRJEB98562. Pseudohaploid genotypes are available as transposed PLINK files at 10.5281/zenodo.17295109

Human research participants

Policy information about studies involving human research participants and Sex and Gender in Research.

Reporting on sex and gender	N/A
Population characteristics	N/A
Recruitment	N/A
Ethics oversight	N/A

Note that full information on the approval of the study protocol must also be provided in the manuscript.

Field-specific reporting

Please select the one below that is the best fit for your research. If you are not sure, read the appropriate sections before making your selection.

☒ Life sciences ☐ Behavioural & social sciences ☐ Ecological, evolutionary & environmental sciences

For a reference copy of the document with all sections, see nature.com/documents/nr-reporting-summary-flat.pdf

Life sciences study design

All studies must disclose on these points even when the disclosure is negative.

Sample size	Genomic and radiocarbon data from 28 ancient individuals from South Africa were analysed in this study. The sample size was dependent on the availability of human remains dating to the Stone Age and Iron Age from southern Africa, with preserved and retrievable ancient DNA sequences. These specimens are very rare, given the poor molecular preservation of human remains from this period in that region. Given the millions of genetic variants analysed for each individual, information about the genetic history can be retrieved.
Data exclusions	Reads shorter than 35 base pairs (bp), with more than 10% mismatch from the Reference genome and mapping quality score below 30 were discarded while preparing bamfiles for merged genomic libraries data. For samples not subjected to Uracil-Specific Excision Reagent (USER) treatment, 10 bp at the reads ends were excluded. For samples with partial treatment (comparative dataset) 2 bp were trimmed off of the reads ends. For analyses, minimum mapping and read qualities were set to 30. Pseudohaploid dataset was generated by randomly drawing one read at each SNP site, and that allele assumed to be homozygous. LD pruning for ADMIXTURE resulted in a reduction of the number of analysed SNPs (originally 6,116,165) to 5,616,975. When pairs of first-degree relatives (of comparative populations) were found, the individual with lower genomic coverage of the pair was excluded from analysis. Diploid dataset was generated with samples with a minimum of 7.2x genomic coverage. For MSMC's implementation of PSMC', minimum mapping quality of 30 and minimum genotype quality of 30 were used. For phenotypic analysis, genotype likelihoods were computed based on minimum mapping and read quality of 30 and read depth of 5.
Replication	107 DNA extracts and 558 genomic libraries were generated (a detailed break-down per individual is reported in Supplementary Data File 1), and 1296 rounds of sequencing were performed (a detailed break-down for each library is reported in Supplementary Data File 1) as replication. All these sequencing replicates were successful. Data was merged for downstream analysis after confirming similar results, as expected of different replicates of the same individual's genomic data, such as contamination estimates, mitochondrial haplogroup. Thousands to millions of genetic markers were then analysed as an internal replication of the results. Detailed description of the methods used, including samples included in the dataset, software employed and respective parameters is available in the Supplementary Information.

Randomization	Randomization is not applicable to this study. Samples were grouped according to the archaeological site of origin and radiocarbon date. Groups are validated by verifying genetic affinities among its several individuals.
Blinding	Blinding is not applicable to this study. The archaeological context, including site location and estimated date, of each individual analysed is known prior to sampling and analysis, as these are relevant for conceiving the study.

Reporting for specific materials, systems and methods

We require information from authors about some types of materials, experimental systems and methods used in many studies. Here, indicate whether each material, system or method listed is relevant to your study. If you are not sure if a list item applies to your research, read the appropriate section before selecting a response.

Materials & experimental systems

n/a	Involved in the study
☒	☐ Antibodies
☒	☐ Eukaryotic cell lines
☐	☒ Palaeontology and archaeology
☐	☒ Animals and other organisms
☒	☐ Clinical data
☒	☐ Dual use research of concern

Methods

n/a	Involved in the study
☒	☐ ChIP-seq
☒	☐ Flow cytometry
☒	☐ MRI-based neuroimaging

Palaeontology and Archaeology

Specimen provenance	Samples (labelled "flo") from human remains were collected at the National Museum of Bloemfontein at Florisbad Quarternary Research Station, Free State, South Africa. Permission to sample human remains were approved by Eastern Cape Provincinal heritage Resources Authority (no. 2/2APM-PERMIT/15/03/002-) and Heritage Western Cape (no. 14120409GT0812E) and permission for export and destructive sampling was approved by the South African Heritage Resources Agency (SAHRA no. 1987). Samples (labelled ”plo” and ”tob”) were collected at the School of Anatomical Sciences at Wits Medical School University of Witwatersrand, Gauteng, South Africa. Permission for sampling (SAHRA no. 1934) and export (SAHRA no. 1935) were approved by the South African Heritage Resources Agency. Sampling was done on site and the skeletal remains were immediately returned.
Specimen deposition	National Museum of Bloemfontein, School of Anatomical Sciences at Wits Medical School University of Witwatersrand
Dating methods	All samples without prior radiocarbon date were directly radiocarbon dated using accelerator mass spectrometry (AMS) at the Beta Analytic Carbon dating laboratory. Radiocarbon calibration was performed using OxCal v.4.4 and the IntCal20 dataset.

☒ Tick this box to confirm that the raw and calibrated dates are available in the paper or in Supplementary Information.

Ethics oversight	Sampling permits were obtained from the South African Heritage Resources Agency (SAHRA)

Note that full information on the approval of the study protocol must also be provided in the manuscript.

Animals and other research organisms

Policy information about studies involving animals; ARRIVE guidelines recommended for reporting animal research, and Sex and Gender in Research

Laboratory animals	n/a
Wild animals	n/a
Reporting on sex	The sex of the individuals for which archaeological remains were analysed was determined based on the ratio of coverage of the X chromosome and Y chromosome relative to the autosomes.
Field-collected samples	n/a
Ethics oversight	n/a

Note that full information on the approval of the study protocol must also be provided in the manuscript.
